# Complex Loop Dynamics Underpin Activity, Specificity, and Evolvability in the (βα)_8_ Barrel Enzymes of Histidine and Tryptophan Biosynthesis

**DOI:** 10.1021/jacsau.2c00063

**Published:** 2022-04-04

**Authors:** Adrian Romero-Rivera, Marina Corbella, Antonietta Parracino, Wayne M. Patrick, Shina Caroline Lynn Kamerlin

**Affiliations:** † Department of Chemistry—BMC, Uppsala University, BMC Box 576, S-751 23 Uppsala, Sweden; ‡ Centre for Biodiscovery, School of Biological Sciences, Victoria University of Wellington, 6012 Wellington, New Zealand

**Keywords:** (βα)_8_-barrel enzymes, protein evolution, catalytic promiscuity, computational enzymology, empirical valence bond, enhanced sampling

## Abstract

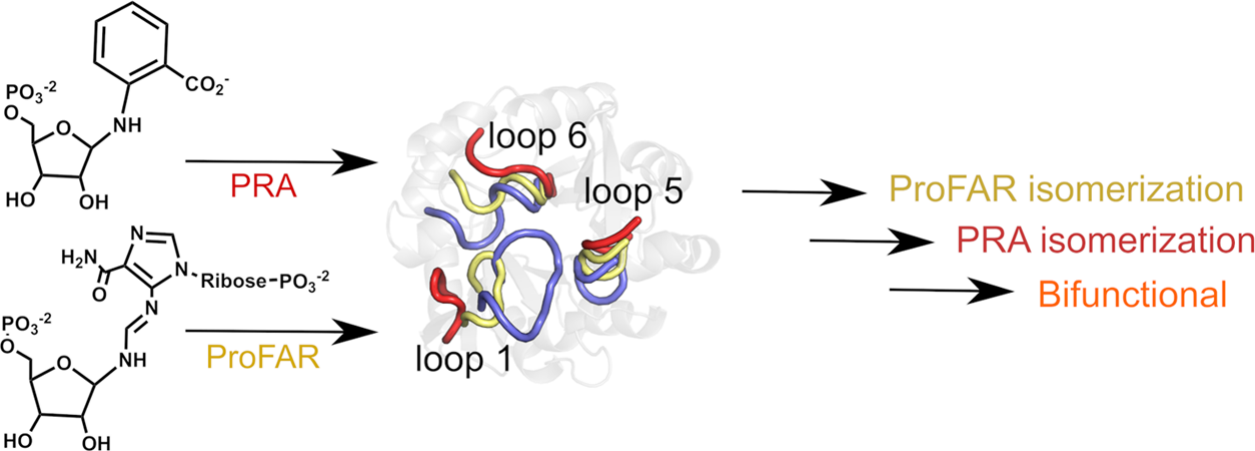

Enzymes are conformationally dynamic, and their dynamical properties play an important role in regulating their specificity and evolvability. In this context, substantial attention has been paid to the role of ligand-gated conformational changes in enzyme catalysis; however, such studies have focused on tremendously proficient enzymes such as triosephosphate isomerase and orotidine 5′-monophosphate decarboxylase, where the rapid (μs timescale) motion of a single loop dominates the transition between catalytically inactive and active conformations. In contrast, the (βα)_8_-barrels of tryptophan and histidine biosynthesis, such as the specialist isomerase enzymes HisA and TrpF, and the bifunctional isomerase PriA, are decorated by multiple long loops that undergo conformational transitions on the ms (or slower) timescale. Studying the interdependent motions of multiple slow loops, and their role in catalysis, poses a significant computational challenge. This work combines conventional and enhanced molecular dynamics simulations with empirical valence bond simulations to provide rich details of the conformational behavior of the catalytic loops in HisA, PriA, and TrpF, and the role of their plasticity in facilitating bifunctionality in PriA and evolved HisA variants. In addition, we demonstrate that, similar to other enzymes activated by ligand-gated conformational changes, loops 3 and 4 of HisA and PriA act as gripper loops, facilitating the isomerization of the large bulky substrate ProFAR, albeit now on much slower timescales. This hints at convergent evolution on these different (βα)_8_-barrel scaffolds. Finally, our work reemphasizes the potential of engineering loop dynamics as a tool to artificially manipulate the catalytic repertoire of TIM-barrel proteins.

## Introduction

Enzymes are conformationally dynamic systems, and their dynamical properties are clearly connected to their biological function.^1−7^ Further, conformational dynamics, in particular, that of decorating loops that cover enzyme active sites, can regulate substrate selectivity, the evolution of new activities, and potentially also turnover rates.^3,4,8−29^ Because of this, enzyme conformational ensembles can, in principle, be engineered to allow enzymes to acquire new catalytic functions and/or physiochemical properties.^23,24,29−33^ Therefore, there is substantial interest in understanding loop dynamics and its impact on selectivity and catalysis, to improve our ability to manipulate enzyme conformational ensembles for engineering purposes.

There have been extensive studies of a range of enzymes, such as triosephosphate isomerase (TPI),^34,35^ orotidine 5′-monophosphate decarboxylase, (OMPDC)^36^ glycerol-3-phosphate dehydrogenase (GPDH),^37,38^ 1-deoxy-d-xylulose-5-phosphate reductoisomerase,^39,40^ and β-phosphoglucomutase,^41^ that are activated by ligand-gated conformational changes. Specifically, interactions between a key “gripper” loop decorating the active site and the nonreactive phosphodianion groups of these enzymes’ substrates trigger substantial conformational catalytically critical changes in the gripper loop, facilitating energetically unfavorable transitions from inactive open to active closed conformations.

It is noteworthy that several of these enzymes have TIM-barrel ((βα)_8_-barrel) folds. Most if not all TIM-barrel proteins possess decorating loops,^8,12,21,42^ the conformational diversity of which likely plays an important role in regulating specificity and function.^15,18^ These flexible loops can vary in length,^8^ but are typically used to bind substrate, and to sequester the active site from solvent by closing over the active site.^43^ However, these well-characterized examples of proteins activated by ligand-gated conformational changes all focus on the roles and importance of single loops, such as gripper loop 6 in TPI. Studying the ligand-gated motion of a single loop can already pose substantial challenges;^44^ systems with multiple active site loops undergoing substantial conformational changes are even more complex and therefore understudied.

**Figure 1:**
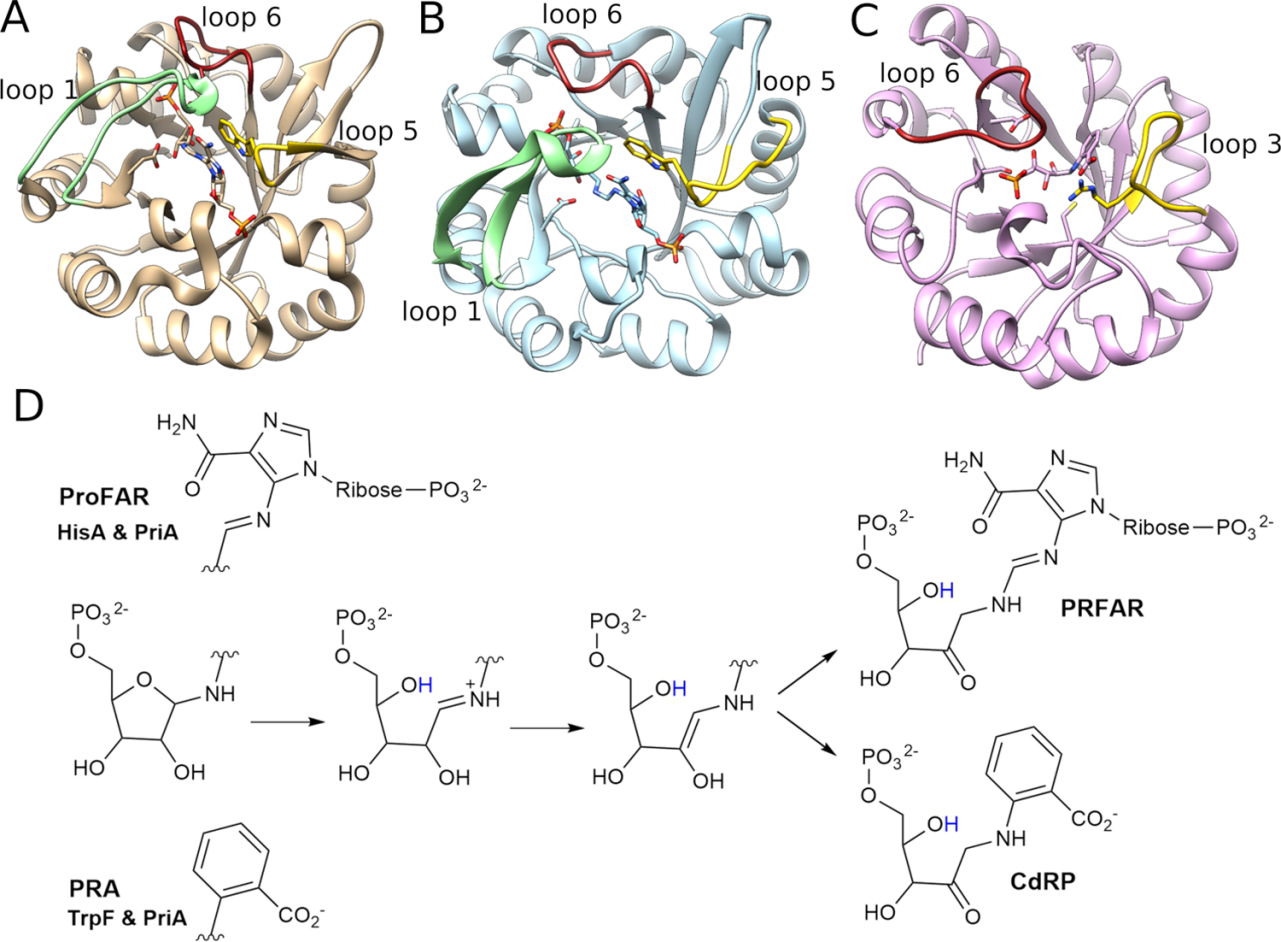
Tertiary structures of (A) *Se*HisA in complex with ProFAR (PDB ID: 5A5W(54,55)), (B) *Mt*PriA in complex with PRFAR (PDB ID: 3ZS4(55)), and (C) *Tm*TrpF in complex with product analogue rCdRP (PDB ID: 1LBM(49)). Loop 1 (residues 11–29 in HisA, 15–33 in *Mt*PriA), loop 5 (residues 142–147 in *Se*HisA, 141–151 in PriA and 30–39 in *Tm*TrpF) (loop 3), and loop 6 (residues 172–182 in *Se*HisA, 172–181 in *Mt*PriA and 128–139 in *Tm*TrpF) are highlighted in light green, yellow, and dark red, respectively. Loop 1 in *Tm*TrpF is short (four residues); thus, no corresponding loop is annotated on this panel. For clarity, N7D and A176D reversions were applied to the structure of *Se*HisA in complex with ProFAR (these reversions were also applied in our simulations). (D) Proposed mechanism for the Amadori rearrangement leading to the isomerization of substrates ProFAR and PRA by the different enzymes.^49^

We have sought to address this knowledge gap by studying active site loop dynamics in the (βα)_8_-barrels of tryptophan and histidine biosynthesis. The enzymes HisA, TrpF, and PriA are model systems for the evolution of enzyme specificity and activity (Figure 1).^18,45−48^ HisA catalyzes the isomerization of *N*′-[(5′-phosphoribosyl)-formimino]-5-aminoimidazole-4-carboxamide-ribonucleotide (ProFAR) into *N*′-[(5′-phosphoribulosyl)-formimino]-5-aminoimidazole-4-carboxamide-ribonucleotide (PRFAR). TrpF catalyzes the same Amadori rearrangement on *N*-(5′-phosphoribosyl)anthranilate (PRA), producing 1-(2-carboxy-phenylamino)-1′-deoxyribulose-5′-phosphate (CdRP). This rearrangement proceeds via a Schiff acid–base mechanism, utilizing aspartate (and in the case of TrpF) cysteine residues as acid–base pairs.^49^

Many actinobacteria lack the *trp*F gene, but instead possess a gene for a naturally bifunctional isomerase, PriA.^50^ The PriA from *Mycobacterium tuberculosis* (*Mt*PriA) has been well characterized, with *k*_cat_/*K*_M_ values of ∼10^4^ M^–1^ s^–1^ for HisA activity and ∼10^5^–10^6^ M^–1^ s^–1^ for TrpF activity.^15,51^ In addition, promiscuous TrpF activity has been detected on ancestral and extant specialist HisA enzymes, with *k*_cat_ values ranging from 10^–4^ to 10^–2^ s^–1^.^52^ HisA has also been converted into TrpF by directed evolution,^53^ and in serial passaging experiments with *Salmonella enterica*,^46^ where 3,000 generations of laboratory evolution yielded an extensive suite of mutations in the ProFAR-specific *S. enterica* HisA (*Se*HisA) resulting in specialist HisA enzymes, specialist TrpF enzymes, and PriA-like bifunctional enzymes.

As shown in Figure 1, HisA and PriA are decorated by three long catalytic loops, loops 1, 5, and 6 (or two analogous loops in the case of TrpF, which has lost most of loop 1).^15,18^ Of these, loop 5 carries key residues that are important for substrate binding, loop 6 carries the catalytically important aspartic acid side chain, and position 15 of loop 1 is important for interaction with substrate PRA.^18,45,54^ In the case of PriA, structural analysis^15^ has suggested that bifunctionality is facilitated by transitions between a “knot-like” pro-ProFAR conformation of loop 5, with W145 pointing “in” toward the substrate ProFAR (PDB ID: 3ZS4(55)), and a pro-PRA β-hairpin conformation of loop 5, with R143 pointing in toward the substrate PRA (PDB ID: 2Y85(15,55)). The evolved *Se*HisA variants generated by Näsvall et al.^46^ have also been the subject of detailed structural and biochemical analyses.^18^ As with PriA,^15^ this analysis indicated that bifunctionality is driven by competition between not just the substrates ProFAR and PRA but also between structurally distinct conformations of loops 1 and 5, in particular^18^ (Figure S1).

**Figure 2:**
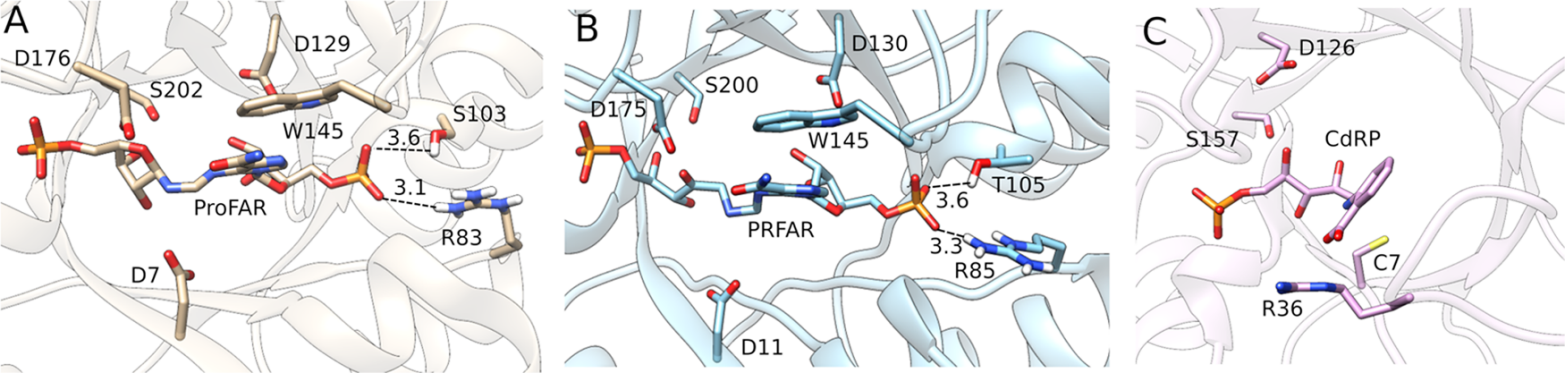
Comparison of the active sites of (A) *Se*HisA in complex with substrate ProFAR (PDB ID: 5A5W(54,55)), (B) *Mt*PriA in complex with product PRFAR (PDB ID: 3ZS4(55)), and (C) *Tm*TrpF in complex with product analogue rCdRP (PDB ID: 1LBM(49,55) ). Highlighted are key catalytic residues, including the catalytic active site aspartic acid (D176 in *Se*HisA, D175 in *Mt*PriA, and D126 in *Tm*TrpF), the active site tryptophan that forms stacking interactions with the larger substrate ProFAR in *Se*HisA and *Mt*PriA (W145 in both enzymes), as well as the gripper residues that interact with the distal phosphate group of the larger substrate/product in *Se*HisA and *Mt*PriA (R83 and S103 in *Se*HisA and R85 and T105 in *Mt*PriA). Key hydrogen bonding interactions are also highlighted, using the distances (Å) found in the corresponding crystal structures. For clarity, N7D and A176D reversions were applied in *Se*HisA in complex with substrate ProFAR (these reversions were also applied in our simulations).

Although isomerization of both ProFAR and PRA proceeds through the same Amadori rearrangement (Figure 1), ProFAR is a much larger molecule containing a second phosphate group. This group forms hydrogen bonds with the side chains of R83 from loop 3 and S103 from loop 4 of *Se*HisA and with the corresponding side chains of R85 and T105 in *Mt*PriA (Figure 2). Although neither of these residues are on the primary mobile loops of either enzyme (Figure 1), these interactions are very similar to analogous interactions in other enzymes activated by ligand-gated conformational changes,^34−41^ suggesting that loops 3 and 4 may similarly act as “gripper loops”, allowing these enzymes to attain relevant catalytically active conformations for the isomerization of the larger substrate. This effect would not be present when the smaller substrate PRA is bound to the active site, as it lacks a nonreactive phosphodianion group to interact with these loops.

Here, we combine conventional molecular dynamics (MD), enhanced sampling, and empirical valence bond (EVB) simulations to present a comprehensive computational study of a number of wild-type and variant forms of *Se*HisA, *Mt*PriA, and the *Thermotoga maritima* TrpF (*Tm*TrpF). All variants studied in this work, and the corresponding structures used, are summarized in Table S1. We chose these systems because high-quality structural data were available in unliganded and ligand-bound forms. For *Se*HisA, we have studied the unliganded and substrate-bound forms,^54^ as well as key *Se*HisA variants from ref (18) that were selected based on their specificity patterns (specialists vs generalists, Table S2). *Mt*PriA and *Tm*TrpF were similarly selected on the basis of structural data in both unliganded and product (PRFAR), or product analogue (rCdRP), bound forms, respectively (Table S1).

Prior simulations have emphasized that even studying the motion of one large dominating conformational change is computationally nontrivial,^22,44,56^ and the current systems involve the interdependent conformational rearrangements of multiple loops simultaneously. Our simulations of HisA, PriA, and TrpF (1) provide rich details of the conformational behavior of the catalytic loops in the different systems and (2) provide insight into the link between conformational dynamics, catalytic activity, and functional evolution in the different enzymes.

## Methodology

Methodological details are presented here in brief. Full details of all simulations and any nonconventional parameters used are provided in the Supporting Information.

### System Preparation for Molecular Dynamics Simulations

Simulations were performed on wild-type *Se*HisA, *Mt*PriA, and *Tm*TrpF, as well as relevant enzyme variants, in both their unliganded forms and in complex with various ligands (substrates ProFAR and PRA and, in the case of the enhanced sampling simulations, products PRFAR and CdRP). A total of 14 crystal structures were used to generate starting points for these simulations, and all simulations performed, as well as the structures, are summarized in Table S1. Where present, the D7N, D11N, and D176A substitutions were reverted to wild type using the Dunbrack 2010 Rotamer Library,^57^ as implemented in UCSF Chimera, v. 1.14.^58^ Missing regions in the catalytic loops were reconstructed using Modeller v. 9.23.^59^ The catalytic aspartic acid side chain in the active site of each enzyme (D176 in HisA, D175 in PriA and D126 in TrpF) was kept protonated in line with the mechanism shown in Figure 1. All other residues (except H50 in PriA, which was doubly protonated) were kept in their default protonation states at physiological pH determined by the use of PROPKA 3.1,^60^ and visual inspection. The substrates ProFAR and PRA were manually placed into the relevant active sites in the same conformation as found in the structure of the HisA wild-type enzyme in complex with ProFAR. In the case of PRA (Figure 1D), the substrate was placed into the relevant active sites by manual overlay of the reactive part of PRA with the reactive part of ProFAR, and with the carboxylate group of PRA keeping key interactions with active site residues. Partial charges for ligands ProFAR, PRA, PRFAR, and CdRP were calculated using the standard restrained electrostatic potential (RESP) protocol using Antechamber v. 17.3,^61^ and based on the vacuum electrostatic potential calculated at the HF/6-31G(d) level of theory, using Gaussian 09 Rev. E.01.^62^ All other simulation parameters were described using the general Amber force field 2 (GAFF2)^63^ (see Tables S3–S6). Finally, to keep the substrate stably bound in the enzyme active sites, weak distance restraints were applied to protein–substrate distances in the conventional molecular dynamics (MD) simulations, as described in Table S7.

### Conventional Molecular Dynamics Simulations

Conventional MD simulations were performed using the CUDA version of the PMEMD module of the AMBER 16 simulation package.^64^ The protein, ligands, and solvent were described using the ff14SB force field,^65^ the General AMBER Force Field 2 (GAFF2),^63^ and the TIP3P water model,^66^ respectively. Following initial minimization and equilibration, each system (summarized in Table S1) was subjected to 10 × 500 ns of molecular dynamics simulations controlled by the Langevin thermostat with a collision frequency of 2 ps^–1^,^67^ and the Berendsen barostat with a 1 ps coupling constant.^68^ This led to a cumulative 5 μs of production simulations per system, and a cumulative total of 70 μs of conventional MD simulations over all systems studied (Table S1).

### Enhanced Sampling Molecular Dynamics Simulations

Steered molecular dynamics simulations (sMD) were performed using GROMACS 2018.4 to pull products PRFAR and CdRP out of the active site of the *Se*HisA(dup13–15/D10G/G102A/Q24L) variant, as described in the Supporting Information. System preparation was performed as for the conventional MD simulations, using the same force fields and water models. Following initial minimization and equilibration, 10 × 50 ns production MD simulations were performed on each system. The first 5 ns of production MD were unrestrained, after which an external force with a force constant of 10 kcal mol^–1^ Å^–2^ was applied to pull the product out of the active site. This external force was then released for the last 5 ns of each MD simulation run.

### Empirical Valence Bond Simulations

**Figure 3:**
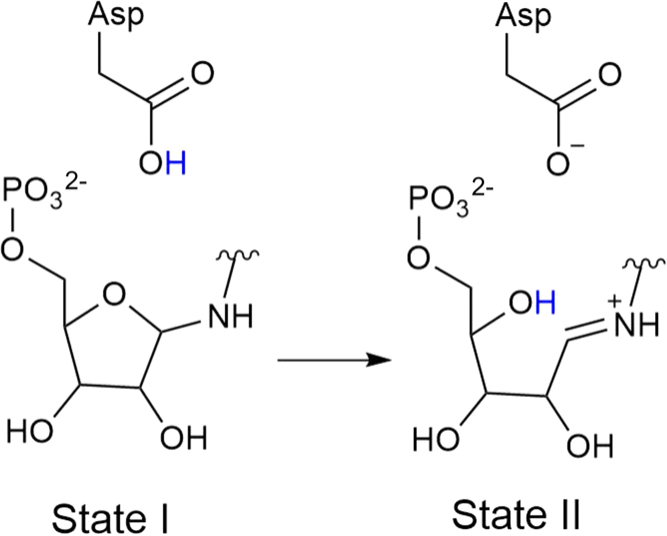
Valence bond states used to describe the ribose ring opening catalyzed by the different enzyme variants studied in this work. Only the ribose moiety of the substrate was placed in the EVB region, with the remainder of the substrate treated purely classically. The corresponding EVB parameters are provided in the Supporting Information and on Zenodo, DOI: 10.5281/zenodo.5893598.

We extended our previous empirical valence bond (EVB) approach^69−73^ to study the enzyme-catalyzed opening of the ribose ring of substrates ProFAR and PRA (Figure 1), as catalyzed by wild-type and variant forms of HisA, PriA, and TrpF. This is the first step of the mechanism shown in Figure 1D, and used the valence bond states shown in Figure 3. Simulations were performed on wild-type *Se*HisA, *Mt*PriA, and *Tm*TrpF as well as selected variants, as described in the Supporting Information. All simulations were performed using the *Q*6 simulation package,^74,75^ using the all-atom optimized potentials for liquid simulations (OPLS-AA) force field.^76^ All EVB parameters and methods necessary to reproduce our work can be found in the Supporting Information, with the full parameters also uploaded to Zenodo (DOI: 10.5281/zenodo.5893598). Each system was simulated using 30 individual replicas, with each replica first equilibrated for 20 ns and the endpoint of that equilibration being used as the starting point for propagating an EVB trajectory. Each EVB free energy perturbation/umbrella sampling (EVB-FEP/US)^69^ simulation was simulated using 51 individual mapping windows of 200 ps of simulation time each, leading to a total of 10.2 ns of simulation time per individual EVB trajectory. The cumulative totals were 600 ns equilibration and 306 ns EVB simulation time per individual system, and a cumulative 12 μs of equilibration and 6.12 μs of EVB simulation time over all 20 systems studied.

### Analysis of Conventional and Enhanced Sampling Molecular Dynamics Simulations

Unless stated otherwise, all analysis of all conventional and enhanced sampling MD simulations was performed using CPPTRAJ.^77^ Hydrogen bonds were defined as formed if the donor–acceptor distance was ≤3.0 Å and the donor–hydrogen-acceptor angle was within 180 ± 45°. Principal component analysis (PCA) was performed by first RMS fitting to a whole protein C_α_ carbon atoms and then performing PCA on the C_α_ carbon atoms of loops 1, 5, and 6, as well as loop 1 for HisA loop 1 elongated systems analysis. Other analyses were performed as described in the Supporting Information.

## Results and Discussion

### Active Site Plasticity and Substrate Binding in the Different Enzymes

*Se*HisA and *Mt*PriA have similar structures, with a C_α_-RMSD of 1.09 Å when PDB IDs 3ZS4 ^55^ and 5A5W ^54,55^ are compared. In contrast, *Tm*TrpF is significantly smaller, with 40 fewer residues. We calculated the mean and standard deviations of the active site volumes of each unliganded enzyme during 10 × 500 ns conventional MD simulations using MDpocket,^78^ obtaining average volumes of 745.4 ± 129.6, 1033.8 ± 217.0, and 1173.5 ± 158.0 Å^3^, for TrpF, PriA, and HisA, respectively (Table S8). The TrpF-active site is more compact than those of PriA and HisA, which have successively larger and more “flexible” active site pockets (using the standard deviation on the volume as a proxy for flexibility). For comparison, the substrates ProFAR and PRA have volumes of 829 and 559 Å^3^, respectively, calculated using Mol_Volume Version 1.0, with default radii of 1.7 Å and a probe sphere of 0.5 Å. This confirms the structural data indicating that the active site pocket of TrpF is too compact to accommodate the larger substrate, ProFAR, leading to the selectivity of this enzyme toward PRA.^79^ Furthermore, the PriA active site is the most flexible of the three, in line with structural data,^15^ indicating that PriA is capable of significantly rearranging its active site (in particular, loop 5 conformation) when accommodating the different substrates ProFAR and PriA (Figure S1).

**Figure 4:**
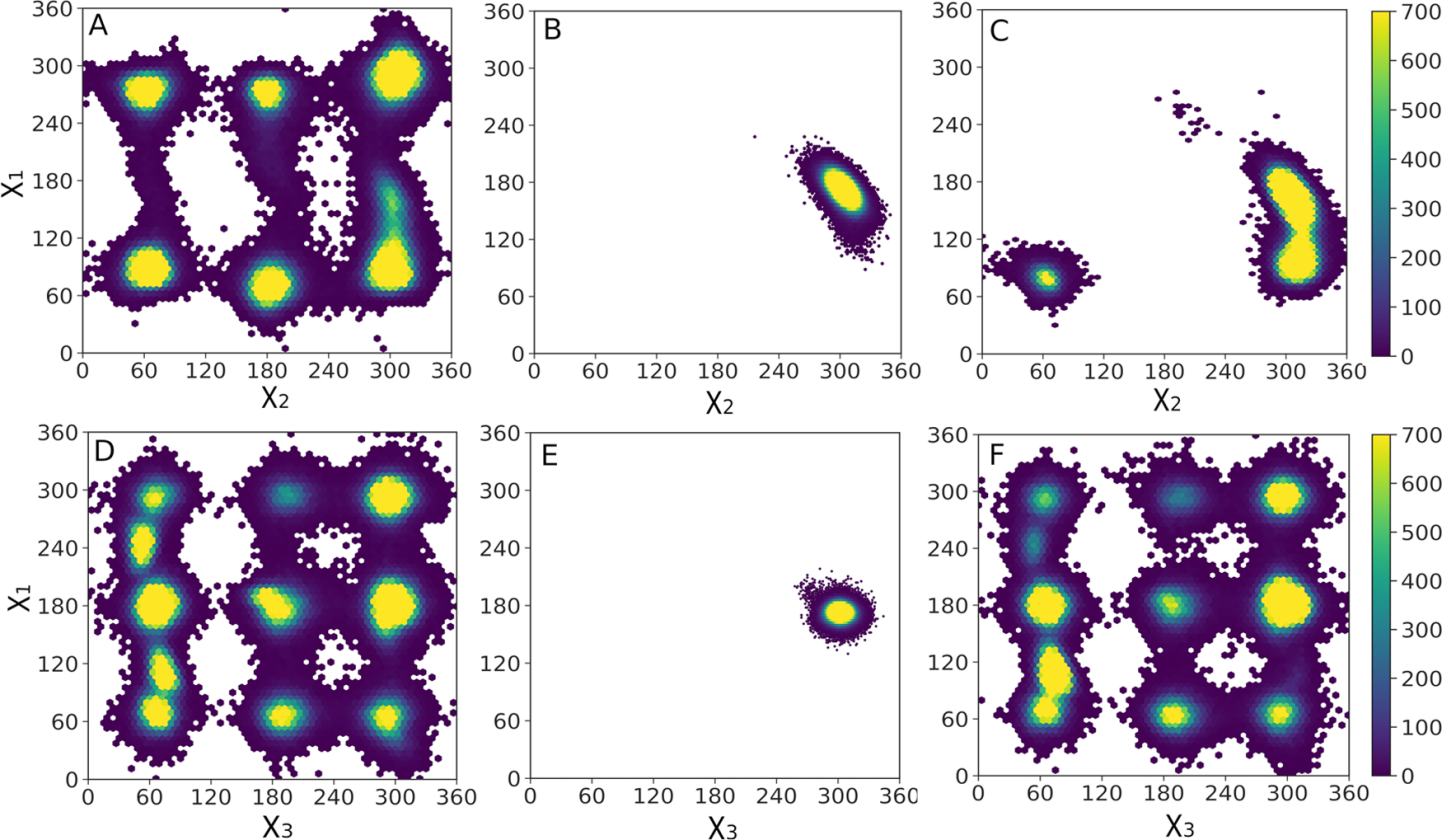
Joint distribution of the side-chain dihedral angles of the side chains of (A–C) W145 and (D–F) R83 in (A, D) unliganded HisA, (B, E) HisA in complex with substrate ProFAR, and (C, F) HisA in complex with substrate PRA. Data were extracted every 5 ps of 10 × 500 ns production simulations of each system, performed as described in the Supporting Information.

Two key active site side chains in HisA and PriA are important for binding ProFAR:^15,18,54,80^ W145, which forms a stabilizing stacking interaction with the substrate, and R83 (R85), which interacts with the second phosphodianion group of the substrate (Figure 2). To explore the conformational diversity of these side chains, we examined their joint dihedral angle distributions in simulations of unliganded HisA and PriA, as well as HisA and PriA in complex with substrates ProFAR and PRA. The corresponding dihedral data are shown in Figures 4 and S2.

These data show that the tryptophan and arginine side chains are both highly conformationally flexible in the unliganded enzymes. However, while ProFAR binding to HisA restricts the conformational space of the arginine side chain on the gripper loop 3 to a catalytically competent position that helps stabilize the bound substrate, in PriA, the R143 side chain is only 4.8 Å from the gripper residue R85 (distance between the two side-chain carbon atoms, based on PDB ID: 3ZS4 ^55^). This creates electrostatic repulsion between the two arginine side chains, destabilizing both loop 5 as well as the interaction between ProFAR and the R85 side chain (Figure S3). In contrast, when PRA is bound to either HisA or PriA, the R83/R85 side chains increase their conformational flexibility, sampling roughly the same conformational space as in the unliganded enzyme (Figure S2). Therefore, the interaction of these residues with the larger substrate ProFAR is likely playing an important role in the ability of these enzymes to bind and isomerize it.

The W145 side chain forms a stabilizing stacking interaction with the substrate and slightly increases its conformational flexibility when the smaller substrate PRA is bound to HisA (note that PRA was placed manually in the active site by overlay with ProFAR), although the flexibility of this side chain is still substantially reduced compared to the unliganded enzyme (Figure 4A). However, in the case of PriA, both the W145 and R143 side chains are conformationally restricted to a catalytically competent position due to a rearrangement of loop 5 upon PRA binding that swaps the position between these two residues compared to when ProFAR is bound to PriA, preventing the electrostatic repulsion between the R85 and R143 side chains that is observed when ProFAR (PRFAR) is bound to the active site (Figure S1).^15^

As noted previously, the R83/R85 side chain is one of a number of key residues on the gripper loop that interact with the distal phosphodianion group of the larger substrate ProFAR, contributing to the stabilization of the substrate in the HisA active site. Hence, in PriA, the loop 5 rearrangement required for ProFAR binding^15^ prevents R85 from gripping the second phosphodianion group, leading to a preference for the isomerization of PRA. These gripper interactions are similar to corresponding interactions in enzymes such as TIM, OMPDC, and GPDH,^34−38^ where interactions with the nonreactive phosphodianion group of the substrate drive catalytically important ligand-gated conformational changes. Here, the interaction between the HisA gripper residues and the remote phosphodianion group of ProFAR appears to be similarly important for maintaining the closed conformation of loop 5, and when this interaction is lost, as in PriA, we see the corresponding opening of loop 5 (Figure S3). This supports the likelihood that HisA and PriA are also activated by ligand-gated conformational changes, albeit with more complex loop dynamics (due to the involvement of not one but three highly mobile and long catalytic loops) than in other previously characterized systems.

### Conformational Dynamics of Key Catalytic Loops of HisA, PriA, and TrpF

To further explore the impact of ProFAR and PRA binding on loop dynamics, we extended our simulations to include TrpF in both its unliganded form, and in complex with PRA (Table S1). We then performed principal component analysis (PCA) to characterize the motion of the catalytic loops during our simulations of each system, similar to prior analysis we have performed on TPI.^44^ The PCA was performed on the mass-weighted Cartesian coordinates of each enzyme compared to the coordinates of the corresponding closed state, allowing us to explore the variation of the conformations of these loops in coordinate space.

**Figure 5:**
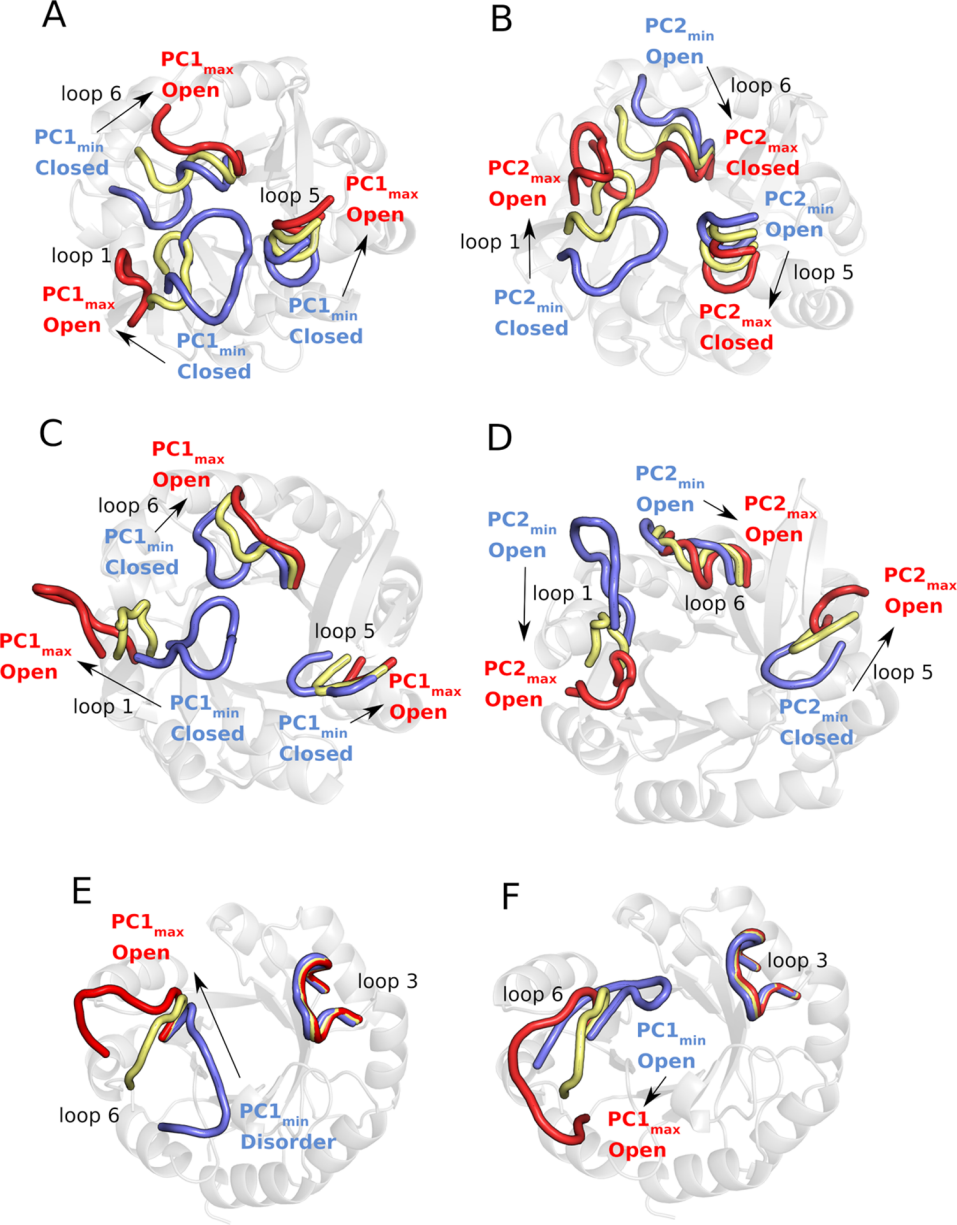
Overview of the variations in the conformational states of key catalytic loops of (A, B) HisA, (C, D) PriA, and (E, F) TrpF in coordinate space, along the first two principal components (A, C, E) PC1 and (B, D, F) PC2 obtained from PCA performed on our conventional MD simulations of each system. PCA was performed relative to the corresponding loop closed states for each enzyme, and the minimum and maximum deviations of each loop from this closed state along each PC are highlighted in blue and red, respectively, with an intermediate state observed in our analysis highlighted in yellow.

Figure 5 shows an overview of structural changes in the catalytic loops along the first two principal components, PC1 and PC2, as well as the minimum and maximum deviations of each of the catalytic loops from the corresponding closed reference state along each PC for each enzyme. PC1 and PC2 make contributions of 41.2, 70.9, and 51.0% and 16.4, 6.0, and 13.9% to the overall variance in each of HisA, TrpF, and PriA, respectively. Significant conformational motion is observed along both PCs; however, PC1 primarily describes loop transitions between closed and open conformational states, whereas PC2 describes conformational variation in the loops during these transitions, including transitions between different open conformations.

**Figure 6:**
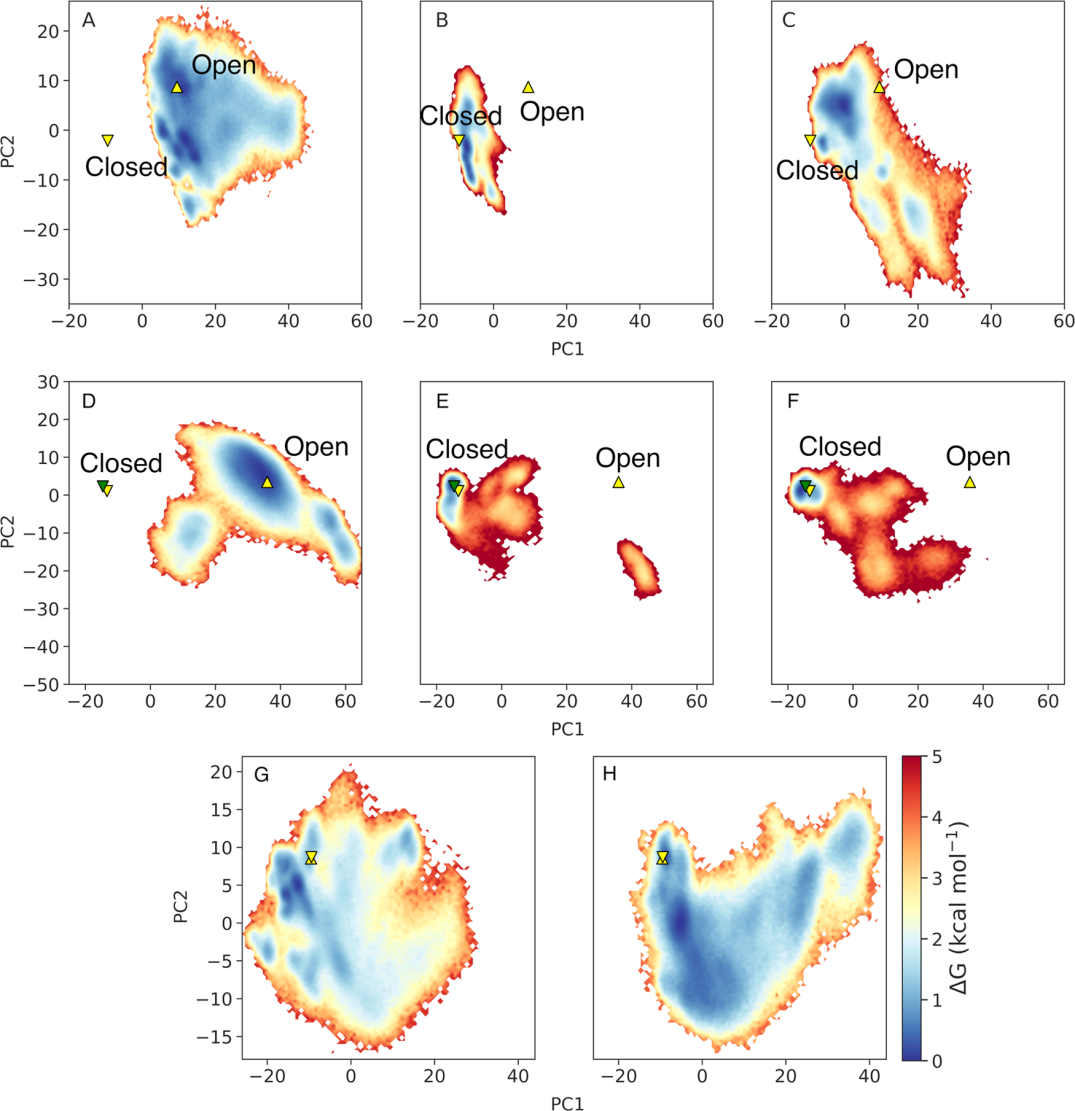
Projected free energy surfaces (kcal mol^–1^) along the first two principal components (PC1 and PC2) obtained from applying Cartesian principal component analysis (PCA) to our conventional MD simulations of (A–C) HisA in its (A) unliganded form, and in complex with (B) ProFAR and (C) PRA, (D–F) PriA in its (D) unliganded form and in complex with (E) ProFAR and (F) PRA, and (G, H) TrpF in its (G) unliganded form and in complex with (H) PRA. Free energies were estimated from the bin populations of the 2D histograms (using 100 bins on each axis), using the formula *G*_i_ = −*k*_B_*T*(*N*_i_/*N*_Max_), where *k*_B_ is the Boltzmann constant, *T* is the temperature at which the simulations were performed (300 K), *N*_i_ is the population of each bin, and *N*_Max_ is the population of the most populated bin. The crystallographic loop open and loop closed states of the enzyme are indicated on this surface using the symbols ▲ and ▼. In the case of HisA, these states are defined based on the loop conformations found in PDB IDs: 5AHE^54^ and 5A5W,^54^ for the unliganded open, ProFAR-bound closed and PRA-bound closed conformations, where the latter two systems have the same crystallographic closed conformation. In the case of PriA, these states are defined based on PDB IDs: 2Y89,^15^3ZS4,^55^ and 2Y85,^15,55^ for the unliganded open, ProFAR-bound closed (yellow triangle), and PRA-bound closed (blue triangle) conformations. For TrpF, the conformations of the catalytic loops are defined based on PDB IDs: 1NSJ ^55,81^ and 1LBM.^49,55^ The overlap of these triangles indicates that the loops are found in similar positions in the crystal structure, irrespective of whether the starting structure is liganded or unliganded. Note that this figure considers the motion of all relevant catalytic loops along these two PC, as illustrated in Figure 5.

We subsequently projected the free energies for each enzyme along the most dominant motions, PC1 and PC2, from simulations of each of HisA, PriA, and TrpF in their unliganded forms as well as in complex with substrates ProFAR and PRA, respectively (where PRA was artificially placed in the HisA active site by manual overlay with the reactive part of ProFAR). This allowed us to compare both the free energy surfaces defined by these two PCs between the different enzymes, and the effect of ligand binding on these surfaces. The resulting combined motion of all key catalytic loops along each principal component is illustrated in Figure 6.

In the unliganded form of each enzyme, the catalytic loops are highly conformationally diverse and explore a range of “wide-open” conformations (Figure 5). This is consistent with our prior simulation studies on both TPI,^44^ and the protein tyrosine phosphatases PTP1B and YopH,^22^ as well as chimeric forms of these enzymes.^82^ However, and consistent with structural data, ProFAR binding to HisA fully restricts the conformational sampling of all three loops (Figure 6B). In the case of PriA, the binding of both ProFAR and PRA also stabilizes the closed conformation of the three active site loops, but still allows for some conformational flexibility in these loops (Figure 6, based on both the topologies of the projected free energy surfaces and the corresponding energies). In sharp contrast, in the liganded form of TrpF, our MD simulations show that PRA is not stable in the active site due to the flexibility of loop 6, which explores transitions toward open conformations, similarly to the unliganded system (Figures 5E,F and 6G,H). This in turn increases the mobility of PRA in the active site even with the restraints applied in the simulations (Table S7), allowing it to sample noncatalytic conformations with Asp-H...PRA ring oxygen distances as high as 10.8 Å (and averaging 4.4 ± 1.4 Å during our simulations). In the crystal structure of the unliganded enzyme (PDB ID: 1NSJ(55,81)), this loop is present in a closed position but with missing density, whereas in our simulations of both the liganded and unliganded form of the enzyme, loop 6 samples open conformations, suggesting that the crystal structure does not reveal the correct closed state to stabilize the substrate PRA in the active site. We note that the structure used for these simulations (PDB ID 1LBM(49,55)) was solved in complex with the product analogue rCdRP. Our simulations suggest that the loop conformations observed in this structure are a conformational state on the trajectory to product release, rather than an ideal conformational state for stabilizing the Michaelis complex.

During our simulations of HisA and PriA, we observed the formation of a stacking interaction between ProFAR and the side chain of W145 (Figure S4), with an average distance of 3.9 ± 0.3 Å and an angle γ = 11.7 ± 5.5° between the center of mass of the ProFAR imidazole ring and the W145 indole ring. Our results thus confirm the role for W145 that was proposed based on experimentally determined structures,^54^ and is consistent with experiments indicating that HisA activity is abolished in *Se*HisA(dup13–15) because the extended conformation of loop 1 blocks this side chain from interacting with ProFAR.^18^

In contrast, in the case of PriA, and again in agreement with prior structural analysis,^15^ we observe two possible conformations of loop 5, depending on which substrate is bound to the active site. When ProFAR is bound, we sample a conformation similar to that observed in wild-type HisA (Figure S1A) with a similar stacking interaction between ProFAR and W145 (Figure S4). However, the loop 5 rearrangement required to optimize the stacking position of W145 with ProFAR creates transient electrostatic repulsion between loop 5 and the rest of the enzyme, making this loop more conformationally dynamic, which we observe in our analysis in the form of an increased standard deviation in the distance and angle of the corresponding stacking interaction (*d* = 4.1 ± 0.6 Å, γ = 19.2 ± 13.9°) (Figures S3 and S4). The greater plasticity of this interaction, in turn, decreases substrate stability in the active site (the ProFAR RMSF increases from 18.4 Å in our simulations of wild-type HisA to 22.2 Å in our simulations of wild-type PriA), and thus the corresponding ProFAR isomerization activity of PriA.

For comparison, in our simulations of PRA-bound PriA, we sampled an active site conformation in which the R143 side chain (loop 5 residue) forms salt bridges with the side chains of D130 and D175, and the arginine acts as a “shield”, dampening the electrostatic repulsion between the D130 side chain and the anthranilate carboxylate group of PRA. This interaction also stabilizes the catalytic aspartic (D175), placing it in an optimal position for catalysis (Figure S5 and Table S9), as shown in previous studies.^15^ This PriA conformation is similar to the “TrpF-active” conformation observed in the *Se*HisA(dup13–15/D10G/Q24L/G102A) crystal structure^18^ (PDB ID: 5AB3,^18,55^Figure S6, with manual placement of PRA in the active site), where the arginine is close to residue D129. We do not observe the formation of a corresponding interaction in our simulations of PRA-bound wild-type HisA, as the negative charge repulsion between PRA and the D129 side chain destabilizes the PRA position in the active site (Figure S7), as well as the stability of loop 6 carrying the key catalytic aspartate (Figure S7). We do, however, observe a similar interaction with the R169 side chain in the *Se*HisA(L169R) variant, with interactions with D129 and, in this case, a salt-bridge interaction with the anthranilate carboxylate group of PRA (Figure S7 and Table S9), consistent with experimental work demonstrating that the introduction of the L169R substitution in HisA induces TrpF activity (Table S2).^46^

Finally, in the case of PRA-bound TrpF, we observe a salt-bridge interaction between E184 and the side chain of R36 on loop 3 (Figure S6 and Table S9), and we can see that as for HisA and PriA, R36 is again acting as a shield avoiding possible negative repulsion interactions between the substrate and the negatively charged side chain. However, we do not observe clear interactions between the R36 side chain and the substrate PRA (Figure S6 and Table S9, with the fraction of simulation time in which this interaction is observed being <0.1).

Overall, we observed that this arginine plays an essential role in the introduction of TrpF activity, by shielding electrostatic repulsion between negatively charged side chains and the anthranilate carboxylate group of PRA. This is in agreement with experiments where introducing an arginine or removing the negative residue introduces TrpF activity in HisA systems.^46^

### Conformational Dynamics of Loop 1 in HisA and PriA and Its Impact on Selectivity

The importance of loops 5 and 6 of HisA and PriA for binding and catalysis is documented.^15,18,54^ In contrast, the precise catalytic role of loop 1 remains unclear, although substitutions at position 15 on this loop are important for facilitating the TrpF activity of this enzyme,^18,45,54^ and extending the conformation of loop 1 through duplication of residues 13–15 (HisA(dup13–15)) plays an important role in the acquisition of bifunctionality.^18,46,83^ In this context, *Se*HisA(dup13–15/D10G) is a bifunctional enzyme that can catalyze the isomerization of both ProFAR and PRA with modest catalytic efficiencies,^18^ and the corresponding crystal structure (PDB ID: 5AC7(18,55)) shows the enzyme in a “PRA-active” conformation with loops 1 and 6 in a closed state and loop 5 in an open state.

**Figure 7:**
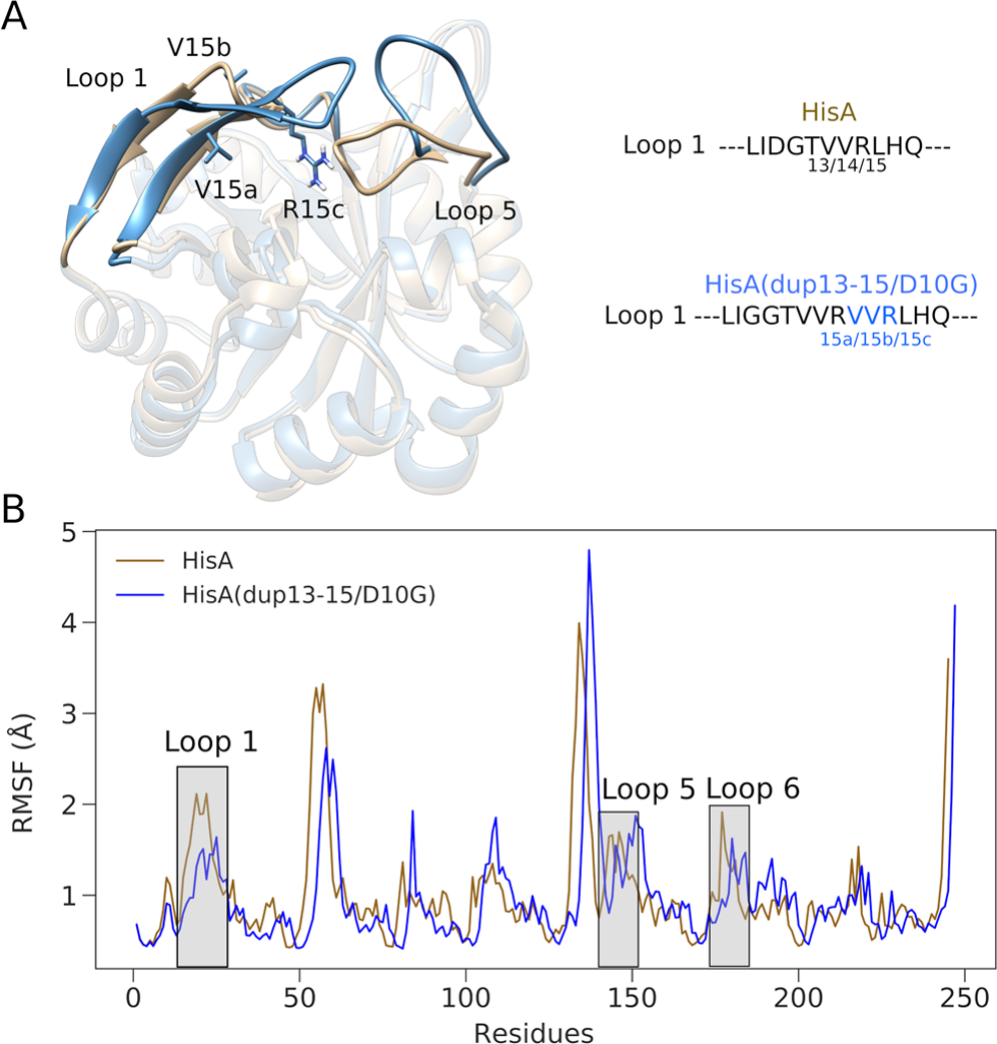
(A) Structural comparison of wild-type HisA (PDB ID: 5A5W(54,55)) and the *Se*HisA(dup13–15/D10G) variant (PDB ID: 5AC7(18,55)). The position of the key catalytic loops 1 and 5 is highlighted (loop 6 is not clearly visible as it is behind loops 1 and 5 but has the same conformation in both systems). A comparison between the loop 1 sequences in wild-type HisA and in the *Se*HisA(dup13–15/D10G) variant is provided as an inset. (B) Root-mean-square fluctuation (RMSF, Å) of all backbone C_α_ atoms in each enzyme.

When initiating simulations of the unliganded bifunctional *Se*HisA(dup13–15/D10G) variant starting from the loop 1 closed conformation, we did not observe any opening of loop 1. This contrasts with our simulations of the unliganded wild-type enzyme, where we sampled open conformations of this loop when we started from the closed conformation (PDB ID: 5A5W(54)) and removed ProFAR. This provides evidence, in addition to the discussion in ref (18), that the elongation of loop 1 heavily stabilizes this PRA-active conformation. This is further supported by examining the root-mean-square fluctuations (RMSF) of all C_α_ atoms in our simulations of these two enzymes starting from loop 1 in the closed conformation (Figure 7), where we observe that loop 1 is more flexible than either of loops 5 or 6 in simulations of the wild-type, but has reduced flexibility in simulations of the *Se*HisA(dup13–15/D10G) variant.

It is possible that product release could trigger a change from a closed to a wide-open conformation of loop 1, so we also performed steered molecular dynamics (sMD) simulations of the only crystal structure of a loop-elongated HisA variant with product bound to the active site (*Se*HisA(dup13–15/D10G/G102A/Q24L), PDB ID: 5AB3(18)). Specifically, we performed pulling simulations of products PRFAR and CdRP out of the enzyme from the bottom of the TIM-barrel. In doing so, we observed that the larger molecule, PRFAR, always induced a conformational change in loops 1 and 6 from a closed to an open and/or wide-open conformation upon pulling it out of the active site (Figure S8A,C, backbone RMSD of loop residues of up to ∼4.0 Å in loop 1 and ∼3.5 Å in loop 6, compared to the starting closed conformation). In contrast, CdRP is able to leave the active site without inducing large conformational changes in loop 1 (loop 1 opens in only one out of ten replicates), but pulling it out from the active site still induces a conformational change in loop 6 (Figure S8B,D, backbone RMSD of loop residues of up to ∼2.0 Å in loop 1 and ∼3.5 Å in loop 6, compared to the starting closed conformation). One interpretation consistent with this observation is that substantial loop rearrangement is required for efficient product release, and this in turn makes it possible that a slow (rate-limiting) product release step might be the reason for the low turnover numbers observed for catalyzing an intrinsically facile reaction^84^ (Table S2).

Interestingly, while the elongation of loop 1 through the duplication of residues 13–15 (VVR) appears to be essential for the change of specificity toward the isomerization of PRA (facilitated by the presence of a new stabilizing arginine side chain close to the active site, Figure S1),^18^ simply the loop duplication by itself is not enough to induce bifunctionality. That is, while the duplication elongates loop 1, it also rigidifies it such that *Se*HisA(dup13–15) does show some ability to isomerize PRA (*k*_cat_ > 0.15 s^–1^), but at the expense of losing all ability to isomerize ProFAR.^18^ Critical to bifunctionality is the inclusion of an additional substitution, D10G (*Se*HisA(dup13–15/D10G)).

**Figure 8:**
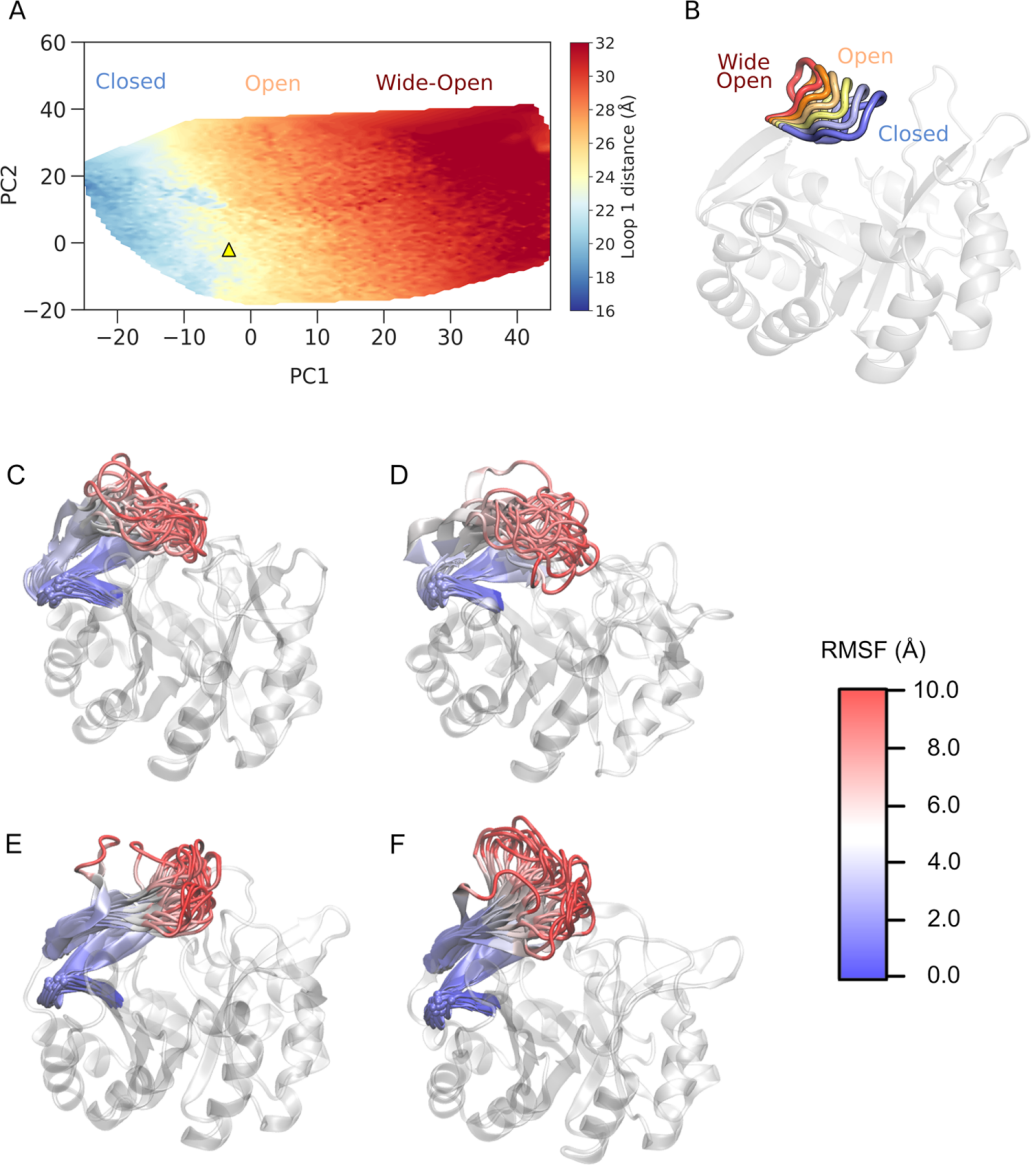
(A) Global projected loop 1 distance surfaces (Å) along the first two principal components (PC1 and PC2) obtained from applying Cartesian PCA to combined analysis of our conventional MD simulations of unliganded *Se*HisA(dup13–15) (PDB ID: 5G2I ^18^), *Se*HisA (dup13–15/D10G) (PDB ID: 5AC7(18,55)), *Se*HisA(dup13–15/D10G/G102A) (PDB ID: 5AC8 ^18^), and *Se*HisA(dup13–15/D10G/G102A/Q24L/V15[b]M) (PDB ID: 5G1Y ^18^). The starting *Se*HisA(dup13–15) loop 1 open state is indicated on this surface by a yellow triangle, ▲, and the same starting loop 1 conformation is used for simulations of all four systems. Note that this figure considers the motion of loop 1 along the PCs, as shown for PC1, projections along which include transitions from closed to wide-open states of loop 1 (B). PCA was performed on the mass-weighted Cartesian coordinates of the HisA variants compared to the coordinates of the corresponding open state, allowing us to explore the variation of the conformations of this loop in coordinate space. The loop 1 distance is defined as the center of mass of residues 15–25 of loop 1 and residue 129 from the barrel scaffold. Here, we considered distances <22.0 Å as corresponding to closed states (blue), between 23.0 and 28.0 Å as corresponding to open states (yellow), and >29.0 Å as corresponding to wide-open conformations (red), based on a combination of visual inspection and comparison of the closed-state crystal structures (PDB IDs: 5AB3(18,55) and 5AC7(18,55)), and using the *Se*HisA(dup13–15) variant as a reference. A 1 Å difference between states was applied to avoid fuzzy states between transition from closed to open state and from open to wide-open state. (C–F) Snapshots from our simulations, illustrating the conformational sampling of loop 1 in (C) wild-type *Se*HisA, (D) *Se*HisA(D10G), (E) *Se*HisA(dup13–15), and (F) *Se*HisA (dup13–15/D10G). The loops are colored mapped from red (most flexible) through white and to blue (least flexible), according to their calculated C_α_ RMSF.

In all HisA variants we studied, when performing conventional MD simulations starting from the closed conformation of loop 1, this loop is very stable and remains closed over our simulation timescales (Table S1). We therefore also initiated trajectories starting from the loop 1 open conformations of these variants to see whether the loop prefers to remain open, transition to wide-open conformations, or transition back to a closed conformation. PCA was then performed on the mass-weighted Cartesian coordinates of each enzyme compared to the coordinates of the corresponding loop 1, with closed, open, and wide-open conformations defined as described in the captions to Figures 8 and S9.

Inclusion of the D10G substitution (*Se*HisA(dup13–15/D10G)) increases the conformational space sampled by the elongated loop 1 (Figure 8) compared to *Se*HisA(dup13–15), allowing for the loop to take on wide-open configurations which in turn facilitate the entry and binding of ProFAR to the active site (Figure 8B, wide-open conformation). More specifically, as can be seen in Figure 8, the inclusion of the D10G substitution alone is enough to increase the conformational space sampled by loop 1 in *Se*HisA(D10G). The dup13–15 elongation rigidifies the whole loop compared to the wild type, and adding the D10G variant on the background of the elongation (*Se*HisA(dup13–15/D10G)) once again expands the conformational space sampled by the loop.

Our conventional MD simulations (500 ns per trajectory) are short, taking into account the slow turnover numbers of these enzymes (that suggest loop motions on the ms to s timescale).^18^ However, our observation that *Se*HisA(D10G) has an expanded conformational space sampled by loop 1, compared to *Se*HisA(dup13–15/D10G), agrees with prior NMR relaxation dispersion experiments.^18^ These detected μs to ms motions at 14 backbone ^15^N positions in the *Se*HisA(dup13–15/D10G) variant, compared to only three positions for the *Se*HisA(dup13–15) variant, and with two resonances that are unique to *Se*HisA(dup13–15/D10G). As a result, adding this substitution is sufficient to convert *Se*HisA(dup13–15/D10G) back to a bifunctional enzyme, through the exploitation of conformational dynamics, with *k*_cat_ of 0.09 s^–1^ for the isomerization of PRA, and 0.05 s^–1^ for the isomerization of ProFAR (Table S2).^18^

To explore this further, we examined simulations of four loop-elongated variants, specifically: dup13–15 (PDB ID: 5G2I(18)), dup13–15/D10G (PDB ID: 5AC7(18,55)), dup13–15/D10G/G102A (PDB ID: 5AC8(18)), and dup13–15/D10G/G102A/Q24L/V15[b]M (PDB ID: 5G1Y(18)). The first and last of these variants are only active toward the isomerization of PRA, whereas the middle two variants are bifunctional toward both PRA and ProFAR (Table S2).

Our simulations revealed how all of these enzymes are able to populate closed, open, and wide-open conformations (Table 1). However, the relative populations of these states depend strongly on enzyme variant: already, the D10G substitution by itself appears to be sufficient to cause a conformational shift toward a closed conformation compared to the distribution observed in the wild-type enzyme, and the population of the wide-open conformation is further reduced in the *Se*HisA(dup13–15) variant, consistent with the rigidification of loop 1. Introduction of the D10G and G102A substitutions reinstates sampling of the wide-open conformation and results in bifunctional variants that are also able to isomerize ProFAR. Finally, the *Se*HisA(dup13–15/D10G/Q102A/Q24L/V15[b]M) variant, which shows the highest TrpF activity of all variants studied in ref (18) (Table S2), also shows the most significant population shift toward sampling a closed conformation of loop 1.

**Table 1:** Relative Populations of the Closed, Open, and Wide-Open Conformations of Loop 1 Sampled in 10 × 500 ns MD Simulations of Wild-Type *Se*HisA and Variants^a^

system	function	closed	open	wide-open
wild-type	HisA	13	60	27
HisA(D10G)	HisA	33	49	18
HisA(dup13–15)	TrpF	35	59	6
HisA(dup13–15/D10G)	HisA + TrpF	29	53	18
HisA(dup13–15/D10G/G102A)	HisA + TrpF	13	65	22
HisA(dup13–15/D10G/G102A/Q24L/V15bM)	TrpF	64	31	5

a The relative populations (%) of the different loop 1 conformational states sampled during our simulations with HisA dup13–15 variants were determined by the number of frames with distances <22.0 Å defined as closed states, between 23.0 and 28.0 Å defined as open states, and >29.0 Å defined as wide-open conformations, as shown schematically in Figure 8, divided by the sum of all states considered for this analysis. A 1 Å difference between states was applied to avoid fuzzy states between transition from closed to open state and from open to wide-open state for the *Se*HisA(dup13–15) variants.

These observations are consistent with structural analyses^18^ indicating that the Q24L substitution is important because it introduces a new stabilizing interaction with V15b, as well as an even better interaction with V15[b]M such that the Q24L interaction is just as important as the VVR duplication for the adaptive benefit of the V15[b]M substitution to be realized. Both this variant and the *Se*HisA(dup13–15) variant, which show the lowest population of the hyper-open conformation, are TrpF specialists. We hypothesize that in the case of these variants, this is due to the presence of a Gly-Gly dyad in the hinge of loop 1, which provides the loop with enough flexibility to explore these wide-open conformations.

### Empirical Valence Bond Simulations of the Enzyme-Catalyzed Ribose Ring-Opening Step in the Isomerization of Substrates ProFAR and PRA

To further probe the role of loop 1 in HisA and PriA, we performed empirical valence bond (EVB)^69^ simulations of the initial ribose ring opening of substrates ProFAR and PRA (Figure 1), as catalyzed by wild-type and variant forms of the two enzymes. The calculated activation free energies for this step are not trivial to directly compare with the experimental turnover numbers; unfortunately, no kinetic data exist for the rates of the individual chemical steps. Furthermore, the observed *k*_cat_ values for these systems are extremely low—on the order of 1 s^–1^ (or lower) for both the HisA and TrpF reactions catalyzed by HisA and its variants.^18^ However, in the case of *Escherichia coli* TrpF, which has a higher turnover number than any of the enzymes studied here (*k*_cat_ of 30–40 s^–1^, see Table S2 for comparison), the rate-limiting step is an uncatalyzed keto-enol tautomerization that occurs after the ring-opening step (Figure 1).^85^

When also taking into account the potential involvement of loop dynamics in determining turnover rates, as seen in other enzymes with catalytically important conformational changes such as protein tyrosine phosphatases^16,19,22,82^ and indole-3-glycerol phosphate synthase,^86^ this means the experimental turnover numbers for the isomerization of ProFAR and PRA by the enzymes of interest do not correspond to a chemical step occurring in the enzyme active site. Furthermore, the Amadori reaction that occurs between the enzyme-catalyzed ring-opening reaction and the nonenzymatic tautomerization is likely to be fast (the uncatalyzed reaction occurs spontaneously at 25 °C).^84^ Thus, the ring-opening reaction is likely the slowest enzyme-catalyzed chemical step in the catalytic cycle, as supported by QM/MM studies of the HisA-catalyzed reaction.^83^ However, even if the experimentally measured turnover numbers (*k*_cat_) do not directly correspond to this step, they do produce a lower limit for the rate of this step (and thus an upper limit for the corresponding activation free energy for the rate-limiting step of the enzymatic reaction). Thus, comparing the calculated activation free energies for the ring opening in different variants, and in different conformational states of loop 1, can provide insight into the impact of loop dynamics and key amino acid substitutions on the rate of the slowest enzyme-catalyzed step.

**Figure 9:**
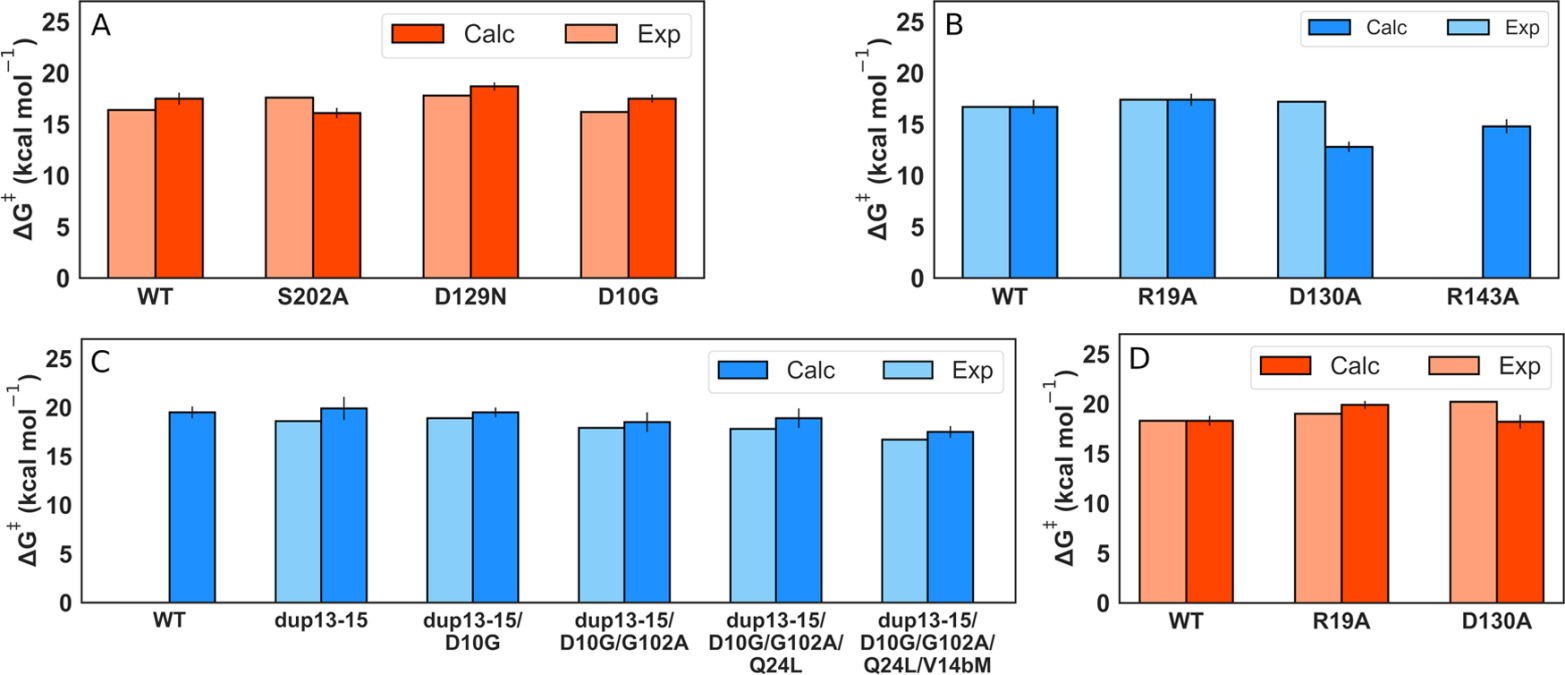
Calculated and experimentally derived activation energies for the isomerization of substrates (A, D) ProFAR (orange bars) and (B, C) PRA (blue bars), as catalyzed by (A, C) wild-type *Se*HisA and variants and (B, D) *Mt*PriA and variants. The calculated free energies (Δ*G*_calc_^‡^) correspond to the ribose ring-opening step (Tables S10 and S11). The experimental activation free energies (Δ*G*_exp_^‡^) were derived from the kinetic data presented in refs (15, 18).

**Figure 10:**
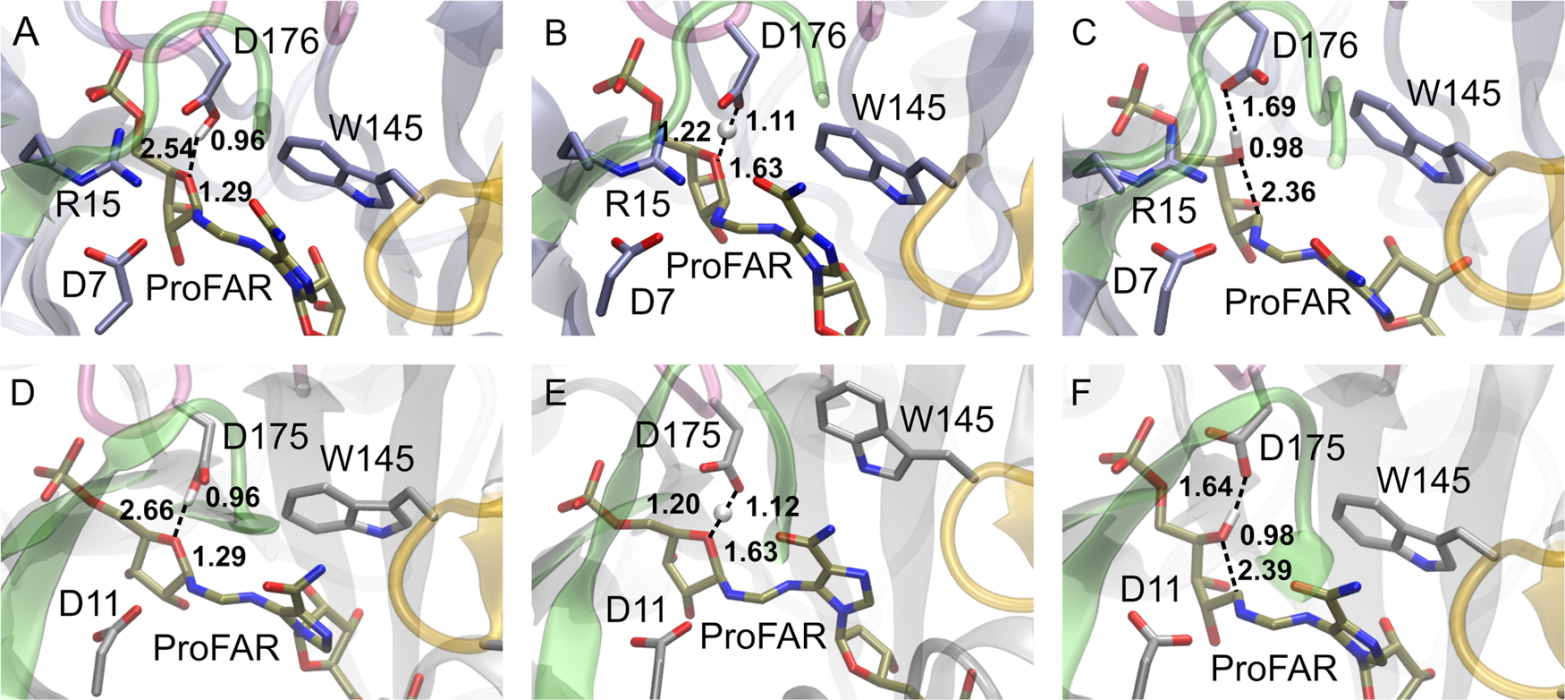
Representative structures of stationary points at the Michaelis complexes (MC), transition states (TS), and intermediate states (IS), respectively, for the ribose ring-opening step of the (A–C) HisA- and (D–F) PriA-catalyzed isomerization of ProFAR (by the wild-type enzymes). For EVB simulation details, see the Methodology section. Structures were selected based on clustering analysis using the hierarchical agglomerative algorithm, as implemented in CPPTRAJ.^77^ Note that the annotated catalytic distances are average values over 6000 snapshots extracted for each state from our EVB trajectories (from 30× individual 200 ps EVB mapping windows per stationary point/system). For a full list of reacting distances across all variants, see Tables S12 and S13.

Taking these limitations into account, the experimental and calculated activation energies are shown in Tables S10 and S11 and Figure 9, with representative Michaelis complexes (MC), transition states (TS), and ring-open intermediates for the ribose ring-opening reaction catalyzed by each wild-type enzyme shown in Figure 10 (ProFAR) and Figure S10 (PRA). We note that PRA has much more conformational freedom in the HisA and PriA active sites, which are both evolved to (also) accommodate the larger substrate ProFAR, and is thus capable of sampling a broad number of different conformations at the Michaelis complex, leading to an increased standard error of the mean in comparison to systems where ProFAR is bound to the active site of these enzymes. Curiously, our calculations reproduce the experimental trend in activation free energies derived from the turnover numbers relatively well for both substrates ProFAR (Table S10) and PRA (Table S11) for nearly all variants, despite the fact that observed *k*_cat_ does not correspond to a chemical step in the enzyme, as discussed above.

We used *Mt*PriA in its pro-ProFAR conformation as a reference state to calibrate our EVB simulations of the initial ring-opening of ProFAR, obtaining an activation free energy of 18.3 ± 0.5 kcal mol^–1^ for this reaction. The resulting EVB parameters were then used unchanged in all relevant systems, to obtain the data presented in Table S10. We obtained a slightly lower activation free energy of 17.5 ± 0.6 kcal mol^–1^ for the analogous reaction catalyzed by wild-type HisA, which is in agreement with the experimental observation that *Se*HisA is a better catalyst of ProFAR isomerization than *Mt*PriA (Table S10). To further validate our results, we performed single amino acid substitutions in each enzyme (R19A and D130A in PriA and S202A, D129N and D10G in HisA), and performed EVB simulations on these variants. In nearly all cases, we observed similar or higher activation free energies for the ring-opening step compared to the respective wild-type enzymes, in agreement with experimentally observed loss of activity upon these substitutions.^15,18^ We note, however, that this effect is less pronounced than the experiment in the case of the HisA(S202A) and PriA(D130A) variants, suggesting that the experimentally observed loss of activity is due to either a change in substrate positioning or loop conformation or dynamics, which we are not necessarily able to capture in our simulations when simply starting from the wild-type crystal structure and manually truncating these residues.

In the case of PRA (Table S11), we again used the reaction catalyzed by *Mt*PriA as our EVB reference state, this time with loop 5 in its pro-PRA conformation. While wild-type *Se*HisA does not show TrpF activity,^18^ we nevertheless modeled this reaction in *Se*HisA for comparison, starting from an idealized position of PRA in the active site (based on overlay with substrate ProFAR), and obtained an activation free energy of 19.5 ± 0.6 kcal mol^–1^. This is higher than the value obtained for ProFAR ring-opening by *Se*HisA (17.5 ± 0.6 kcal mol^–1^, Table S10), but is very similar to the value obtained for *Se*HisA(dup13–15) (19.9 ± 1.2 kcal mol^–1^, Table S11). This suggests that, in theory, *Se*HisA could catalyze PRA ring-opening if all loops are in the correct conformation and the substrate is optimally positioned, and that the lack of activity is not because of a high barrier to the chemical reaction catalyzed by this enzyme. All subsequent variants show a steady decrease in calculated activation free energies for the ring-opening reaction catalyzed from the loop closed conformation, in line with the increased population of the loop 1 closed conformation (Table 1) compensating for the lack of interactions between the substrate PRA and the stabilizing gripper loop 3 (Figure 3).

In the case of *Mt*PriA we modeled three individual substitutions (R19A, D130A, and R143A) and extended our EVB simulations (Table S11 and Figure 9), to specifically capture the impact of losing the electrostatic contribution of each truncated side chain on the activation free energy. In the case of *Mt*PriA(R19A), we obtained a slight increase in activation free energy that trends with the loss of activity observed experimentally.^15^ Structurally, this side chain is located on loop 1, and has been suggested to be important for the substrate-specific formation of the active site into pro-ProFAR and pro-PRA conformations.^15^ Our EVB calculations on both ProFAR and PRA ring-opening as catalyzed by *Mt*PRA(R19A) (Tables S10 and S11) suggest an important electrostatic role for this residue. Similarly, kinetic and structural data suggest that truncation of R143 and D130 to alanine negatively impacts PRA isomerization (in the case of R143A, *k*_cat_/*K*_M_ is reduced from 1.7 × 10^5^ to 6.0 × 10^3^ M^–1^ s^–1^ on the introduction of this substitution).^15^ In contrast, we obtain lower activation free energies for PRA ring-opening catalyzed by both the D130A and R143A variants. However, especially in the case of the R143A variant, it is unclear if the experimentally observed effect is expressed on *k*_cat_, *K*_M_, or both, and it is plausible that the loss of activity is due to structural effects that prohibit productive substrate binding, and therefore are not captured in our simulations. This scenario would be similar to our observations from an analogous system activated by a ligand-gated conformational change, glycerol-3-phosphate dehydrogenase (GPDH).^38^

Finally, to examine how important loop 1 closure is for PRA isomerization, we performed EVB simulations of ProFAR and PRA ring-opening as catalyzed by *Se*HisA (both wild-type and the *Se*HisA(dup13–15/D10G/G102A/Q24L/V15[b]M variant)) and *Mt*PriA (wild-type) with loop 1 in the open conformation (Tables S10 and S11). Due to the lack of crystal structures, we extracted structures from our conventional MD simulations as starting points for these EVB simulations. For PRA isomerization, we obtained much higher activation free energies for ring-opening when loop 1 was modeled in an open conformation than a closed conformation, due to a combination of the loss of key interactions between loop 1 and the substrate, and also extra solvent exposure of the active site when this loop opens. However, we observed no impact on the activation free energy when modeling ProFAR isomerization starting with loop 1 open. Therefore, while the catalytic importance of loops 5 and 6 is well established,^15,18,54^ our EVB calculations clearly show that, in addition, full closure of loop 1 into a catalytically competent conformation is essential for efficient PRA isomerization.

## Overview and Conclusions

In the present work, we combined conventional and enhanced sampling MD simulations, with EVB calculations, to explore the role of loop dynamics in dictating the selectivity and evolvability of the evolutionarily important model enzymes, HisA, TrpF, and PriA.^18,45−48,87^ The roles of loop dynamics and ligand-gated conformational changes in TIM-barrel proteins and proteins from other folds have been a topic of substantial research interest (e.g., refs (8, 34−38, 88−97)). However, what makes the current enzymes stand out from these prior studies is the importance of not one but two (TrpF) or even three (HisA and PriA) long, mobile loops (Figure 1), the specific conformations of which have been suggested to play an important role in facilitating the substrate selectivity of PriA and evolved HisA variants.^15,18,54^ Thus, these enzymes undergo particularly complex loop dynamics. In addition, prior enzymes that have been characterized as being activated by ligand-gated conformational changes, such as TPI and OMPDC, are extremely proficient.^34−38,98−100^ In contrast, the enzymes studied here are relatively inefficient (Table S2),^15,18,79^ with turnover numbers of ∼10 s^–1^ or less,^18,52,101^ despite catalyzing an intrinsically facile reaction.^84^ Furthermore, while loop motions in TIM-barrel enzymes such as TPI are relatively fast (on the μs timescale),^96^ motions on the ms timescale have been detected in the evolved HisA variants,^18^ and thus loop motions are likely to be (at least partially) rate-limiting in these enzymes.

At the simplest level, our simulations agree with structural data^15^ that TrpF, PriA, and HisA have increasingly large (in terms of active site volume) and “breathable” active sites (Table S8), allowing for the accommodation of ProFAR by HisA and PriA but not PRA-specific TrpF, the active site of which is too small to accommodate the larger substrate.^15^ More importantly, HisA and PriA both possess “gripper residues” interacting with the nonreactive phosphodianion group of ProFAR (R83 and S103 in HisA; R85 and T105 in PriA, Figure 2), that are very similar to analogous interactions in other enzymes activated by ligand-gated conformational changes, such as TPI.^34,35^ Of note, however, is that the HisA/PriA gripper residues are contributed from the less mobile loops 3 and 4, unlike the primary gripper loop in TPI (loop 6), which undergoes a substantial conformational change upon ligand binding.^12^ Other TIM-barrel proteins such as OMPDC possess analogous gripper loops to TPI loop 6,^12^ showing evidence for convergent evolution on these different (βα)_8_-barrel scaffolds.

Our simulations show that while the gripper interaction is stable in HisA throughout the simulations, in PriA, there is electrostatic repulsion between R85 and an additional active site arginine, R143, which causes instabilities in the catalytic loops (in particular loop 5, Figure S3), as well as in the substrate positioning in the active site such that ProFAR is less stable in the PriA active site than in the HisA active site. This is in effect a ligand-gated effect, where interaction with the nonreactive phosphodianion (which is not present in the smaller substrate, PRA) facilitates the stability of catalytically important loop 5. Thus, the underlying principles driving loop stability are similar to those of other enzymes that are activated by ligand-gated changes.

PCA on our simulations showed that the active site loops in these enzymes can sample a range of wide-open conformations with transitions between them, with their conformational flexibility being stabilized by ligand binding (although less so in the bifunctional *Mt*PriA than the ProFAR-specific *Se*HisA). In contrast, the active site loops in *Tm*TrpF, which binds a smaller substrate and lacks the gripper, remain dynamic. In particular, loop 6 (Figure 8) samples a range of open conformations even with PRA bound to the active site. Additionally, all HisA variants from the real-time evolution experiment^46^ studied here also sample a range of open and wide-open conformations. In this context, however, the D10G substitution on loop 1 appears to be sufficient by itself to increase the population of the closed conformation sampled during our simulations (Table 1), and the highest proportion of closed conformation is observed in simulations of the *Se*HisA(dup13–15/D10G/Q102A/Q24L/V15[b]M) variant, which has the highest TrpF activity^18^ (Table S2).

Pulling simulations, where we pulled products PRFAR and CdRP out of the *Se*HisA(dup13–15/D10G/G102A/Q24L) active site, showed significant conformational changes in both loops 1 and 6 when pulling PRFAR out, whereas CdRP was pulled out with loop 1 remaining stable and loop 6 opening. This suggests that loop 1 dynamics are more important for binding of ProFAR and subsequent release of PRFAR, than for the smaller substrate PRA and its product CdRP, whereas loop 1 dynamics appears to be critical to catalysis (Table S11). In addition, as mentioned above, these enzymes are relatively inefficient (Table S2).^18,52,101^ When taking this into account, the substantial rearrangements that we observe for both compounds suggests that a slow (potentially rate-limiting) product release step that requires substantial loop rearrangement may be the reason for the low turnover numbers observed for an otherwise facile reaction, further providing evidence that turnover rates are being regulated by loop dynamics.

In contrast to HisA, which undergoes conformational changes of loop 1 during the real-time evolution experiment that changes its selectivity from ProFAR-specific to PRA-specific,^18,46^ the bifunctional PriA is already able to rearrange its active site in its wild-type form, to accommodate the different substrates through alternation between pro-ProFAR and pro-PRA conformations of loop 5.^15^ These conformational changes both reduce repulsion between the two active site arginine side chains that in turn destabilize loop 5 dynamics (Figure S3), as well as disrupting the stacking interaction between the W145 side chain and the substrate ProFAR (Figure S4). This rationalizes the preference of this enzyme toward PRA rather than ProFAR, despite its similarities with the HisA active site. We also note that loss of the stacking interaction between W145 and ProFAR was one aspect of the gain of PRA isomerization activity in the *Se*HisA(dup13–15) variant from the real-time evolution experiment.^18,46^

Finally, we performed EVB simulations of the first ring-opening step of ProFAR and (where relevant) PRA isomerization (Figure 1) by wild-type HisA, PriA, and variants. As described above, the actual rate of the chemical step in these enzymes is unknown, since the rate-limiting step in, at least tryptophan biosynthesis, appears to be an uncatalyzed keto-enol tautomerization step.^85^ Nevertheless, we saw that our calculated activation free energies for the ring-opening reaction trend well with the differences in activation free energies derived using the measured turnover numbers as an upper limit for this value, suggesting there is both a chemical and a dynamical component to the observed changes in activity upon substitution and/or duplication of key residues. Furthermore, to reproduce the relevant PriA activation free energies, it was necessary to start from different structures of loop 5, following earlier structural analysis that demonstrated the loop can exist in either a knot-like pro-PRA or β-hairpin pro-ProFAR conformation (Figure S1), depending on which substrate is bound.^15^ Clearly, the ease with which this rearrangement can occur will also impact the selectivity of this enzyme. Finally, EVB simulations of wild-type *Se*HisA, *Mt*PriA and the *Se*HisA(dup13–15/D10G/G102A/Q24L/V15[b]M) variant with loop 1 in an open conformation all yielded substantially higher energies for PRA isomerization, whereas ProFAR isomerization (by wild-type HisA) seems to be unaffected (Tables S10 and S11). This further emphasizes the importance of the correct closure of loop 1 for isomerization of the smaller substrate PRA. This is in contrast to substrate binding, where the conformational plasticity of loop 1 appears to be far more important for facilitating the correct binding of ProFAR than PRA (Figure S8).

Taken together, these observations highlight the critical role of multiple decorating loops of HisA, PriA, and TrpF in facilitating catalysis. These enzymes stand out from prior systems that have been demonstrated to be activated by ligand-gated conformational changes^34−41^ due to a number of factors. First, we have shown the interdependent motion of three long loops (or two in TrpF), none of which dominates and each of which is capable of undergoing substantial conformational changes to facilitate the turnover of different substrates. Second, unlike prior systems which show substantial rate accelerations compared to the uncatalyzed reactions with comparatively rapid loop motions, in these enzymes, the catalyzed reaction is already intrinsically fast,^84^ whereas loop motion is slow and appears to be controlling the reaction rate.

It has been argued that a PriA-like gene product could have been the common evolutionary ancestor for both HisA and TrpF.^53,87,102^ Ancestral sequence reconstruction has also been used to suggest that ancient HisA precursors were likely bifunctional and that this bifunctionality persisted over at least a 2 billion year time span.^52^ However, as shown in Figure 5, HisA and PriA exploit loops 1, 5, and 6 to facilitate activity, whereas TrpF lacks an analogue for loop 1, and isomerizes PRA harnessing just two catalytic loops 3 and 6. Our results suggest that an evolutionary trajectory from a PriA-like ancestor to an extant TrpF would be surprisingly complex. Loop 1 would need to be truncated (and not extended, as when *Se*HisA was artificially evolved into a TrpF),^18^ and the interdependency of this third loop would need to be lost, raising the question of what evolutionary path would take a PriA-like precursor to TrpF, while completely abolishing this loop. We note that the natural or evolved bifunctionality in these enzymes being driven by the ability to adapt different conformational states is consistent with prior studies on the laboratory evolved bifunctionality of a phosphotriesterase from *Pseudomonas diminuta*,^30,103^ suggesting that it may be a more common phenomenon not limited to these enzymes.

Despite the novel aspects of the systems studied here, a key similarity with prior systems is the generalizability of ligand-gated conformational changes across a wide range of systems, in particular TIM-barrel proteins,^34^ which tend to possess flexible loops decorating their active sites. The conservation of such ligand-gated conformational changes—albeit triggered in different loops—suggests that these loops evolve independently of the barrel providing a starting point for the emergence and divergence of new enzyme activities.^21,47^ In addition, TrpF, for example, has been shown to be highly tolerant of variations in loop 6 sequence such that grafting sequences from related enzymes such as TrpA, HisA, and PriA onto the TrpF scaffold did not abolish activity.^104^ This is significant considering the high evolvability of this scaffold,^8^ and the wide range of chemistry it supports,^47^ which makes it very desirable as a starting point for protein engineering efforts. In addition, it could be argued that the real-time evolution experiment that bestowed PRA isomerization activity to HisA^46^ effectively performed “natural” loop-engineering by altering the conformations of key active site loops.^18^ Our work therefore suggests that, more broadly, loop grafting and engineering is a powerful tool for generating novel enzymes with tailored activities and specificities, even in complex systems with multiple highly mobile and interdependent catalytic loops.

## Supporting Information Available

Computational methodology, additional structural, and simulation analysis; representative EVB input files, starting structures, parameter files, and *Q*6 topology files have been uploaded to Zenodo, DOI: 10.5281/zenodo.5893598 (PDF)

## References

[ref1] Gora A.; Brezovsky J.; Damborsky J. Gates of Enzymes. Chem. Rev. 2013, 113, 5871–5923. 10.1021/cr300384w. 23617803 PMC3744840

[ref2] McGeagh J. D.; Ranaghan K. E.; Mulholland A. J. Protein Dynamics and Enzyme Catalysis: Insights from Simulations. Biochim. Biophys. Acta, Proteins Proteomics 2011, 1814, 1077–1092. 10.1016/j.bbapap.2010.12.002. 21167324

[ref3] Villali J.; Kern D. Choreographing an Enzyme’s Dance. Curr. Opin. Chem. Biol. 2010, 14, 636–643. 10.1016/j.cbpa.2010.08.007. 20822946 PMC2987738

[ref4] Tawfik O. K. A. D. S.; Tawfik D. S. Enzyme Promiscuity: A Mechanistic and Evolutionary Perspective. Annu. Rev. Biochem. 2010, 79, 471–505. 10.1146/annurev-biochem-030409-143718. 20235827

[ref5] Doshi U.; Hamelberg D. The Dilemma of Conformational Dynamics in Enzyme Catalysis: Perspectives from Theory and Experiment. Adv. Exp. Med. Biol. 2014, 805, 221–243. 10.1007/978-3-319-02970-2_10. 24446364

[ref6] Kohen A. Role of Dynamics in Enzyme Catalysis: Substantial versus Semantic Controversies. Acc. Chem. Res. 2015, 48, 466–473. 10.1021/ar500322s. 25539442 PMC4334245

[ref7] Klinman J. P. Dynamically Achieved Active Site Precision in Enzyme Catalysis. Acc. Chem. Res. 2015, 48, 449–456. 10.1021/ar5003347. 25539048 PMC4334267

[ref8] Wierenga R. K. The TIM-Barrel Fold: A Versatile Framework for Efficient Enzymes. FEBS Lett. 2001, 492, 193–198. 10.1016/S0014-5793(01)02236-0. 11257493

[ref9] Rajagopalan P. T. R.; Benkovic S. J. Preorganization and Protein Dynamics in Enzyme Catalysis. Chem. Rec. 2002, 2, 24–36. 10.1002/tcr.10009. 11933259

[ref10] McElheny D.; Schnell J. R.; Lansing J. C.; Dyson H. J.; Wright P. E. Defining the Role of Active-Site Loop Fluctuations in Dihydrofolate Reductase Catalysis. Proc. Natl. Acad. Sci. U.S.A. 2005, 102, 5032–5037. 10.1073/pnas.0500699102. 15795383 PMC556001

[ref11] Tawfik D. S. Loop Grafting and the Origins of Enzyme Species. Science 2006, 311, 475–476. 10.1126/science.1123883. 16439649

[ref12] Malabanan M. M.; Amyes T. L.; Richard J. P. A Role for Flexible Loops in Enzyme Catalysis. Curr. Opin. Struct. Biol. 2010, 20, 702–710. 10.1016/j.sbi.2010.09.005. 20951028 PMC2994964

[ref13] Wierenga R. K.; et al. Triosephosphate Isomerase: A Highly Evolved Biocatalyst. Cell. Mol. Life Sci. 2010, 67, 3961–3982. 10.1007/s00018-010-0473-9. 20694739 PMC11115733

[ref14] Bhabha G.; Lee J.; Ekiert D. C.; Gam J.; Wilson I. A.; Dyson H. J.; Benkovic S. J.; Wright P. E. A Dynamic Knockout Reveals That Conformational Fluctuations Influence the Chemical Step of Enzyme Catalysis. Science 2011, 332, 234–238. 10.1126/science.1198542. 21474759 PMC3151171

[ref15] Due A. V.; Kuper J.; Geerlof A.; von Kries J. P.; Wilmanns M. Bisubstrate Specificity in Histidine/Tryptophan Biosynthesis Isomerase from *Mycobacterium tuberculosis* by Active Site Metamorphosis. Proc. Natl. Acad. Sci. U.S.A. 2011, 108, 3554–3559. 10.1073/pnas.1015996108. 21321225 PMC3048130

[ref16] Whittier S. K.; Hengge A. C.; Loria J. P. Conformational Motions Regulate Phosphoryl Transfer in Related Protein Tyrosine Phosphatases. Science 2013, 341, 899–903. 10.1126/science.1241735. 23970698 PMC4078984

[ref17] Blaha-Nelson D.; Krüger D. M.; Szeler K.; Ben-David M.; Kamerlin S. C. L. Active Site Hydrophobicity and the Convergent Evolution of Paraoxonase Activity in Structurally Divergent Enzymes: The Case of Serum Paraoxonase 1. J. Am. Chem. Soc. 2017, 139, 1155–1167. 10.1021/jacs.6b10801. 28026940 PMC5269640

[ref18] Newton M. S.; Guo X.; Söderholm A.; Näsvall J.; Lundström P.; Andersson D. I.; Selmer M.; Patrick W. M. Structural and Functional Innovations in the Real-Time Evolution of New (βα)_8_ Barrel Enzymes. Proc. Natl. Acad. Sci. U.S.A. 2017, 114, 4727–4732. 10.1073/pnas.1618552114. 28416687 PMC5422803

[ref19] Moise G.; Morales Y.; Beaumont V.; Caradonna T.; Loria J. P.; Johnson S. J.; Hengge A. C. A YopH PTP1B Chimera Shows the Importance of the WPD-Loop Sequence to the Activity, Structure, and Dynamics of Protein Tyrosine Phosphatases. Biochemistry 2018, 57, 5315–5326. 10.1021/acs.biochem.8b00663. 30110154

[ref20] Moreira C.; Calixto A. R.; Richard J. P.; Kamerlin S. C. L. The Role of Ligand-Gated Conformational Changes in Enzyme Catalysis. Biochem. Soc. Trans. 2019, 47, 1449–1460. 10.1042/BST20190298. 31657438 PMC6824834

[ref21] Richard J. P. Protein Flexibility and Stiffness Enable Efficient Enzymatic Catalysis. J. Am. Chem. Soc. 2019, 141, 3320–3331. 10.1021/jacs.8b10836. 30703322 PMC6396832

[ref22] Crean R. M.; Biler M.; van der Kamp M. W.; Hengge A. C.; Kamerlin S. C. L. Loop Dynamics and Enzyme Catalysis in Protein Tyrosine Phosphatases. J. Am. Chem. Soc. 2021, 143, 3830–3845. 10.1021/jacs.0c11806. 33661624 PMC8031367

[ref23] James L. C.; Tawfik D. S. Conformational Diversity and Protein Evolution—A 60 Year Old Hypothesis Revisited. Trends Biochem. Sci. 2003, 28, P361–P368. 10.1016/S0968-0004(03)00135-X. 12878003

[ref24] Tokuriki N.; Tawfik D. S. Protein Dynamism and Evolvability. Science 2009, 324, 203–207. 10.1126/science.1169375. 19359577

[ref25] Petrović D.; Risso V. A.; Kamerlin S. C. L.; Sanchez-Ruiz J. M. Conformational Dynamics and Enzyme Evolution. J. R. Soc., Interface 2018, 15, 20180330 10.1098/rsif.2018.0330. 30021929 PMC6073641

[ref26] Johansson K. E.; Lindorff-Larsen K. Structural Heterogeneity in Protein Evolution and Design. Curr. Opin. Struct. Biol. 2018, 48, 157–163. 10.1016/j.sbi.2018.01.010. 29413956

[ref27] Maria-Solano M. A.; Serrano-Hervás E.; Romero-Rivera A.; Iglesias-Fernández J.; Osuna S. Role of Conformational Dynamics in the Evolution of Novel Enzyme Functions. Chem. Commun. 2018, 54, 6622–6634. 10.1039/C8CC02426J. PMC6009289 29780987

[ref28] Campitelli P.; Modi T.; Kumar S.; Ozkan S. B. The Role of Conformational Dynamics and Allostery in Modulating Protein Evolution. Annu. Rev. Biochem. 2020, 49, 267–288. 10.1146/annurev-biophys-052118-115517. 32075411

[ref29] Damry A. M.; Jackson C. J. The Evolution and Engineering of Enzyme Activity Through Tuning Conformational Landscapes. Protein Eng., Des. Sel. 2021, 34, gzab009 10.1093/protein/gzab009. 33903911

[ref30] Campbell E. C.; Correy G. J.; Mabbitt P. D.; Buckle A. M.; Tokuriki N.; Jackson C. J. Laboratory Evolution of Protein Conformational Dynamics. Curr. Opin. Struct. Biol. 2018, 50, 49–57. 10.1016/j.sbi.2017.09.005. 29120734

[ref31] Crean R. M.; Gardner J. M.; Kamerlin S. C. L. Harnessing Conformational Plasticity to Generate Designer Enzymes. J. Am. Chem. Soc. 2020, 142, 11324–11342. 10.1021/jacs.0c04924. 32496764 PMC7467679

[ref32] Osuna S. The Challenge of Predicting Distal Active Site Mutations in Computational Enzyme Design. Wiley Interdiscip. Rev.: Comput. Mol. Sci. 2020, 11, e1502 10.1002/wcms.1502.

[ref33] Nestl B. M.; Hauer B. Engineering of Flexible Loops in Enzymes. ACS Catal. 2014, 4, 3201–3211. 10.1021/cs500325p.

[ref34] Richard J. P.; Zhai X.; Malabanan M. M. Reflections on the Catalytic Power of a TIM-barrel. Bioorg. Chem. 2014, 57, 206–212. 10.1016/j.bioorg.2014.07.001. 25092608 PMC4256097

[ref35] Richard J. P. A Paradigm for Enzyme-Catalyzed Proton Transfer at Carbon: Triosephosphate Isomerase. Biochemistry 2012, 51, 2652–2661. 10.1021/bi300195b. 22409228 PMC3319633

[ref36] Richard J. P.; Amyes T. L.; Reyes A. C. Orotidine 5′-Monophosphate Decarboxylase: Probing the Limits of the Possible for Enzyme Catalysis. Acc. Chem. Res. 2018, 51, 960–969. 10.1021/acs.accounts.8b00059. 29595949 PMC6016548

[ref37] He R.; Reyes A. C.; Amyes T. L.; Richard J. P. Enzyme Architecture: The Role of a Flexible Loop in Activation of Glycerol-3-Phosphate Dehydrogenase for Catalysis of Hydride Transfer. Biochemistry 2018, 57, 3227–3236. 10.1021/acs.biochem.7b01282. 29337541 PMC6001809

[ref38] Mhashal A. R.; Romero-Rivera A.; Mydy L. S.; Cristobal J. R.; Gulick A. M.; Richard J. P.; Kamerlin S. C. L. Modeling the Role of a Flexible Loop and Active Site Side Chains in Hydride Transfer Catalyzed by Glycerol-3-phosphate Dehydrogenase. ACS Catal. 2020, 10, 11253–11267. 10.1021/acscatal.0c02757. 33042609 PMC7536716

[ref39] Kholodar S. A.; Murkin A. S. DXP Reductoisomerase: Reaction of the Substrate in Pieces Reveals a Catalytic Role for the Non-Reacting Phosphodianion Group. Biochemistry 2013, 52, 2302–2308. 10.1021/bi400092n. 23473304

[ref40] Kholodar S. A.; Allen C. L.; Gulick A. M.; Murkin A. S. The Role of Phosphate in a Multistep Enzymatic Reaction: Reactions of the Substrate and Intermediate in Pieces. J. Am. Chem. Soc. 2015, 137, 2748–2756. 10.1021/ja512911f. 25642788 PMC4507815

[ref41] Ray W. J.; Long J. W.; Owens J. D. An Analysis of the Substrate-Induced Rate Effect in the Phosphoglucomutase System. Biochemistry 1976, 15, 4006–4017. 10.1021/bi00663a015. 963019

[ref42] Kadumuri R. V.; Vadrevu R. Diversity in αβ and βα Loop Connections in TIM Barrel Proteins: Implications for Stability and Design of the Fold. Interdiscip. Sci.: Comput. Life Sci. 2018, 10, 805–812. 10.1007/s12539-017-0250-7. 29064074

[ref43] Wierenga R. The TIM-Barrel Fold: A Versatile Framework for Efficient Enzymes. FEBS Lett. 2001, 492, 193–198. 10.1016/S0014-5793(01)02236-0. 11257493

[ref44] Liao Q.; Kulkarni Y.; Sengupta U.; Petrović D.; Mulholland A. J.; van der Kamp M. W.; Strodel B.; Kamerlin S. C. L. Loop Motion in Triosephosphate Isomerase is not a Simple Open and Shut Case. J. Am. Chem. Soc. 2018, 140, 15889–15903. 10.1021/jacs.8b09378. 30362343

[ref45] Lundin E.; Näsvall J.; Andersson D. I. Mutational Pathways and Trade-Offs Between HisA and TrpF Functions: Implications for Evolution via Gene Duplication and Divergence. Front. Microbiol. 2020, 11, 588235 10.3389/fmicb.2020.588235. 33154742 PMC7591586

[ref46] Näsvall J.; Sun L.; Roth J. R.; Andersson D. I. Real-Time Evolution of New Genes by Innovation, Amplification and Divergence. Science 2012, 338, 384–387. 10.1126/science.1226521. 23087246 PMC4392837

[ref47] Romero-Romero S.; Kordes S.; Michel F.; Höcker B. Evolution, Folding, and Design of TIM Barrels and Related Proteins. Curr. Opin. Struct. Biol. 2021, 68, 94–104. 10.1016/j.sbi.2020.12.007. 33453500 PMC8250049

[ref48] Copley S. D. Evolution of New Enzymes By Gene Duplication and Divergence. FEBS J. 2020, 287, 1262–1283. 10.1111/febs.15299. 32250558 PMC9306413

[ref49] Henn-Sax M.; Thoma R.; Schmidt S.; Hennig M.; Kirschner K.; Sterner R. Two (βα)_8_-Barrel Enzymes of Histidine and Tryptophan Biosynthesis Have Similar Reaction Mechanisms and Common Strategies for Protecting Their Labile Substrates. Biochemistry 2002, 41, 12032–12042. 10.1021/bi026092h. 12356303

[ref50] Juárez-Vázquez A. L.; Edirisinghe J. N.; Verduzco-Castro E. A.; Michalska K.; Wu C.; Noda-Garcia L.; Endres M.; Medina-Ruíz S.; Santoyo-Flores J.; Carrillo-Tripp M.; Ton-That H.; Joachimiak A.; Henry C. S.; Barona-Gómez F. Evolution of Substrate Specificity in a Retained Enzyme Driven by Gene Loss. eLife 2017, 6, e22679 10.7554/eLife.22679. 28362260 PMC5404923

[ref51] Kuper J.; Dönges C.; Wilmanns M. Two-fold Repeated (βα)_4_ Half-Barrels May Provide a Molecular Tool for Dual Substrate Specificity. EMBO Rep. 2005, 6, 134–139. 10.1038/sj.embor.7400330. 15654319 PMC1299240

[ref52] Plach M. G.; Reisinger B.; Sterner R.; Merkl R. Long-Term Persistence of Bi-functionality Contributes to the Robustness of Microbial Life to Exaptation. PLoS Genet. 2016, 12, e1005836 10.1371/journal.pgen.1005836. 26824644 PMC4732765

[ref53] Jürgens C.; Strom A.; Wegener D.; Hettwer S.; Wilmans S.; Sterner R. Directed Evolution of a (βα)_8_-Barrel Enzyme to Catalyze Related Reactions in Two Different Metabolic Pathways. Proc. Natl. Acad. Sci. U.S.A. 2000, 97, 9925–9930. 10.1073/pnas.160255397. 10944186 PMC27628

[ref54] Söderholm A.; Guo X.; Newton M. S.; Evans G. B.; Näsvall J.; Patrick W. M.; Selmer M. Two-step Ligand Binding in a (βα)_8_ Barrel Enzyme: Substrate-Bound Structures Shed New Light on the Catalytic Cycle of HisA. J. Biol. Chem. 2015, 290, 24657–24668. 10.1074/jbc.M115.678086. 26294764 PMC4598979

[ref55] Berman H. M.; Westbrook J.; Feng Z.; Gililand G.; Bhat T. N.; Weissig H.; Shindyalov I. N.; Bourne P. E. The Protein Data Bank. Nucleic Acids Res. 2000, 28, 235–242. 10.1093/nar/28.1.235. 10592235 PMC102472

[ref56] Katebi A. R.; Jernigan R. L. The Critical Role of the Loops of Triosephosphate Isomerase for its Oligomerization, Dynamics and Functionality. Protein Sci. 2014, 23, 213–228. 10.1002/pro.2407. 24318986 PMC3926746

[ref57] Shapovalov M. X.; Dunbrack R. L. Jr. A Smoothed Backbone-Dependent Rotamer Library for Proteins Derived from Adaptive Kernel Density Estimates and Regressions. Structure 2011, 19, 844–858. 10.1016/j.str.2011.03.019. 21645855 PMC3118414

[ref58] Pettersen E. F.; Goddard T. D.; Huang C. C.; Couch G. S.; Greenblatt D. M.; Meng E. C.; Ferrin T. E. UCSF Chimera—A Visualization System for Exploratory Research and Analysis. J. Comput. Chem. 2004, 25, 1605–1612. 10.1002/jcc.20084. 15264254

[ref59] Šali A.; Blundell T. L. Comparative Protein Modelling by Satisfaction of Spatial Restraints. J. Mol. Biol. 1993, 234, 779–815. 10.1006/jmbi.1993.1626. 8254673

[ref60] Olsson M. H. M.; Søndergaard C. R.; Rostkowski M.; Jensen J. H. PROPKA3: Consistent Treatment of Internal and Surface Residues in Empirical p*K*_a_ Predictions. J. Chem. Theory Comput. 2011, 7, 525–537. 10.1021/ct100578z. 26596171

[ref61] Wang J.; Wang W.; Kollman P. A.; Case D. A. Automatic Atom Type and Bond Type Perception in Molecular Mechanical Calculations. J. Mol. Graphics Modell. 2006, 25, 247–260. 10.1016/j.jmgm.2005.12.005. 16458552

[ref62] Frisch M. J.; Trucks G. W.; Schlegel H. B.; Scuseria G. E.; Robb M. A.; Cheeseman J. R.; Scalmani G.; Barone V.; Petersson G. A.; Nakatsuji H.; Li X.; Caricato M.; Marenich A.; Bloino J.; Janesko B. G.; Gomperts R.; Mennucci B.; Hratchian H. P.; Ortiz J. V.; Izmaylov A. F.; Sonnenberg J. L.; Williams-Young D.; Ding F.; Lipparini F.; Egidi F.; Goings J.; Peng B.; Petrone A.; Henderson T.; Ranansinghe D.; Zakrzewski V. G.; Gao J.; Rega N.; Zheng G.; Liang W.; Hada M.; Ehara M.; Toyota K.; Fukuda R.; Hasegawa J.; Ishida M.; Nakajima T.; Honda Y.; Kitao O.; Nakai H.; Vreven T.; Throssell K.; Montgomery Jr J. A.; Peralta J. E.; Ogliaro F.; Bearpark M.; Heyd J. J.; Brothers E.; Kudin K. N.; Staroverov V. N.; Keith T.; Kobayashi R.; Normand J.; Raghavachari K.; Rendell A.; Burant J. C.; Iyengar S. S.; Tomasi J.; Cossi M.; Millam J. M.; Klene M.; Adamo C.; Cammi R.; Ochterski J. W.; Martin R. L.; Morokuma K.; Farkas O.; Foresman J. B.; Fox D. J. Gaussian 09, revision E.01; Gaussian, Inc.: Wallingford, CT, 2016.

[ref63] Wang J.; Wolf R. M.; Caldwell J. W.; Kollmann P. A.; Case D. A. Development and Testing of a General AMBER Force Field. J. Comput. Chem. 2004, 25, 1157–1174. 10.1002/jcc.20035. 15116359

[ref64] Case D. A.; Betz R. M.; Cerutti D. S.; Cheatham T. E. III; Darden T. A.; Duke R. E.; Giese T. J.; Gohlke H.; Goetz A. W.; Homeyer N.; Izadi S.; Janowski P.; Kaus J.; Kovalenko A.; Lee T. S.; LeGrand S.; Li P.; Lin C.; Luchko T.; Luo R.; Madej B.; Mermelstein D.; Merz K. M.; Monard G.; Nguyen H.; Nguyen H. T.; Omelyan I.; Onufriev A.; Roe D. R.; Roitberg A.; Sagui C.; Simmerling C. L.; Botello-Smith W. M.; Swails J.; Walker R. C.; Wang J.; Wolf R. M.; Wu X.; Xiao L.; Kollman P. A. AMBER 2016; University of California: San Francisco, 2016.

[ref65] Maier J. A.; Martinez C.; Kasavaghala K.; Wickström L.; Hauser K. E.; Simmerling C. L. FF14SB: Improving the Accuracy of Protein Side Chain and Backbone Parameters from FF99SB. J. Chem. Theory Comput. 2015, 11, 3696–3713. 10.1021/acs.jctc.5b00255. 26574453 PMC4821407

[ref66] Jorgensen W. L.; Chandresekhar J.; Madura J. D.; et al. Comparison of Simple Potential Functions for Simulating Liquid Water. J. Chem. Phys. 1983, 79, 926 10.1063/1.445869.

[ref67] Schneider T.; Stoll E. Molecular-Dynamics Study of a Three-Dimensional One- Component Model for Distortive Phase Transitions. Phys. Rev. B 1978, 17, 1302–1322. 10.1103/PhysRevB.17.1302.

[ref68] Berendsen H. J. C.; Postma J. P. M.; van Gunsteren W. F.; DiNola A.; Haak J. R. Molecular-Dynamics with Coupling to an External Bath. J. Chem. Phys. 1984, 81, 3684–3690. 10.1063/1.448118.

[ref69] Warshel A.; Weiss R. M. An Empirical Valence Bond Approach for Comparing Reactions in Solutions and in Enzymes. J. Am. Chem. Soc. 1980, 102, 6218–6226. 10.1021/ja00540a008.

[ref70] Blaha-Nelson D.; Krüger D. M.; Szeler K.; Ben-David M.; Kamerlin S. C. L. Active Site Hydrophobicity and the Convergent Evolution of Paraoxonase Activity in Structurally Divergent Enzymes: The Case of Serum Paraoxonase 1. J. Am. Chem. Soc. 2017, 139, 1155–1167. 10.1021/jacs.6b10801. 28026940 PMC5269640

[ref71] Ben-David M.; Soskine M.; Dubovetskyi A.; Cherukuri K.-P.; Dym O.; Sussman J. L.; Liao Q.; Szeler K.; Kamerlin S. C. L.; Tawfik D. S. Enzyme Evolution: An Epistatic Ratchet versus a Smooth Reversible Transition. Mol. Biol. Evol. 2020, 37, 1133–1147. 10.1093/molbev/msz298. 31873734

[ref72] Amrein B. A.; Bauer P.; Duarte F.; Janfalk Carlsson Å.; Naworyta A.; Mowbray S. L.; Widersten M.; Kamerlin S. C. L. Expanding the Catalytic Triad in Epoxide Hydrolases and Related Enzymes. ACS Catal. 2015, 5, 5702–5713. 10.1021/acscatal.5b01639. 26527505 PMC4613740

[ref73] Bauer P.; Janfalk Carlsson Å.; Amrein B. A.; Dobritzsch D.; Widersten M.; Kamerlin S. C. L. Conformational Diversity and Enantioconvergence in Potato Epoxide Hydrolase 1. Org. Biomol. Chem. 2016, 14, 5639–5651. 10.1039/C6OB00060F. 27049844 PMC5315018

[ref74] Marelius J.; Kolmodin K.; Feierberg I.; Åqvist J. Q: A Molecular Dynamics Program for Free Energy Calculations and Empirical Valence Bond Simulations in Biomolecular Systems. J. Mol. Graphics Modell. 1998, 16, 213–225. 10.1016/S1093-3263(98)80006-5. 10522241

[ref75] Bauer P.; Barrozo A.; Purg M.; Amrein B. A.; Esguerra M.; Wilson P. B.; Major D. T.; Åqvist J.; Kamerlin S. C. L. *Q*6: A Comprehensive Toolkit for Empirical Valence Bond and Related Free Energy Calculations. SoftwareX 2018, 7, 388–395. 10.1016/j.softx.2017.12.001.

[ref76] Jorgensen W. L.; Maxwell D. S.; Tirado-Rives J. Development and Testing of the OPLS All-Atom Force Field on Conformational Energetics and Properties of Organic Liquids. J. Am. Chem. Soc. 1996, 118, 11225–11236. 10.1021/ja9621760.

[ref77] Roe D. R.; Cheatham T. E. III PTRAJ and CPPTRAJ: Software for Processing and Analysis of Molecular Dynamics Trajectory Data. J. Chem. Theory Comput. 2013, 9, 3084–3095. 10.1021/ct400341p. 26583988

[ref78] Schmidtke P.; Bidon-Chanal A.; Luque F. J.; Barril X. MDpocket: Open-Source Cavity Detection and Characterization on Molecular Dynamics Trajectories. Bioinformatics 2011, 27, 3276–3285. 10.1093/bioinformatics/btr550. 21967761

[ref79] Sterner R.; Kleemann G. R.; Szadkowski H.; Lustig A.; Hennig M.; Kirschner K. Phosphoribosyl Anthranilate Isomeraes from *Thermotoga maritima* is an Extremely Stable and Active Homodimer. Protein Sci. 1996, 5, 2000–2008. 10.1002/pro.5560051006. 8897600 PMC2143258

[ref80] Noda-García L.; Juárez-Vázquez A. L.; Ávila-Arcos M. C.; Verduzco-Castro E. A.; Montero-Morán G.; Gaytán P.; Carrillo-Tripp M.; Barona-Gómez F. Insights Into the Evolution of Enzyme Substrate Promiscuity After the Discovery of (βα)_8_ Isomerase Evolutionary Intermediates from a Diverse Metagenome. BMC Evol. Biol. 2015, 15, 107 10.1186/s12862-015-0378-1. 26058375 PMC4462073

[ref81] Hennig M.; Sterner R.; Kirschner K.; Jansonius J. N. Crystal Structure at 2.0 Å Resolution of Phosphoribosyl Anthranilate Isomerase from the Hyperthermophile *Thermotoga maritima*: Possible Determinants of Protein Stability. Biochemistry 1997, 36, 6009–6016. 10.1021/bi962718q. 9166771

[ref82] Shen R.; Crean R. M.; Olsen K. J.; Richan T.; Brandão T. A. S.; Berry R. D.; Tolman A.; Loria J. P.; Johnson S. J.; Kamerlin S. C. L.; Hengge A. C. Insights into the Importance of WPD-Loop Sequence for Activity and Structure in Protein Tyrosine Phosphatases; ChemRxiv, 2021. 10.1039/d2sc04135a PMC9682893 36507179

[ref83] Dubey K. D.; Singh W. Simulations Reveal the Key Role of Arg15 in the Promiscuous Activity in the HisA Enzyme. Org. Biomol. Chem. 2021, 19, 10652–10661. 10.1039/D1OB02029C. 34854451

[ref84] Nursten H. The Maillard Reaction: Chemistry, Biochemistry and Implications; Royal Society of Chemistry: Great Britain, 2005.

[ref85] Hommel U.; Eberhard M.; Kirschner K. Phosphoribosyl Anthranilate Isomerase Catalyzes a Reversible Amadori Reaction. Biochemistry 1995, 34, 5249–5439. 10.1021/bi00016a014. 7727401

[ref86] Schlee S.; Klein T.; Schumacher M.; Nazet J.; Merkl R.; Steinhoff H.-J.; Sterner R. Relationship of Catalysis and Active Site Loop Dynamics in the (βα)8-Barrel Enzyme Indole-3-glycerol Phosphate Synthase. Biochemistry 2018, 57, 3265–3277. 10.1021/acs.biochem.8b00167. 29498826

[ref87] Barona-Gómez F.; Hodgson D. A. Occurence of a Putative Ancient-Like Isomerase Involved in Histidine and Tryptophan Biosynthesis. EMBO Rep. 2003, 4, 296–300. 10.1038/sj.embor.embor771. 12634849 PMC1315899

[ref88] Brändén C. The TIM Barrel—The Most Frequently Occuring Folding Motif in Proteins. Curr. Opin. Struct. Biol. 1991, 1, 978–983. 10.1016/0959-440X(91)90094-A.

[ref89] Katebi A. R.; Jernigan R. L. The Critical Role of the Loops of Triosephosphate Isomerase for Its Oligomerization, Dynamics and Functionality. Protein Sci. 2014, 23, 213–228. 10.1002/pro.2407. 24318986 PMC3926746

[ref90] Joseph D.; Petsko G.; Karplus M. Anatomy of a Conformational Change: Hinged “Lid” Motion of the Triosephosphate Isomerase Loop. Science 1990, 249, 1425–1428. 10.1126/science.2402636. 2402636

[ref91] Desamero R.; Rozovsky S.; Zhadin N.; McDermott A.; Callender R. Active Site Loop Motion in Triosephosphate Isomerase: T-Jump Relaxation Spectroscopy of Thermal Activation. Biochemistry 2003, 42, 2941–2951. 10.1021/bi026994i. 12627960

[ref92] Kadumuri R. V.; Vadrevu R. Diversity in αβ and βα Loop Connections in TIM Barrel Proteins: Implications for Stability and Design of the Fold. Interdiscip. Sci. 2018, 10, 805–812. 10.1007/s12539-017-0250-7. 29064074

[ref93] Liao Q.; Kulkarni Y.; Sengupta U.; Petrovic D.; Mulholland A. J.; van der Kamp M.; Strodel B.; Kamerlin S. C. L. Loop Motion in Triosephosphate Isomerase Is Not a Simple Open and Shut Case. J. Am. Chem. Soc. 2018, 140, 15889–15903. 10.1021/jacs.8b09378. 30362343

[ref94] Mhashal A. R.; Romero-Rivera A.; Mydy L. S.; Cristobal J. R.; Gulick A. M.; Richard J. P.; Kamerlin S. C. L. Modeling the Role of a Flexible Loop and Active Site Side Chains in Hydride Transfer Catalyzed by Glycerol-3-phosphate Dehydrogenase. ACS Catal. 2020, 10, 11253–11267. 10.1021/acscatal.0c02757. 33042609 PMC7536716

[ref95] Rozovsky S.; Jogl G.; Tong L.; McDermott A. E. Solution-State NMR Investigations of Triosephosphate Isomerase Active Site Loop Motion: Ligand Release in Relation to Active Site Loop Dynamics. J. Mol. Biol. 2001, 310, 271–280. 10.1006/jmbi.2001.4673. 11419952

[ref96] Rozovsky S.; McDermott A. E. The Time Scale of the Catalytic Loop Motion in Triosephosphate Isomerase. J. Mol. Biol. 2001, 310, 259–270. 10.1006/jmbi.2001.4672. 11419951

[ref97] Wang Y.; Berlow R.; Loria J. P. The Role of Loop-Loop Interactions in Coordinating Motions and Enzymatic Function in Triosephosphate Isomerase. Biochemistry 2009, 48, 4548–4556. 10.1021/bi9002887. 19348462 PMC2713366

[ref98] Radzicka A.; Wolfenden R. A Proficient Enzyme. Science 1995, 267, 90–93. 10.1126/science.7809611. 7809611

[ref99] Knowles J. R.; Albery W. J. Perfection in Enzyme Catalysis: The Energetics of Triosephosphate Isomerase. Acc. Chem. Res. 1977, 10, 105–111. 10.1021/ar50112a001.

[ref100] Knowles J. R. Enzyme Catalysis: Not Different, Just Better. Nature 1991, 350, 121–124. 10.1038/350121a0. 2005961

[ref101] Claren J.; Malisi C.; Höcker B.; Sterner R. Establishing Wild-Type Levels of Catalytic Activity on Natural and Artificial (βα)8-Barrel Protein Scaffolds. Proc. Natl. Acad. Sci. U.S.A. 2009, 106, 3704–3709. 10.1073/pnas.0810342106. 19237570 PMC2656144

[ref102] Lang D.; Thoma R.; Henn-Sax M.; Sterner R.; Wilmanns M. Structural Evidence for Evolution of the β/α Barrel Scaffold by Gene Duplication and Fusion. Science 2000, 289, 1546–1550. 10.1126/science.289.5484.1546. 10968789

[ref103] Campbell E. C.; Kaltenbach M.; Correy G. J.; Carr P. D.; Probeski B. T.; Livingstone E. K.; Afriat-Jurnou L.; Buckle A. M.; Weik M.; Hollfelder F.; Tokuriki N.; Jackson C. J. The Role of Protein Dynamics in the Evolution of New Enzyme Function. Nat. Chem. Biol. 2016, 12, 944–950. 10.1038/nchembio.2175. 27618189

[ref104] Ochoa-Leyva A.; Montero-Morán G.; Saab-Rincón G.; Brieba L. G.; Soberón X. Alternative Splice Variants in TIM Barrel Proteins from Human Genome Correlate with the Structural and Evolutionary Modularity of this Versatile Protein Fold. PLoS One 2013, 8, e70582 10.1371/journal.pone.0070582. 23950966 PMC3741200

