# Supplementary material for: Complex Loop Dynamics Underpin Activity, Specificity, and Evolvability in the (βα)8 Barrel Enzymes of Histidine and Tryptophan Biosynthesis
Source: JACS Au. 2022 Apr 4;2(4):943–60. doi: 10.1021/jacsau.2c00063 (PMC9088769; doi:10.1021/jacsau.2c00063)
Supplement: Supplementary file 1 — au2c00063_si_001.pdf [file au2c00063_si_001.pdf]

Supporting Information for:

Complex Loop Dynamics Underpin Activity, Specificity and Evolvability in the $(\beta\alpha)_8$ Barrel Enzymes of Histidine and Tryptophan Biosynthesis

Adrian Romero-Rivera,[1,‡,#] Marina Corbella,[1,‡] Antonietta Parracino,[1] Wayne M. Patrick[2] and Shina Caroline Lynn Kamerlin[1,*]

1. Department of Chemistry – BMC, Uppsala University, BMC Box 576, S-751 23 Uppsala, Sweden. 2. Centre for Biodiscovery, School of Biological Sciences, Victoria University of Wellington, Wellington 6012, New Zealand.

Corresponding author email addresses: lynn.kamerlin@kemi.uu.se

Table of Contents

S1. Supplementary Methodology……………………………………………………………………..**S3**

System Setup for Conventional and Enhanced Sampling Molecular Dynamics Simulations……S3

Ligand Parameterization for Conventional and Enhanced Sampling Molecular Dynamics Simulations…………………………………………………………………………..…S4

Conventional Molecular Dynamics Simulations……………………………………………..S5

Equilibration Procedure for the Conventional Molecular Dynamics Simulations……………...S5

Enhanced Sampling Molecular Dynamics Simulations……………………………………....S6

System Preparation for the Empirical Valence Bond Simulations………………………………S7

Parameterization and Calibration of the Empirical Valence Bond Simulations………………...S8

Empirical Valence Bond Simulations…………………………………………………………S9

Simulation Analysis…………………………………………………………..……..S11

S2. Supplementary Figures………………………………………………………………..…**S13**

S3. Supplementary Tables…………………………………………………………..……....**S29**

S4. Supplementary References………………………………………………………..…..**S48**

S1. Supplementary Methodology

System Setup for Conventional and Enhanced Sampling Molecular Dynamics Simulations

Simulations were performed (**Table S1**) of the enzymes TrpF (*Thermotoga maritima,* dimer), PriA (*Mycobacterium tuberculosis*, monomer) and HisA (*Salmonella enterica*, monomer) in both their unliganded forms, and in complex with substrates ProFAR and PRA, using PDB IDs: 1NSJ[1, 2] (unliganded *Tm*TrpF), 1LBM[2, 3] (*Tm*TrpF in complex with PRA in one monomeric unit), 2Y89[2, 4] (unliganded *Mt*PriA, D11N variant), 2Y88[2, 4] (*Mt*PriA in complex with PRFAR, D11N variant), 3ZS4[2] (wild-type *Mt*PriA in complex with PRFAR), 2Y85[2, 4] (wild-type *Mt*PriA in complex with RCdRP), 5AHE[2, 5] (unliganded *Se*HisA), 5A5W[2, 5] (*Se*HisA in complex with ProFAR, D7N/D176A variant), 5G5I[2, 6] (*Se*HisA(D10G)), 5G2I[2, 6] (*Se*HisA(dup13-15)), 5AC8[2, 6] (*Se*HisA(dup13-15/D10G/G102A)), 5G1Y[2, 6] (*Se*HisA(dup13-15/D10G/V14bM/Q24L/G102A)), 5AC7[2, 6] (*Se*HisA(dup13-15/D7N/D10G)), and 5AB3[2, 6] (*Se*HisA(dup13-15/D7N/D10G/Q24L/G102A), see **Table S1** for a full list of structures used). In the latter two structures, the catalytic loops are closed over the active site, whereas in the other HisA structures, the loops are found in open conformations. In the case of PDB IDs: 2Y89[2, 4] (unliganded *Mt*PriA, D11N variant), 5A5W[2, 5] (*Se*HisA in complex with ProFAR, D7N/D176A variant) and 5AC7[2, 6] (*Se*HisA(dup13-15/D7N/D10G)), the substitutions D7N, D11N and D176A were reverted for our simulations using the Dunbrack 2010 Rotamer Library,[7] as implemented in UCSF Chimera, v. 1.14.[8] In each case, the relevant rotamers to revert to were selected by comparison to the positioning of these side chains in the corresponding loop-open forms of the relevant enzymes. PDB IDs: 2Y85,[2, 4] 5AHE,[2, 5] 5G5I,[2, 6] 5G2I,[2, 6] 5G1Y,[2, 6] and 5AB3[2, 6] have missing regions in the catalytic loops; these were reconstructed by using Modeller v. 9.23,[9] and taking the lowest energy conformation prediction after visual inspection in each case.

Protonation states of ionizable residues and protonation patterns of histidine side chains (protonating at the ε- and δ-positions or in both positions) were determined by use of PROPKA 3.1,[10] and necessary

changes were incorporated into the structure using Maestro v. 11.8.[11] The catalytic aspartic acid side chain in the active site of each enzyme (D176 in *Se*HisA, D175 in *Mt*PriA and D126 in *Tm*TrpF) was kept protonated in line with the mechanism shown in **Figure 1**; only H50 in *Mt*PriA was predicted to be doubly protonated at physiological pH, while all the other side chains were kept in their default protonation states at physiological pH. The substrate ProFAR was manually placed into the relevant active sites in the same conformation as found in the structure of the HisA wild-type enzyme in complex with ProFAR (PDB ID: 5A5W[2, 5]). In the case of the smaller substrate, PRA (**Figure 1D**), the substrate was manually placed into the relevant active sites by manual overlay of the reactive part of PRA with the reactive part of ProFAR, and with the carboxylate group of PRA interacting with a nearby active site arginine (R143 for *Mt*PriA, R36 for *Tm*TrpF, R15b for *Se*HisA(dup1315/D10G/G102A/Q24L)), which forms a key interaction which appears to be necessary for proper substrate binding, based on examination of available crystal structures.

Ligand Parameterization for Conventional and Enhanced Sampling Molecular Dynamics Simulations

Partial charges for the ligands ProFAR, PRA, PRFAR and CdRP, were calculated using the standard restrained electrostatic potential (RESP) protocol, using Antechamber v. 17.3.[12] The electrostatic potential of each ligand was determined *in vacuo* at the HF/6-31G(d) level of theory, using Gaussian 09 Rev. E.01,[13] after geometry optimization at the same level of theory. All other simulation parameters were described using the general Amber force field 2 (GAFF2)[14] with the relevant parameters provided in **Tables S3** to **S6**. These parameters were then used for all conventional and enhanced sampling molecular dynamics simulations. In the case of the EVB simulations, these simulations were performed using the OPLS-AA force field[15] and therefore separate EVB parameters were derived as described in the section **Parameterization and Calibration of the Empirical Valence Bond Simulations**.

Conventional Molecular Dynamics Simulations

All systems were solvated in an octahedral box of TIP3P water molecules, extended 10Å from the closest solute molecule in all directions. Each system was neutralized by the addition of a system-specific number of Na^+ and Cl^- counterions (depending on total system charge, which is -3 for wild-type HisA, -13 for wild-type PriA and -2 for wild-type TrpF). Counterions were placed using the "addions" approach as implemented in AMBER 16,[16] which consists of drawing a grid around the solute and placing the ions at the grid points with the lowest energies. The protein was described using the AMBER ff14SB force field,[17] and the relevant substrates, ProFAR and PRA, as well as the products PRFAR and CdRP, were described by parameters obtained using the General AMBER Force Field 2 (GAFF2).[14] The partial charges for both substrates were calculated using the standard restrained electrostatic potential (RESP) approach, using Antechamber v. 17.3,[12] and based on the vacuum electrostatic potential calculated at the HF/6-31G(d) level of theory, using Gaussian 09 Rev. E.01.[13] The GAFF2 parameters and partial charges of substrates ProFAR and PRA as well as products PRFAR and CdRP, are listed in **Table S3** to **S6**. Finally, to keep the substrate stably bound in the enzyme active sites, in particular for simulations with the catalytic loops in their open conformations, we applied weak distance restraints to protein-substrate distances, as described in **Table S7**. Note that we were particularly careful to not include restraints to any loop residues in order to not hamper the conformational sampling of the catalytic loops.

Equilibration Procedure for the Conventional Molecular Dynamics Simulations

The LEaP module of AMBER 16[16] was used to generate the topology and coordinate files for the conventional MD simulations, which were performed using the CUDA version of the PMEMD module of the AMBER 16 simulation package.[16] The solvated system was first subjected to a 5000 step steepest descent minimization, followed by a 5000 step conjugate gradient minimization, with positional restraints placed on all heavy atoms of the solute, using a 5 kcal mol^{-1} $Å^{-2}$ harmonic potential. The minimized system was then heated up to 300 K using the Berendsen thermostat,[18] with a time constant of 1 ps for the coupling, and once again applying 5 kcal mol^{-1} $Å^{-2}$ harmonic positional restraints during

the heating process. These restraints were then gradually decreased to 1 kcal mol^{-1} $Å^{-2}$ over five steps of NPT equilibration (500 ps each), using the Berendsen thermostat and barostat[18] to keep the system at 300K and 1 atm. For the production runs, each system was subjected to 10 x 500 ns of molecular dynamics simulations, controlled by the Langevin thermostat with a collision frequency of 2 ps^{-1},[19] and the Berendsen barostat with a 1 ps coupling constant.[18] A 10 Å cutoff was applied to all non-bonded interactions, with the electrostatic interactions being treated using the particle mesh Ewald (PME) approach. A summary of all conventional MD simulations (time scale and number of replicas per system) performed in this work is shown in **Table S1**, and the root mean square deviations (RMSD) of all backbone atoms for each system during the 500 ns production runs is shown in **Figures S11** and **S12**.

Enhanced Sampling Molecular Dynamics Simulations

Steered molecular dynamics simulation (sMD) were applied using GROMACS 2018.4 in order to pull the product (center of mass of all heavy atoms) from the bottom of the TIM-barrel scaffold (center of mass of C_α from residues L49, R101, H170, and G224), in order to explore loop dynamics upon product release from the active site of *Se*HisA(dup13-15/D10G/G102A/Q24L). Bond lengths of hydrogen atoms were constrained using P-LINCS,[20] and temperature was controlled using the Berendsen thermostat.[18] The Particle-mesh Ewald algorithm[21] was employed to calculate electrostatic interactions, using a cutoff of 10 Å. Energy minimization of the system was performed using 2000 steps of the steepest descent algorithm, with a tolerance of 1000 kJ mol^{-1} nm^{-1}. After minimization, NVT and NPT equilibration were performed for 5 ns for each system. 10 replicas of production MD were performed for 50 ns. After the first 5 ns of production MD, we applied an external force with a force constant of 10 kcal mol^{-1} $Å^{-2}$ to pull the product out of the active site, increasing the distance between the center of mass of the substrate/product and the bottom of the TIM-barrel scaffold from 14.3 Å (initial measured distance from starting structure) to 30 Å over the next 40 ns of simulation time. This external force was then released for the last 5 ns of the MD simulation run.

System Preparation for the Empirical Valence Bond Simulations

Empirical valence bond (EVB) simulations were performed to study the enzyme-catalyzed opening of the ribose ring of substrates ProFAR and PRA (**Figure 1**), as catalyzed by wild-type and variant forms of *Se*HisA and *Mt*PriA. Specifically, simulations were performed on wild-type *Se*HisA and *Mt*PriA, as well as the S202A, D129N, D10G, dup13-15, dup13-15/D10G, dup13-15/D10G/G102A, dup13-15/D10G/Q24L/G102A and dup13-15/D10G/V14bM/Q24L/G102A variants of *Se*HisA, and the R19A, D130A, and R143A variants of *Mt*PriA. In the case of wild-type PriA, as well as the dup13-15/D10G/V14bM/Q24L/G102A variant of HisA, simulations were performed with loop 1 in both catalytically competent closed conformations and the corresponding loop-open conformations for comparison, to examine the effect on the corresponding calculated activation barriers.

In the case of the wild-type enzymes, simulations were performed using PDB ID: 5A5W,[2, 5] in the case of *Se*HisA and PDB IDs: 3ZS4[2] and 2Y85[2, 4], as starting points for simulations of substrate ProFAR and PRA in the *Mt*PriA active site, respectively. In the case of SeHisA(dup13-15/D10G/V14bM/Q24L/G102A), simulations were performed using PDB ID: 5AB3.[2, 6] In the case of other variants studied in this work, all glycine/alanine substitutions were performed by manual side chain deletion using the respective wild-type enzyme as a starting point, and for the other substitutions, by use of the Dunbrack 2010 Rotamer Library,[7] as implemented in UCSF Chimera, v. 1.14.[8] As for the conventional/enhanced sampling molecular dynamics simulations, in each case, the relevant rotamers to revert to were selected by comparison to the positioning of these side chains in the corresponding loop-open forms of the relevant enzymes. In addition, in all cases where the relevant substrate was not already present in the active site in the starting crystal structure used, substrates were manually placed in the active site such that they maintained a catalytic distance (~2.4 Å) between the oxygen of the ribose ring and the protonated side chain of the catalytic aspartic acid (D176 in *Se*HisA and D175 in *Mt*PriA). In addition, substrates were positioned in order to either ensure the formation of a stacking interaction with the side chain of the active site tryptophan W145 and the imidazole ring of ProFAR, or a salt-bridge

between the carboxylate group of PRA and the side chain of a key active site arginine (R15b in the *Se*HisA(dup13-15) variants and R143 in wild-type *Mt*PriA).

All systems were solvated in a 30Å sphere of TIP3P water molecules,[22] subjected to surface-constrained all-atom solvent (SCAAS) boundary conditions.[23] Within this framework, the system is described in a multilayer fashion, in which all atoms within the inner 85% of the solvent sphere are allowed to move freely, while atoms in the external 15% of the sphere, and those that fall outside the explicit solvent sphere, are subject to 10 and 200 kcal mol^{-1} $Å^{-2}$ harmonic positional restraints to restrict their mobility, as is standard within the SCAAS model. The solvent sphere was centered on the C_{γ} carbon atom of the catalytic aspartic acid in each system, in order to ensure that our EVB simulations fully capture the flexibility of the residues of loops 1, 5 and 6, and that the loops do not fall within the restrained region of the simulations. Finally, with the exception of the catalytic acid (**Figure 1**), all titratable residues that fell within the inner 85% of the sphere (*i.e.* within 25.5Å from the simulation center) were kept in their default ionization states at pH 7, based on pK_a estimates using PROPKA 3.1,[10] and all other residues were kept in their neutral forms in order to avoid system instabilities caused by inclusion of charge outside the explicit solvation sphere. For a list of protonation states of titratable residues as well as histidine protonation patterns in our simulations, see **Table S14**. Due to the high negative charge of the system that fits inside the water droplet in the case of HisA, 9 Na^+ counterions were added in order to neutralize the system. These counterions were placed so that they interact with negatively charged residues near the surface of the enzyme, that still fall within the water droplet. Note that neither the number nor the position of the counterions was changed in any of the other systems, in order to avoid any effect on the calculated energies derived from the inclusion of positively charged counterions.

Parameterization and Calibration of the Empirical Valence Bond Simulations

One of the key tenets of the EVB approach is the use of a well-defined reference reaction, which can be for example either the non-enzymatic reaction in either vacuum or aqueous solution, or the reaction catalyzed by the wild-type reaction as a benchmark against a series of enzyme variants.[24, 25] The EVB

parameters (and, in particular, the EVB off-diagonal element, H_{ij}, and the gas-phase shift, α, which are described in more detail in refs. [24, 25]) are then calibrated to fit data from either high-level quantum calculations or experiment, and the same parameter set is used unchanged to compare a series of variants. Due to the lack of experimental data to describe the non-enzymatic isomerization reactions of ProFAR and PRA, we used the activation free energies derived from the experimentally observed turnover numbers for the corresponding PriA catalyzed isomerizations of ProFAR and PRA, as upper limits for the activation-free energy for the corresponding ribose ring-opening step in this enzyme, performed fitting of the EVB parameters to reproduce these values (based on experimental data presented in refs. [4, 6]), and then used these parameters unchanged to describe the ribose ring-opening step in all other systems studied in this work, in order to facilitate direct comparison between different systems. The resulting EVB parameters for each substrate can be found in **Table S15**.

Partial charges for the corresponding intermediate states were calculated as described in the section **Ligand Parameterization for Conventional and Enhanced Sampling Molecular Dynamics Simulaions**. All other OPLS-AA-compatible force filed parameters were obtained using the "ffld_server", as implemented in Schrödinger's Macromodel suite,[26] and then converted to *Q*6 readable format using *Q*tools v0.5.10 (DOI: 10.5281/zenodo.842003). All EVB parameters necessary to reproduce our work have been uploaded to Zenodo and can be found at the following DOI: 10.5281/zenodo.5893598.

Empirical Valence Bond Simulations

All the EVB simulations in this work were done using the *Q6* simulation package.[27, 28] Temperature control was maintained in all simulations using the Berendsen thermostat,[18] with the solute and solvent coupled to individual heat baths independently. All simulations were performed using a 1 fs time step, unless stated otherwise, and using the leapfrog integrator. All bonds involving hydrogen atoms were constrained using the SHAKE algorithm.[29] Long-range electrostatic effects were described using the local reaction field (LRF) approach,[30] and all non-bonded interactions were calculated using a 10 Å

cutoff, with the exception of the reacting (EVB) atoms, which were subjected to an (in principle infinite) 99Å cutoff. All systems were initially briefly simulated for 3 ps, increasing the temperature from 0.01 to 1 K and increasing the timestep from 0.01 to 1 fs, in order to remove steric clashes and close contacts in the system. They were then heated gradually heated up to 300 K and cooled down again to 5 K, over 40 ps of simulation time, in order to equilibrate the water molecules in the explicit solvent sphere. The positions of all atoms were fixed during this initial stage of the simulation by use of a 200 kcal mol^{-1} $Å^{-2}$ harmonic restraint on all atoms in the system but the water molecules. The temperature was then gradually increased to 300 K while steadily removing the positional restraints on the system over a total of 160 ps of simulation time until the only restraints remaining in the mobile region of the sphere were a weak harmonic restraint of 0.5 kcal mol^{-1} $Å^{-2}$ on the reacting atoms (*i.e.* the side chain of the catalytic aspartic acid in loop 6, shown in **Figure 1**, and the substrate ProFAR or PRA), as well as 10 kcal mol^{-1} $Å^{-2}$ harmonic positional restraints on the Na^{+} counterions. This weak restraint was retained throughout the subsequent equilibration and EVB runs. Once the target temperature of 300 K had been reached, we performed a further 20 ns of unrestrained equilibration (apart from the aforementioned restraint on the reacting atoms), the endpoint of which was used as the starting point for propagating an EVB trajectory. Root mean square deviations of all protein backbone atoms during the equilibration of each system are shown in **Figures S13** and **14**.

The EVB free energy perturbation/umbrella sampling (EVB-FEP/US)[24] simulations were performed using the valence bond states shown in **Figure 3**, and each trajectory was simulated using 51 individual mapping windows of 200 ps of simulation time each, leading to a total of 10.2 ns of simulation time per individual EVB trajectory. Each simulation was repeated in 30 individual replicas, leading to a cumulative total of 600 ns equilibration and 306 ns EVB simulation time per individual system, and a cumulative 12 μs of equilibration and 6.12 μs of EVB simulation time over all 20 systems studied in this work.

Simulation Analysis

Active Site Volume Analysis

The active site volume of the unliganded wild-type enzymes (HisA, PriA and TrpF) was calculated along the corresponding conventional molecular dynamics simulations trajectories using the MDpocket[31] tool, published within the *fpocket*[32] suite of pocket detection programs. Snapshots for this analysis were taken every 0.5 ns of simulation time, resulting in a total of 1000 snapshots per trajectory (10000 snapshots total over 10 independent replicas). All cavities were identified using a frequency iso-value of 0.5. Points corresponding to the active site pocket were then selected and tracked throughout the trajectories to yield pocket volume *vs*. time.

Principal Component Analysis

Principal component analysis (PCA) produces orthogonal eigenvectors (principal components, PCs), that describe the variants of the different systems, with the resulting PCs ranked by the amount of variance they describe in the given dataset of MD simulations. Two sets of PCA were performed in this work: one on the C_α-carbon atoms of the 3 catalysis loops, and a second analysis where only the C_α-carbon atoms of loop 1 of the HisA variants carrying the dup13-15 elongation were considered.

In the case of the PCA of the 3 combined loops, we performed this analysis on MD simulations of each of HisA and PriA in their unliganded forms and in complex with substrates ProFAR and PRA, as well as simulations of unliganded and PRA-bound TrpF (see **Table S1** for a detailed description of the simulations performed in this work and corresponding PDB IDs. PCA was performed on each set of simulations using the CPPTRAJ[33] module of the AmberTools19[34] suite of programs. We first performed RMS of each simulation being considered onto the C_α-carbon atoms of all protein residues, using the corresponding loop closed state. The PCA was then performed on residues 11-25 (loop 1), 142-147 (loop5), and 172-182 (loop 6) of *Se*HisA, 15-25 (loop 1), 141-151 (loop 5), and 172-181 (loop 6) of *Mt*PriA, and 30-38 (loop 3) and 128-138 (loop 6) of *Tm*TrpF. In the case of the PCA of the HisA loop-elongated systems, the analysis was performed the combined conventional MD simulations of the

unliganded *Se*HisA(dup13-15), *Se*HisA(dup13-15/D10G), *Se*HisA(dup13-15/D10G/G120A) and *Se*HisA(dup13-15/D10G/G120A/Q24L/V15[b]M) variants (again, see **Table S1** for further details). In this case, the RMS fitting was performed using the *Se*HisA(dup13-15) variant with loop 1 in its open state as a reference, and then performing the PCA on residues 17-26 of each system.

Analysis of the Empirical Valence Bond Simulations

Analysis of root mean square deviations (RMSD) of all backbone atoms during our EVB simulations of various systems was performed using the AmberTools19.[34] All other analyses were performed using the *QCalc* module of the *Q6* simulation package.[27, 28] RMSD clustering was performed on substrate and key catalytic/stabilizing residues, using the hierarchical agglomerative algorithm as implemented in CPPTRAJ,[33] in order to obtain representative structures of each of the key reacting species from each simulation (Michaelis complex, transition state for the ring-opening reaction, and subsequent intermediate state). The centroids of the top ranked cluster of each state were then used to produce **Figures 10** (ProFAR) and **S10** (PRA).

S2. Supplementary Figures

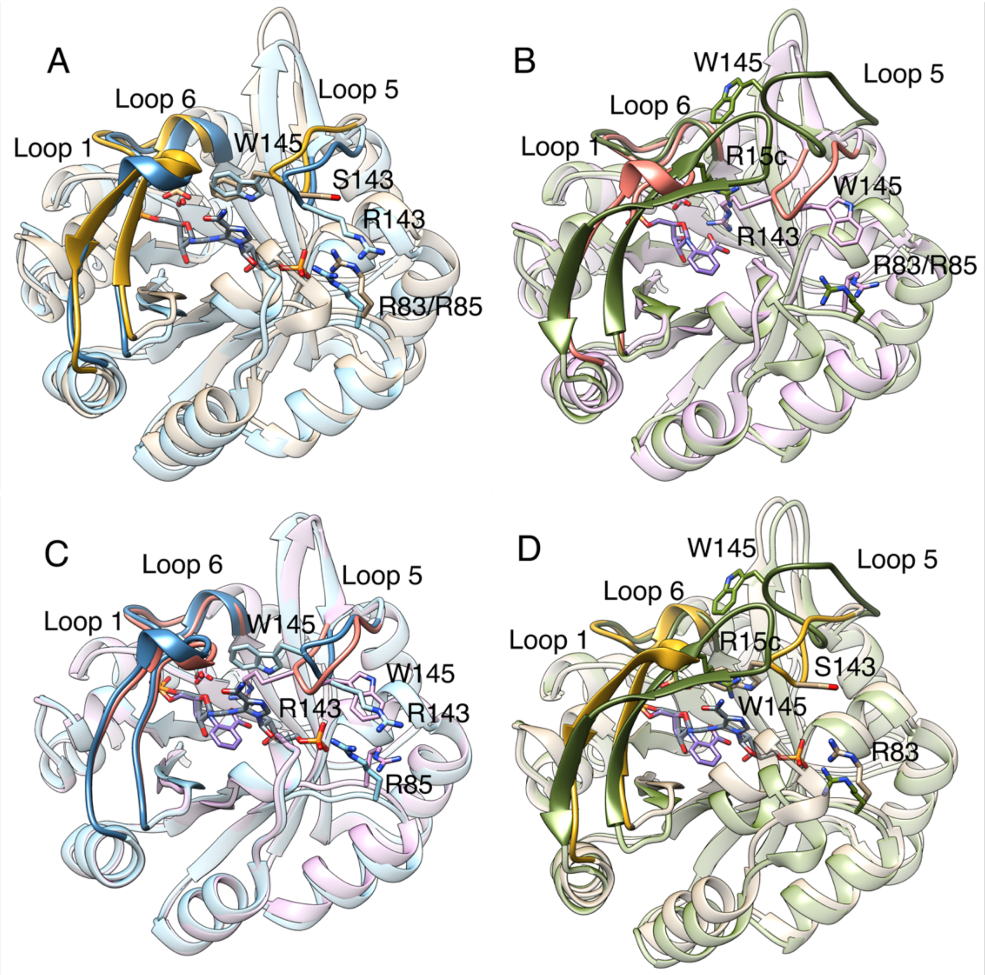

Figure S1. Structural comparison between the crystallographic conformations of key catalytic loops in (**A**) wild-type *Se*HisA (light brown, PDB ID: 5A5W[2, 5]) and wild-type *Mt*PriA in its pro-ProFAR conformation (cyan, PDB ID: 3ZS4[2]), (**B**) *Se*HisA(dup13-15) (green, PDB ID: 5AC7[2, 6]) and wild-type *Mt*PriA in its pro-PRA conformation (pink, PDB ID: 2Y85[2, 4]), (**C**) wild-type *Mt*PriA in its pro-ProFAR (cyan, PDB ID: 3ZS4[2]) and pro-PRA (pink, PDB ID: 2Y85[2, 4]) conformations and **(D)** wild-type *Se*HisA (light brown, PDB ID: 5A5W[2, 5]) and *Se*HisA(dup13-15) (green, PDB ID: 5AC7[2, 6]). The corresponding catalytic loops are shown in a darker version of the scaffold colors. The catalytic aspartic acid side chain in loop 1, as well as W145 and R143 (S143 in wild-type HisA) from loop 5 and the "gripper" arginine side chains (R83 in HisA and R85 in PriA), are shown as sticks. Both ProFAR and PRA substrates, in the corresponding active sites, are depicted as sticks in grey and purple, respectively.

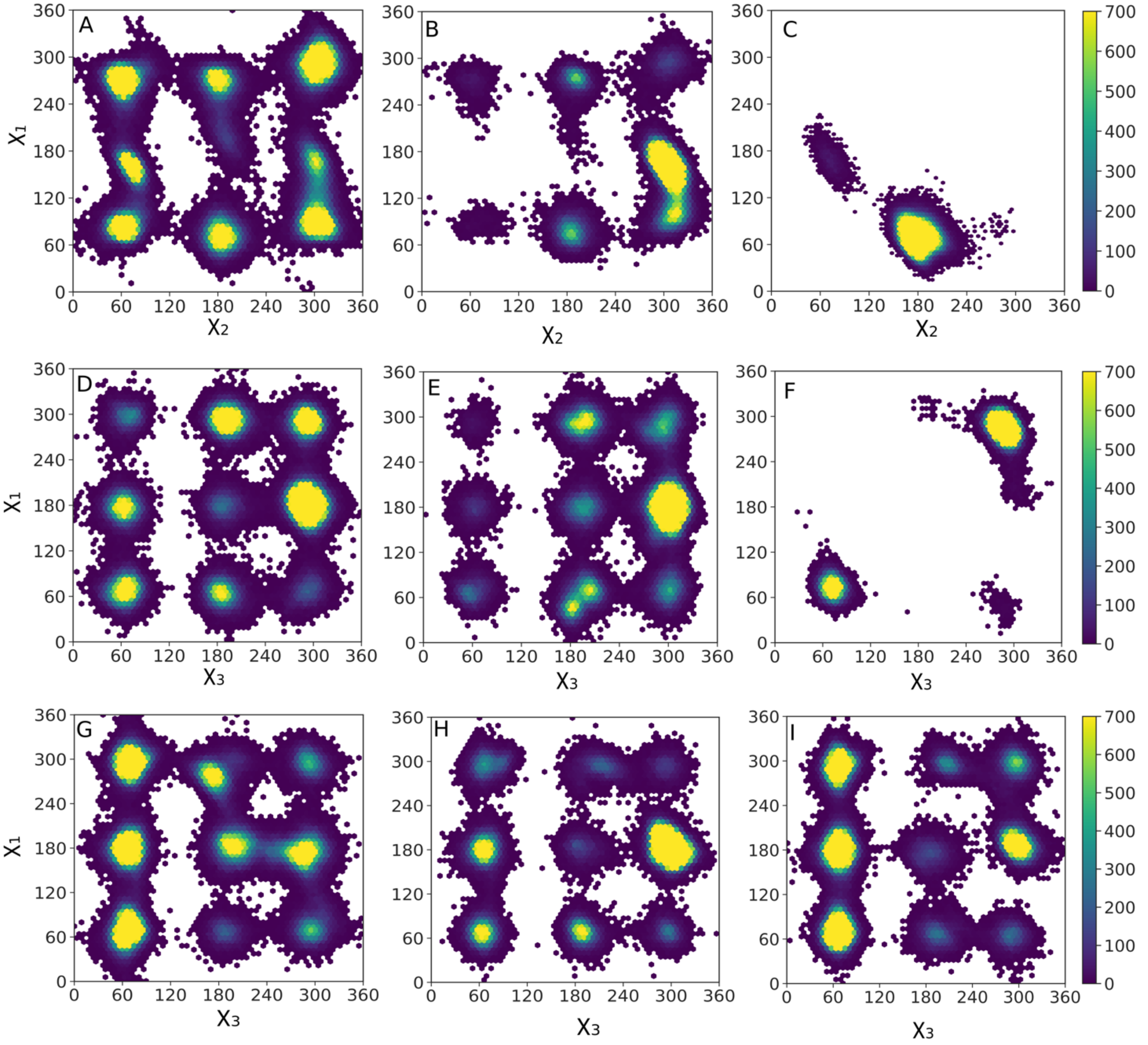

Figure S2. Joint distribution of the side chain dihedral angles of the side chains of (**A**, **B**, **C**) W145, (**D**, **E**, **F**) R143 and (**G**, **H**, **I**) R85 in (**A**, **D**, **G**) unliganded PriA, (**B**, **E**, **H**) PriA in complex with substrate ProFAR and (**C**, **F**, **I**) PriA in complex with substrate PRA. Data was extracted every 5 ps of 10 x 500 ns production simulations of each system, performed as described in the main text.

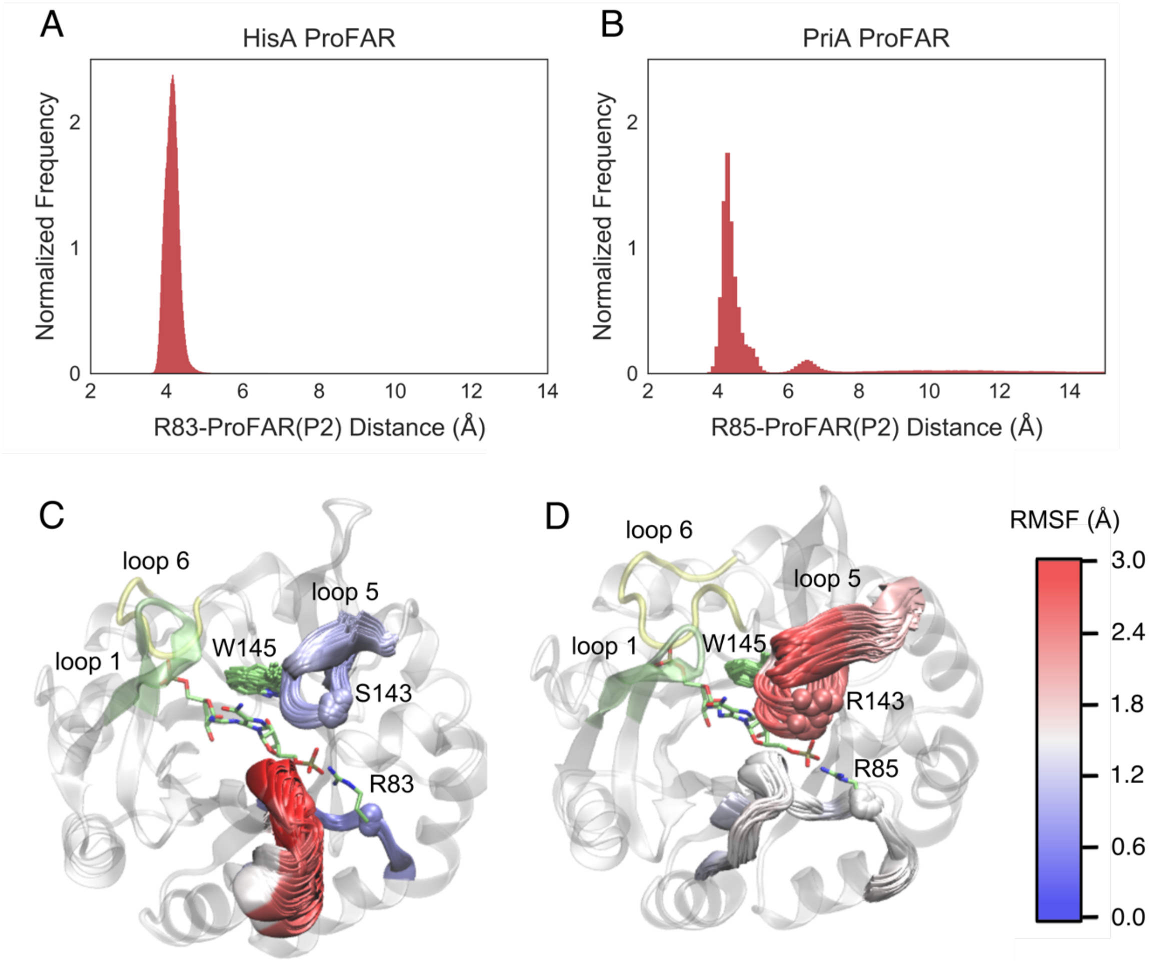

Figure S3. Differences in the structural stability of loop 5 and the “gripper” arginine of HisA and PriA with substrate ProFAR from our conventional MD simulations. (**A**, **B**) Normalized histograms of the distance between the “gripper” arginine (R83/R85) and the second phosphodianion group of substrate ProFAR in (**A**) HisA and (**B**) PriA. (**C**, **D**) Snapshots from our simulations illustrating the conformational sampling/diversity of the loops surrounding the second phosphodianion group of the substrate ProFAR in (**C**) HisA and (**D**) PriA. The loops are colored mapped from red (most flexible) through white and to blue (least flexible) according to their calculated C_α RMSF. Loops 1 and loop 6 and shown in green and yellow in their starting conformations, respectively. R85 and R143, which electrostatically repel each other in PriA due to their close proximity, are shown as spheres for reference, as well as the corresponding residues R83 and S143 in HisA. W145, which is involved in the stacking interaction with the substrate ProFAR is shown as green sticks in both enzymes.

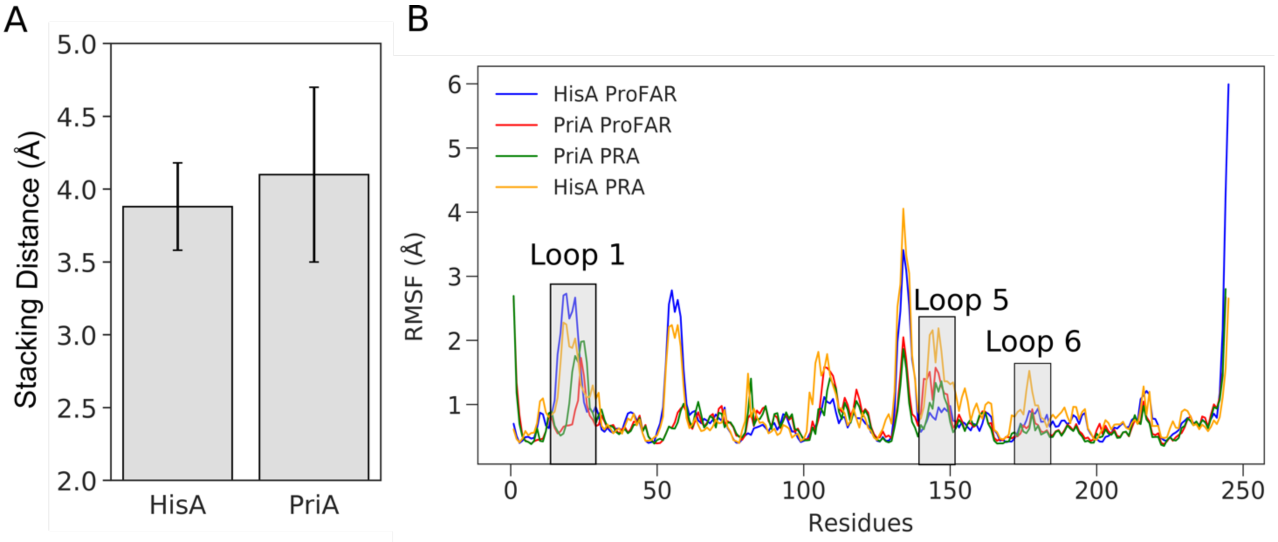

Figure S4. (**A**) Distance between the center of mass of the imidazole ring of ProFAR and the center of mass of the active site tryptophan side chain stabilizing the imidazole ring (W145 for HisA and PriA), shown as average values and standard deviations across 10 x 500 ns conventional MD simulations of each system. These correspond to average values of 3.9 ± 0.3 Å in HisA, and 4.1 ± 0.6 Å in PriA. (**B**) Root mean square fluctuation (RMSF) of HisA with liganded ProFAR (blue), PriA with liganded ProFAR (red), and PriA with liganded PRA (green).

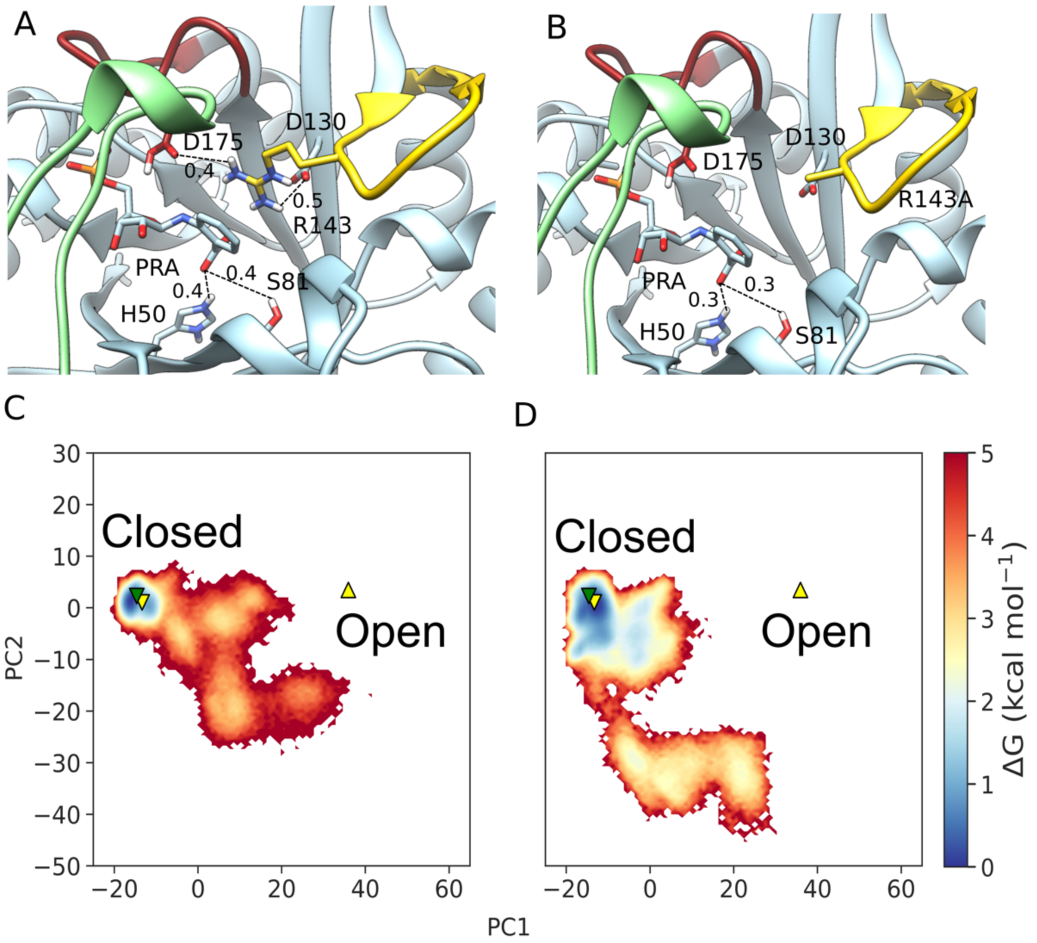

Figure S5. Starting structures for our simulations of (**A**) wild-type PriA and (**B**) the PriA R143A variant, in complex with substrate PRA, prepared as described in the **Methodology** section). This figure highlights the main interactions between the carboxylate fragment of the substrate with its surrounding residues, as well as the interactions between the arginine side chain (R143 in wild-type PriA) with its surrounded residues. The numbers indicate the fraction of simulation time each interaction is maintained. A complete set of interactions with a description of how these values were obtained is provided in **Table S9**. (**C**, **D**) Projected free energy surfaces (kcal mol^{-1}) along the first two principal components (PC1 and PC2), obtained from applying Cartesian principal component analysis (PCA) to our conventional MD simulations of (**C**) wild-type PriA and (**D**) PriA R143A variant, in complex with substrate PRA. Free energies were estimated from the bin populations of the 2D histograms (using 100 bins on each axis), employing the formula $G_i = -k_B T(N_i/N_{Max})$, where k_B is the Boltzmann constant, T is the temperature at

which the simulations were performed (300K), N_i is the population of each bin and N_{Max} is the population of the most populated bin. The open and closed conformations observed in the crystal structure are shown as yellow triangles.

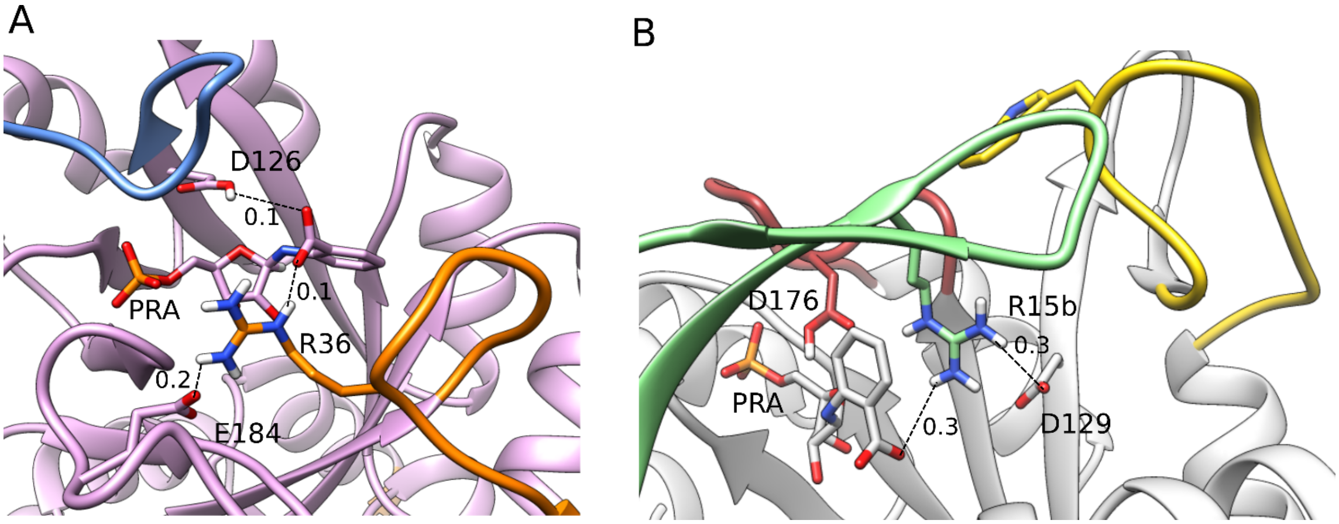

Figure S6. Starting structures for our simulations of (**A**) wild-type *Tm*TrpF and (**B**) the *Se*HisA(dup13-15/D10G/Q24L/G102A) variant, in complex with substrate PRA, prepared as described in the **Methodology** section). This figure highlights the main interactions between the carboxylate fragment of the substrate with its surrounding residues, as well as the interactions between the arginine side chain (36 in wild-type TrpF and R15b in the HisA variant) with its surrounded residues. The numbers indicate the fraction of simulation time each interaction is maintained. A complete set of interactions with a description of how these values were obtained is provided in **Table S9**.

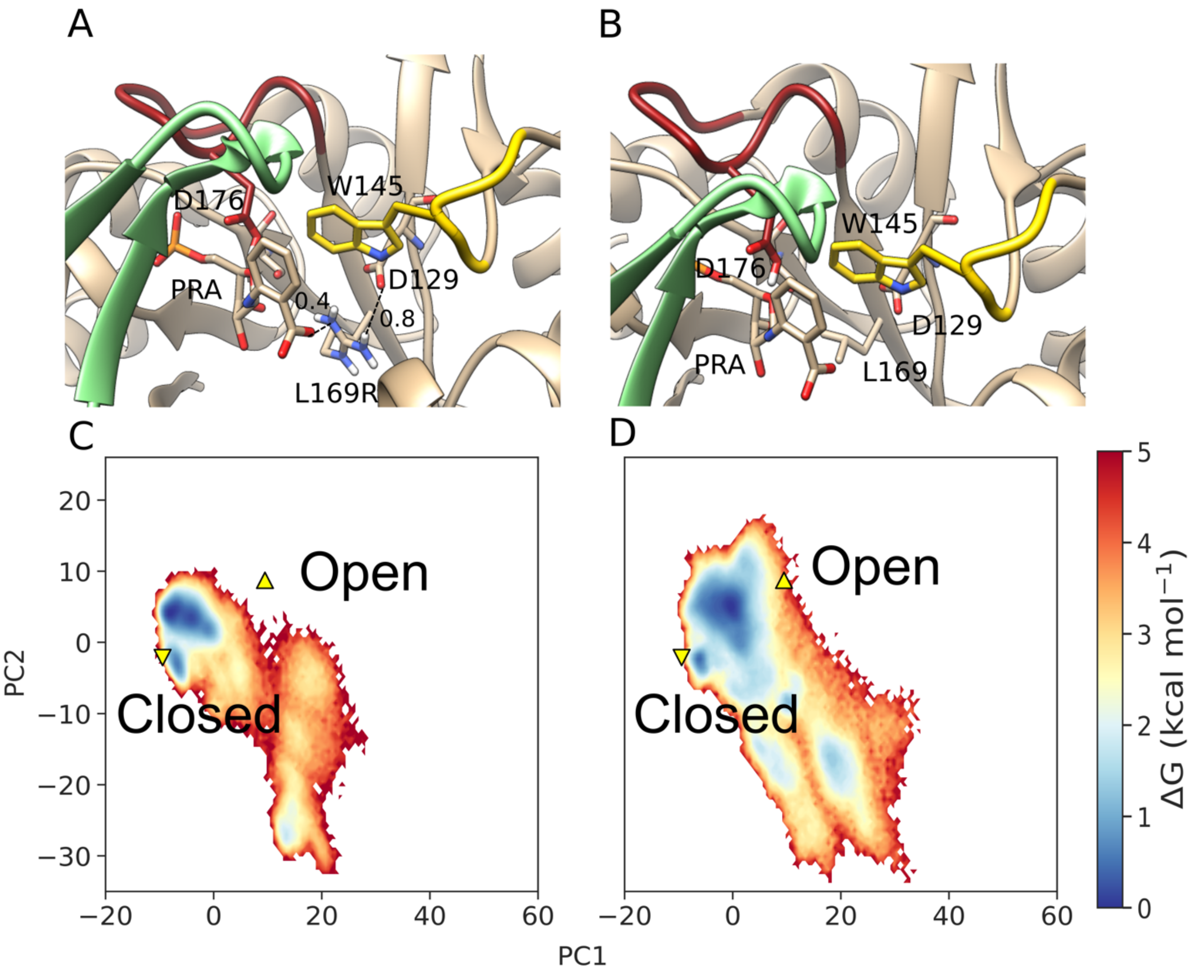

Figure S7. Starting structures for our simulations of (**A**) the *Se*HisA(L169R) variant, and (**B**) wild-type *Se*HisA, in complex with substrate PRA, prepared as described in the **Methodology** section). This figure highlights the main interactions between the carboxylate fragment of the substrate with its surrounding residues, as well as the interactions between the arginine side chain (R169 in the *Se*HisA(L169R) variant) with its surrounded residues. The numbers indicate the fraction of simulation time each interaction is maintained. A complete set of interactions with a description of how these values were obtained is provided in **Table S9**. (**C**, **D**) Projected free energy surfaces (kcal mol^{-1}) along the first two principal components (PC1 and PC2), obtained from applying Cartesian principal component analysis (PCA) to our conventional MD simulations of (**C**) the *Se*HisA(L169R) variant and (**D**) wild-type *Se*HisA, in complex with substrate PRA. Free energies were estimated from the bin populations of the 2D histograms (using 100 bins on each axis) employing the formula $G_i = -k_BT(N_i/N_{Max})$, where k_B is the Boltzmann

constant, T is the temperature at which the simulations were performed (300K), N_i is the population of each bin and N_{Max} is the population of the most populated bin. The open and closed conformations observed in the crystal structure are shown as yellow triangles.

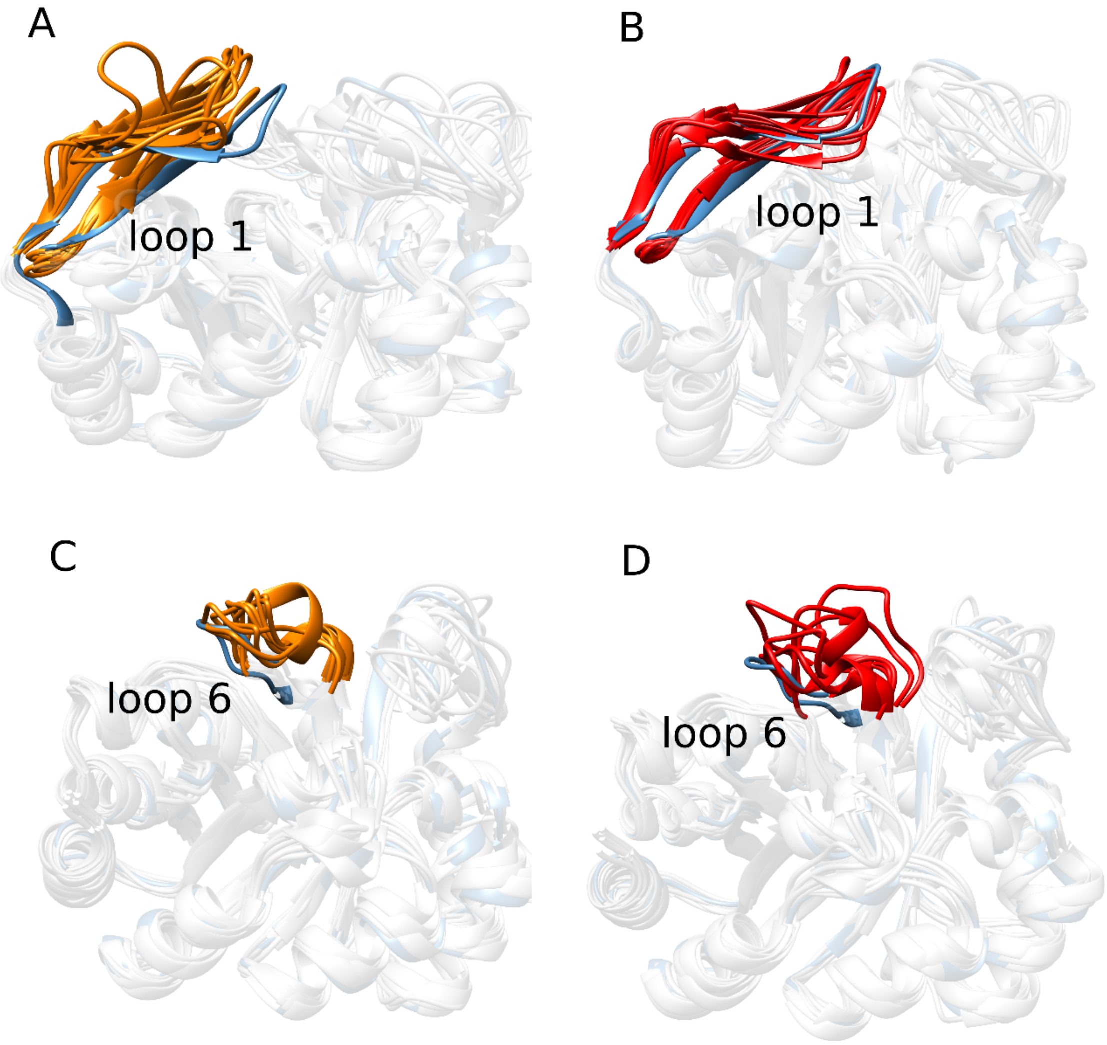

Figure S8. Overlay of the last frame from the 10 independent steered MD simulation of the release of products (**A**, **C**) PRFAR and (**B**, **D**) CdRP from the *Se*HisA(dup1315/D10G/G102A/Q24L) variant (*i.e.* 10 independent simulation structures per system). The starting structure used (PDB ID: 5AB3[2, 6]), in which loops 1 and 6 are closed, is highlighted in blue.

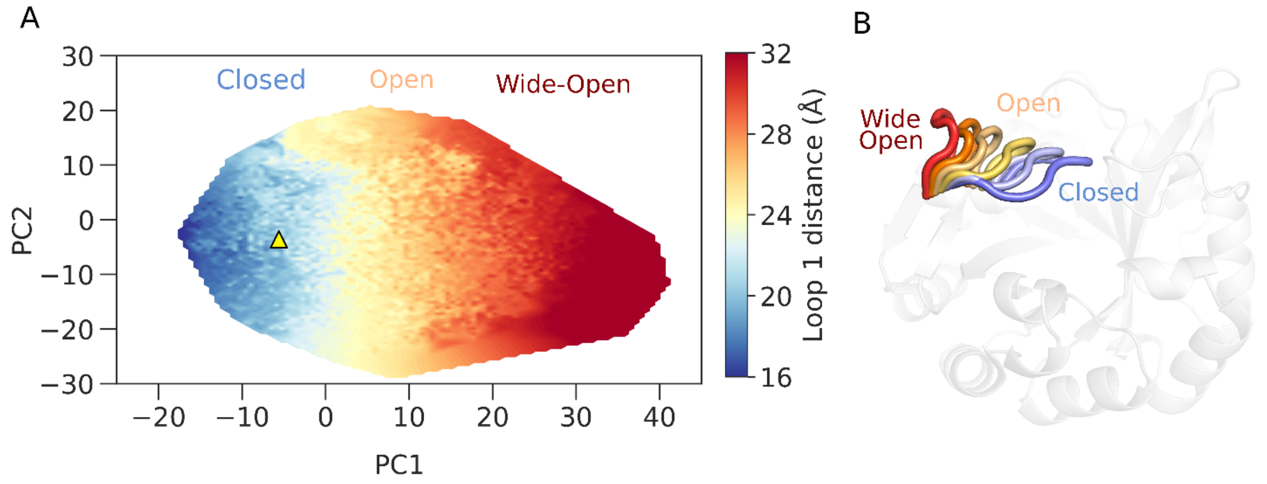

Figure S9. (**A**) Global projected loop 1 distance surfaces (Å) along the first two principal components (PC1 and PC2) obtained from applying Cartesian PCA to our conventional MD simulations of unliganded wild-type *Se*HisA and the *Se*HisA(D10G) variant. The starting loop 1 open state is indicated on this surface by a yellow triangle, ▲, and the same starting loop 1 conformation is used for simulations of both systems. Note that this figure considers the motion of loop 1 along the PCs, as shown for PC1, projections along which include transitions from closed to wide-open states of loop 1 (**B**). The PCA analysis was performed on the mass-weighted Cartesian coordinates of the HisA variants compared to the coordinates of the corresponding closed state, allowing us to explore the variation of the conformations of these loops in coordinate space. The loop 1 distance is defined as the center of mass of residues 15-25 of loop 1 and residue 129 from the barrel scaffold, of PC1 and PC2. Here, we considered distances <22.0 Å as corresponding to closed states (blue), between 23.0 - 28.0 Å as corresponding to open states (yellow), and >29.0 Å as corresponding to wide-open conformations (red), based on a combination of visual inspection and comparison of the closed state crystal structures (PDB IDs: 5AB3[2,6] and 5AC7[2,6]), and using the *Se*HisA(dup13-15) variant as a reference. A 1 Å difference between states was applied to avoid fuzzy states between transitions from the closed to the open state and from the open to the wide-open state.

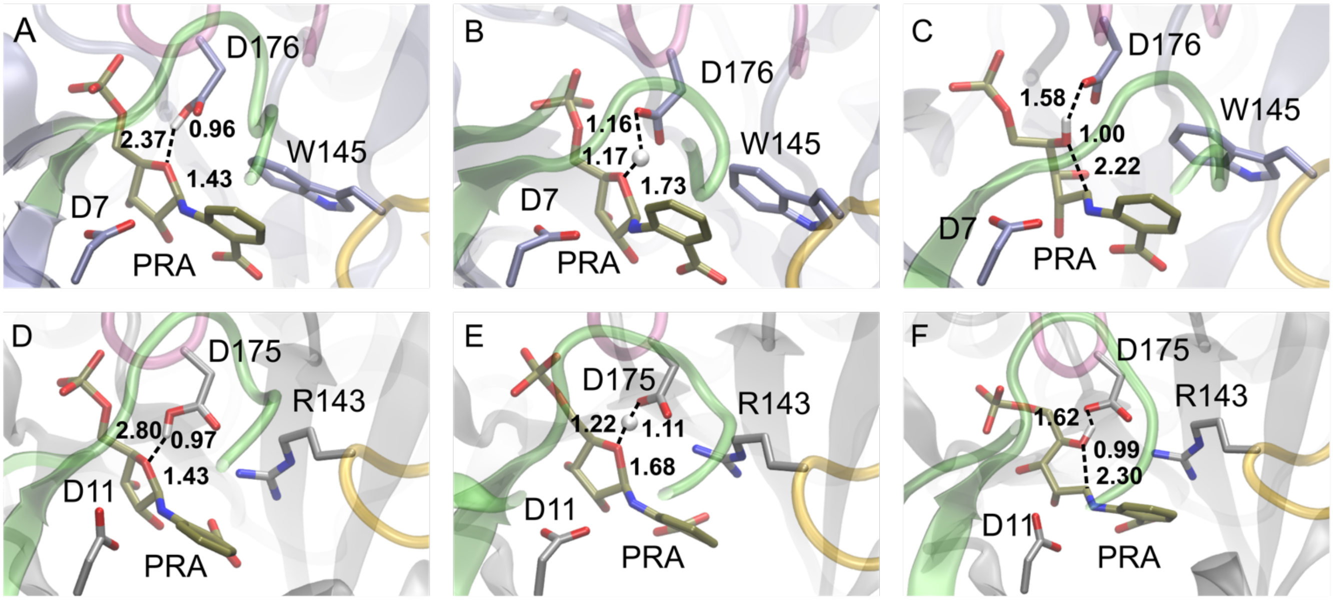

Figure S10. Representative structures of stationary points at the Michaelis complexes (MC), transition states (TS) and intermediate states (IS), respectively, for the ribose-ring opening step of the (**A, B, C**) HisA- and (**D, E, F**) PriA-catalyzed isomerization of PRA (by the wild-type enzymes). For EVB simulation details, see the **Methodology** section. Structures were selected based on clustering analysis using the hierarchical agglomerative algorithm, as implemented in CPPTRAJ[33] Note that the annotated catalytic distances are average values over 6000 snapshots extracted for each state from our EVB trajectories (from 30 x individual 200 ps EVB mapping windows per stationary point/system). For a full list of reacting distances across all variants, see **Tables S12** and **13**.

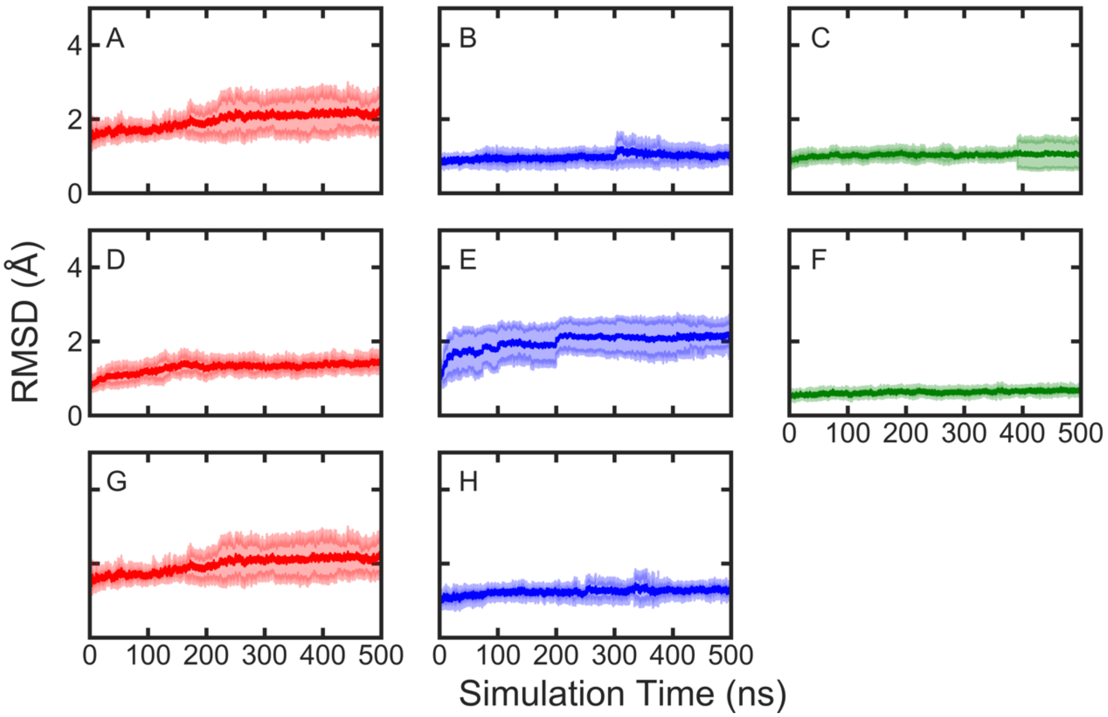

Figure S11. Root mean square deviations (RMSD, Å) of all backbone heavy atoms from MD simulations of (**A**) unliganded, (**B**) PRA-bound and (**C**) ProFAR bound *Mt*PriA, (**D**) unliganded, (**E**) PRA-bound and (**F**) ProFAR-bound *Se*HisA, and (**G**) unliganded and (**H**) PRA-bound *Tm*TrpF. Data was collected every 10 ps from 10 replicas of 500 ns length each. The solid lines show rolling averages of the RMSD over all 10 replicas, and the shaded regions show the corresponding standard deviations in these values.

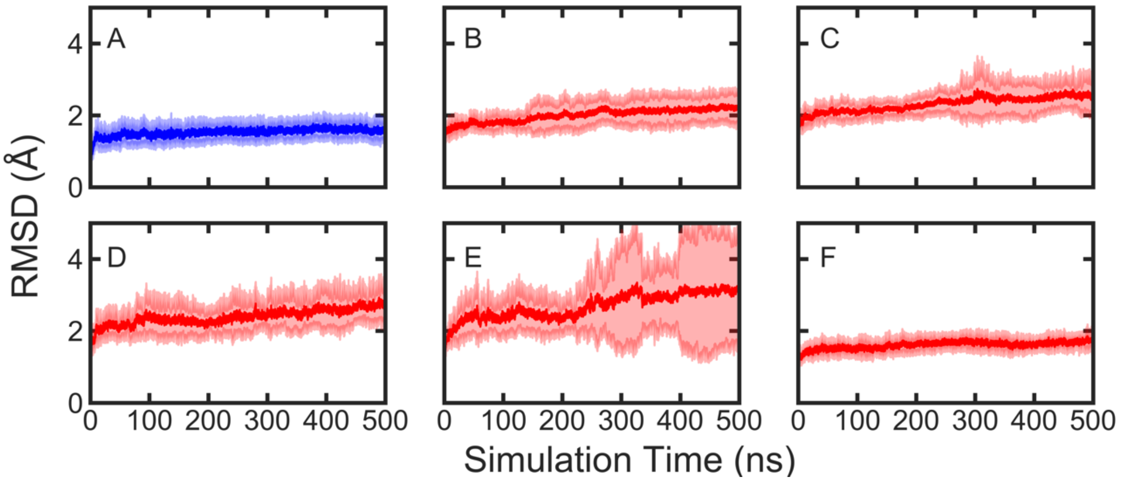

Figure S12. Root mean square deviations (RMSD, Å) of all backbone heavy atoms from MD simulations of (**A**) the PRA-bound *Se*HisA(L169R) variant, (**B**) the unliganded *Se*HisA(D10G) variant, (**C**) the unliganded *Se*HisA(dup13-15) variant, (**D**) the unliganded *Se*HisA(dup13-15/D10G) variant, (**E**) the unliganded *Se*HisA(dup13-15/D10G/G102A) variant, and (**F**) the unliganded *Se*HisA(dup13-15/D10G/G102A/Q24L/V15[b]M) variant. Data was collected every 10 ps from 10 replicas of 500 ns length each. The solid lines show rolling averages of the RMSD over all 10 replicas, and the shaded regions show the corresponding standard deviations in these values.

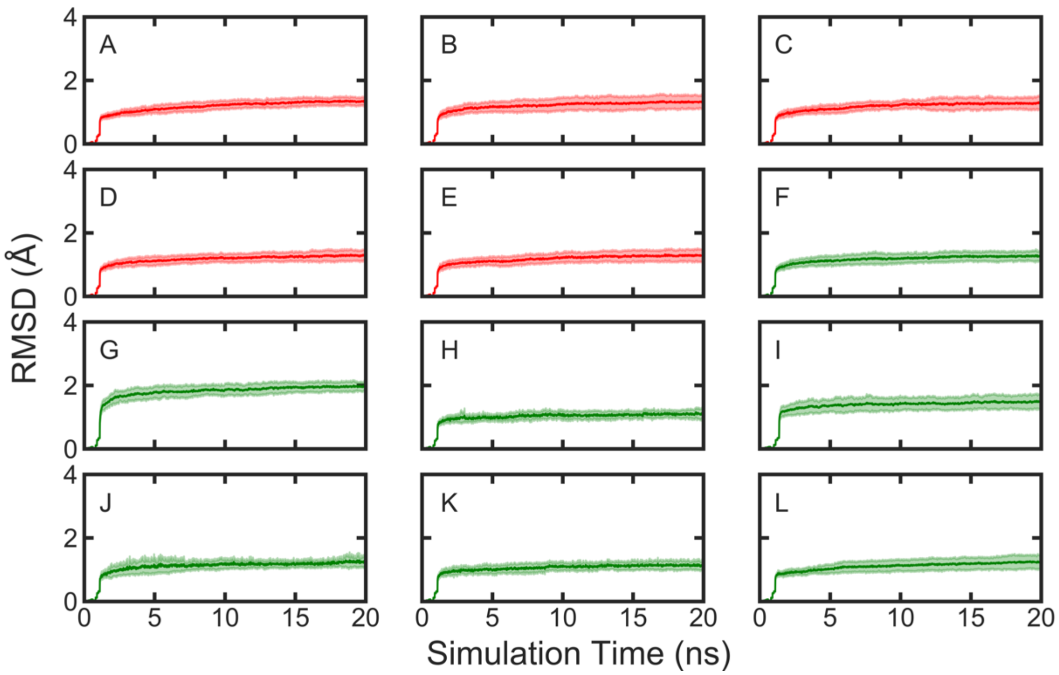

Figure S13. Root mean square deviations (RMSD, Å) of all backbone heavy atoms from EVB equilibration simulations of the ring-opening of PRA, as catalyzed by (**A**) wild-type *Mt*PriA with loop 1 in a closed conformation, (**B**) wild-type *Mt*PriA with loop 1 in an open conformation, the **(C**) R19A, (**D**) D130A, and (**E**) R143A PriA variants, as well as (**F**) wild-type HisA and the (**G**) dup13-15, (**H**) dup13-15/D10G, (**I**) dup13-15/D10G/G102A, (**J**) dup13-15/D10G/G102A/Q24L, (**K**) dup13-15/D10G/G102A/Q24L/V15[b]M (loop 1 closed) and (**L**) dup13-15/D10G/G102A/Q24L/V15[b]M (loop 1 open) *Se*HisA variants. PriA variants are shown in red, and HisA variants in green, for clarity. Data was collected every 10 ps from 30 replicas of 20 ns length each. The solid lines show rolling averages of the RMSD over all 10 replicas, and the shaded regions show the corresponding standard deviations in these values.

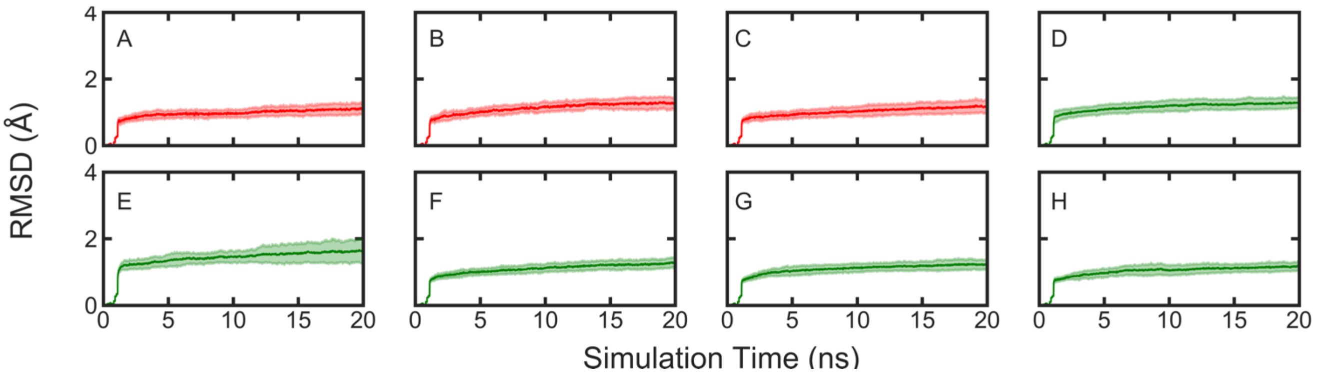

Figure S14. Root mean square deviations (RMSD, Å) of all backbone heavy atoms from EVB equilibration simulations of the ring-opening of ProFAR, as catalyzed by (**A**) wild-type *Mt*PriA and the (**B**) R19A and (**C**) D130A *Mt*PriA variants, as well as (**D**, **E**) wild-type *Se*HisA with loop 1 in closed and open conformations, respectively, as well as the (**F**) D10G (**G**) D129N and (**H**) S202A *Se*HisA variants. PriA variants are shown in red, and HisA variants in green, for clarity. Data was collected every 10 ps from 30 replicas of 20 ns length each. The solid lines show rolling averages of the RMSD over all 10 replicas, and the shaded regions show the corresponding standard deviations in these values.

S3. Supplementary Tables

Table S1. Summary of all MD simulations performed in the work.

Systems: Enzyme	Ligand	PDB ID	Individual Simulations (replicas x ns)	Total Simulation Time (μs)
Conventional MD simulations				
*Mt*PriA	Unliganded	2Y89[4]	10 × 500	5
	ProFAR	3ZS4[2]/2Y88[4]	10 × 500	5
	PRA	2Y85[4]	10 × 500	5
*Tm*TrpF	Unliganded	1NSJ[1]	10 × 500	5
	PRA	1LBM[3]	10 × 500	5
*Se*HisA	Unliganded	5AHE[2, 5]	10 × 500	5
	ProFAR	5A5W[5]	10 × 500	5
	PRA	5A5W[5]	10 × 500	5
*Se*HisA(D10G)	Unliganded	5G5I[6]	10 × 500	5
*Se*HisA(L169R)	PRA	5A5W[5]	10 × 500	5
*Se*HisA(dup13-15)	Unliganded	5G2I[6]	10 × 500	5
*Se*HisA(dup13-15/D10G)	Unliganded	5AC7[6]	10 × 500	5
*Se*HisA(dup13-15/D10G/G102A)	Unliganded	5AC8[6]	10 × 500	5
*Se*HisA(dup13-15/D10G/G102A/Q24L/V15[b]M)	Unliganded	5G1Y[6]	10 × 500	5
Enhanced Sampling MD simulations				
*Se*HisA(dup13-15/D10G/G102A/Q24L)	PRFAR	5AB3[6]	10 × 50	0.5
*Se*HisA(dup13-15/D10G/G102A/Q24L)	CdRP	5AB3[6]	10 × 50	0.5
**Total Simulation Time**				71

Table S2. Experimental kinetic parameters for all enzymes studied in this work.[a]

Enzyme	HisA Activity			TrpF Activity		
	k_{cat} (s^{-1})	K_M (μM)	k_{cat}/K_M (s^{-1} M^{-1})	k_{cat} (s^{-1})	K_M (μM)	k_{cat}/K_M (s^{-1} M^{-1})
***Se*HisA**						
**Wild-Type**	7.8 ± 2.4	17 ± 0.1	4.5×10^5	None	None	None
**D10G**	7.6 ± 0.1	29 ± 10	2.6×10^5	None	None	None
**dup13-15**	None	None	None	>0.15	>2,000	75 ± 2
**dup13-15/D10G**	0.05 ± 0.01	1.7 ± 0.2	2.8×10^4	0.09 ± 0.02	2,100 ± 1,000	51 ± 14
**dup13-15/D10G/G102A**	0.05 ± 0.02	5.7 ± 1.6	9.2×10^3	>0.44	>2,000	220 ± 30
**dup13-15/D10G/ /G102A/Q24L**	0.05 ± 0.01	10 ± 2	5.1×10^3	>0.52	>2,000	260 ± 30
**dup13-15/D10G/ /G102A/Q24L/V15[b]M**	None	None	None	>3.6	>2,000	$(1.8 \pm 0.1) \times 10^3$
***Mt*PriA**						
**Wild-Type**	0.23 ± 0.01	19 ± 3	1.2×10^4	3.6 ± 0.6	21 ± 2	1.7×10^5
**R19A**	0.071 ± 0.004	170 ± 20	4.1×10^2	1.0 ± 0.1	31 ± 2	3.3×10^4
**D130A**	0.01 ± 0.001	17 ± 5	6.0×10^2	1.4 ± 0.4	16 ± 5	9.1×10^4
**R143A**	n.d.	n.d.	2.4×10^2	n.d.	n.d.	6.0×10^3
***Tm*TrpF**						
**Wild-Type**	None	None	None	3.7	0.28	1.3×10^7

[a] Taken from experimental data presented in refs. [4-6, 35]. "n.d." indicates "not determined". Note that we also simulated the L169R variant in this work. This variant has not been included in the table due to the lack of kinetic data; however, relative growth rates on minimal media indicate that this variant is a TrpF-specialist.[36]

Table S3. Non-standard force field parameters used to describe the substrate ProFAR in our conventional and enhanced sampling molecular dynamics simulations.[a]

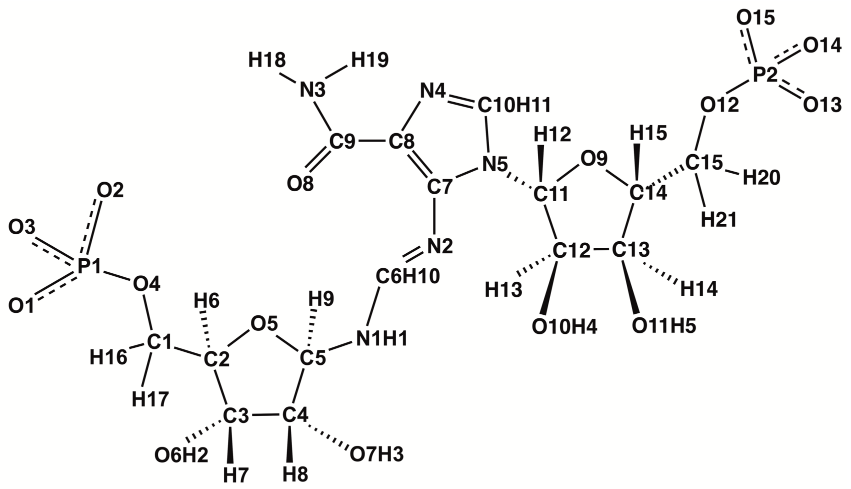

Atom Name	Atom Type	Charge	Atom Name	Atom Type	Charge
O1	o	-0.955265	O8	o	-0.697530
P1	p5	1.336924	N3	nt	-0.917422
O2	o	-0.955265	H18	hn	0.399747
O3	o	-0.955265	H19	hn	0.399747
O4	os	-0.647159	N4	nd	-0.63531
C1	c3	0.207236	C10	cc	0.215177
H16	h1	0.014131	H11	h5	0.209431
H17	h1	0.014131	N5	na	0.041397
C2	c3	0.183661	C11	c3	0.152892
C3	c3	0.340035	C12	c3	0.182788
O6	oh	-0.806662	O10	oh	-0.64782
H2	ho	0.438065	H4	ho	0.441778
C4	c3	0.199026	C13	c3	0.226096
O7	oh	-0.677423	O11	oh	-0.730937
H3	ho	0.298529	H5	ho	0.455426
H8	h1	0.022042	H14	h1	0.008723
H7	h1	0.079220	H13	h1	0.055619
H6	h1	0.072447	H12	h2	0.177249
O5	os	-0.639704	O9	os	-0.545927
C5	c3	0.626988	C14	c3	0.279845
H9	h2	-0.009441	H15	h1	0.096916
N1	nu	-0.678413	C15	c3	-0.028937
H1	hn	0.381766	H20	h1	0.062383
C6	c2	0.386143	H21	h1	0.062383
H10	h5	0.144377	O12	os	-0.559689
N2	ne	-0.390065	P2	p5	1.365000

C7	cc	0.000343	O14	o	-0.960341
C8	cd	0.029032	O15	o	-0.960341
C9	c	0.752563	O13	o	-0.960341

[a] All parameters were obtained using the General AMBER Force Field 2 (GAFF),[14] as outlined in the main text.

Table S4. Non-standard force field parameters used to describe the substrate PRA in our conventional and enhanced sampling molecular dynamics simulations.[a]

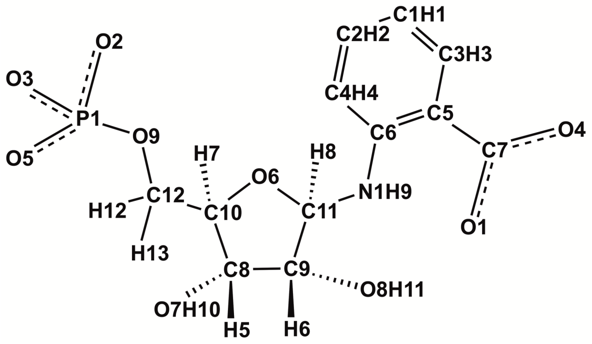

Atom Name	Atom Type	Charge	Atom Name	Atom Type	Charge
O1	o	-0.842154	C9	c3	0.061055
C7	c	0.871874	O8	oh	-0.796973
O4	o	-0.842154	H11	ho	0.488292
C5	ca	-0.283147	H6	h1	0.059818
C3	ca	-0.109958	C8	c3	0.335197
H3	ha	0.162349	O7	oh	-0.815427
C1	ca	-0.327949	H10	ho	0.489752
H1	ha	0.148974	H5	h1	-0.011569
C2	ca	-0.042353	C10	c3	0.284258
H2	ha	0.133908	H7	h1	0.011052
C4	ca	-0.481816	C12	c3	0.207962
H4	ha	0.223051	H12	h1	0.021947
C6	ca	0.598774	H13	h1	0.021947
N1	nu	-1.10638	O9	os	-0.628256
H9	hn	0.520030	P1	p5	1.299227
C11	c3	0.849779	O3	o	-0.919836
O6	os	-0.690857	O5	o	-0.919836
H8	h2	-0.050746	O2	o	-0.919836

[a] All parameters were obtained using the General AMBER Force Field 2 (GAFF),[14] as outlined in the main text.

Table S5. Non-standard force field parameters used to describe the product PRFAR in our conventional and enhanced sampling molecular dynamics simulations.[a]

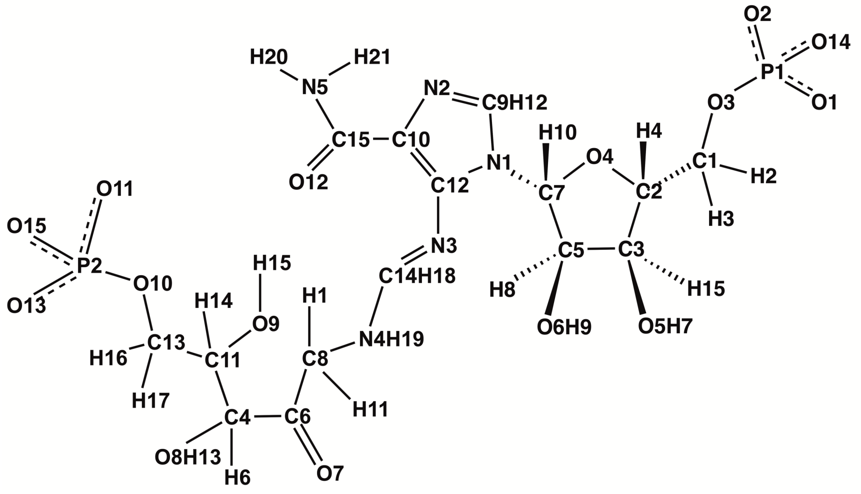

Atom Name	Atom Type	Charge	Atom Name	Atom Type	Charge
O1	o	-0.954583	H21	hn	0.396570
P1	p5	1.338731	O12	o	-0.680045
O2	o	-0.954583	C12	cc	0.019613
O14	o	-0.954583	N3	ne	-0.381121
O3	os	-0.554974	C14	c2	0.357672
C1	c3	0.008498	H18	h5	0.132851
H2	h1	0.055153	N4	nu	-0.503049
H3	h1	0.055153	H19	hn	0.359058
C2	c3	0.172313	C8	c3	-0.129132
H4	h1	0.107698	H1	h1	0.122829
C3	c3	0.279458	H11	h1	0.122829
H5	h1	0.002947	C6	c	0.606672
O5	oh	-0.741675	O7	o	-0.625622
H7	ho	0.449555	C4	c3	0.315244
C5	c3	0.322833	H6	h1	0.024370
H8	h1	0.009626	O8	oh	-0.786028
O6	oh	-0.733586	H13	ho	0.483187
H9	ho	0.345041	C11	c3	0.179868
O4	os	-0.521253	H14	h1	0.004443
C7	c3	0.245611	O9	oh	-0.719232
H10	h2	0.052248	H15	ho	0.416299
N1	na	0.016640	C13	c3	0.169940
C9	cc	0.182770	H16	h1	0.029756
H12	h5	0.215942	H17	h1	0.029756
N2	nd	-0.634022	O10	os	-0.576270
C10	cd	0.016967	P2	p5	1.327435

C15	c	0.759323	O13	o	-0.920003
N5	nt	-0.921700	O15	o	-0.920003
H20	hn	0.396570	O11	o	-0.920003

[a] All parameters were obtained using the General AMBER Force Field 2 (GAFF),[14] as outlined in the main text.

Table S6. Non-standard force field parameters used to describe the product CdRP in our conventional and enhanced sampling molecular dynamics simulations.[a]

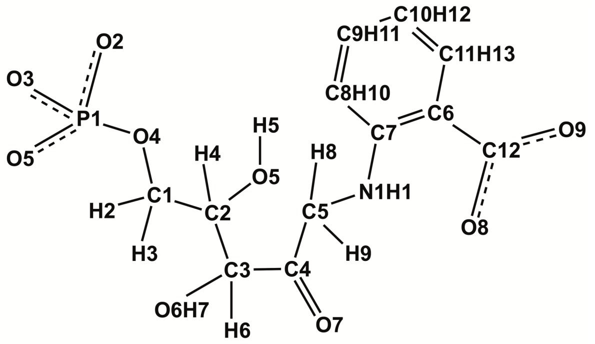

Atom Name	Atom Type	Charge	Atom Name	Atom Type	Charge
O1	o	-0.940107	C5	c3	0.076077
P1	p5	1.236201	H8	h1	0.051561
O2	o	-0.940107	H9	h1	0.051561
O3	o	-0.940107	N1	nu	-0.643338
O4	os	-0.533046	H1	hn	0.390509
C1	c3	0.138366	C7	ca	0.249311
H2	h1	0.031372	C6	ca	-0.096719
H3	h1	0.031372	C12	c	0.818684
C2	c3	0.221510	O8	o	-0.817135
H4	h1	-0.020474	O9	o	-0.817135
O5	oh	-0.688069	C11	ca	-0.218936
H5	ho	0.359250	H13	ha	0.176372
C3	c3	0.421032	C10	ca	-0.194309
H6	h1	-0.003458	H12	ha	0.140609
O6	oh	-0.735467	C9	ca	-0.167750
H7	ho	0.409446	H11	ha	0.157776
C4	c	0.376345	C8	ca	-0.211955
O7	o	-0.540435	H10	ha	0.171192

[a] All parameters were obtained using the General AMBER Force Field 2 (GAFF),[14] as outlined in the main text.

Table S7. Distance restrains applied during conventional molecular dynamics simulations of HisA, PriA and TrpF in complex with substrates ProFAR and PRA.[a]

**Systems**	**Distances**	r_1	r_2	r_3	r_4	K_2	K_3
HisA/ProFAR	D7:OD1…ProFAR:O7	1	1.5	3.2	3.8	5	5
	G204:N…ProFAR:O1	1	1.5	3.2	3.8	5	5
	R226:N…ProFAR:O2	1	1.5	3.2	3.8	5	5
	D129:OD2…ProFAR:O10	1	1.5	3.2	3.8	5	5
	S202:OG…ProFAR:O6	1	1.5	3.2	3.8	5	5
	G101:N…ProFAR:O13	1	1.5	3.2	3.8	5	5
	S103:N…ProFAR:O15	1	1.5	3.2	3.8	5	5
PriA/ProFAR	D11:OD1…ProFAR:O7	1	1.5	3.2	3.8	5	5
	K226N:ProFAR:O3	1	1.5	3.2	3.8	5	5
	G201:N…ProFAR:O2	1	1.5	3.2	3.8	5	5
	D129:OD2…ProFAR:O10	1	1.5	3.2	3.8	5	5
	S199:OG…ProFAR:O6	1	1.5	3.2	3.8	5	5
	T104:N…ProFAR:O13	1	1.5	3.2	3.8	5	5
	T104:OG…ProFAR:O15	1	1.5	3.2	3.8	5	5
HisA and HisA(L169R) PRA	D7:OD1…PRA:O8	1	1.5	3.2	3.8	5	5
	G204:N…PRA:O3	1	1.5	3.2	3.8	5	5
	R226:N…PRA:O5	1	1.5	3.2	3.8	5	5
	S202:OG…PRA:O7	1	1.5	3.2	3.8	5	5
PriA and PriA (R143A) PRA	D11:OD1…PRA:O8	1	1.5	3.2	3.8	5	5
	G201:N…PRA:O5	1	1.5	3.2	3.8	5	5
	K226:N…PRA:O2	1	1.5	3.2	3.8	5	5
TrpF/PRA	C7:SG…PRA:O8	1	1.5	3.2	3.8	5	5
	G159:N…PRA:O3	1	1.5	3.2	3.8	5	5
	S180:N…PRA:O2	1	1.5	3.2	3.8	5	5
	D178:OD2…PRA:O7	1	1.5	3.2	3.8	5	5
HisA(dup1315/D10G/G102A/Q24L) PRA	D7:OD1…PRA:O8	1	1.5	3.2	3.8	5	5
	G204:N…PRA:O3	1	1.5	3.2	3.8	5	5
	R226:N…PRA:O5	1	1.5	3.2	3.8	5	5
	S202:OG…PRA:O7	1	1.5	3.2	3.8	5	5

[a] For atom nomenclature in the different substrates, see the structures presented in **Tables S3** and **S4**. r_1 through r_4 denote the four distances used to define the constrains and K_2 and K_3 denote force constant used for the constrains. All distances are provided in Å and all force constants are presented in kcal mol^{-1} Å^{-2}.

Table S8. Comparison of the active site volumes of wild-type HisA, PriA and TrpF, and the corresponding turnover numbers of each enzyme towards ProFAR and PRA.[a]

		k_{cat} (s^{-1})	
**Enzyme**	**Active Site Volume (Å^3)**	**ProFAR**	**PRA**
HisA	1173.5 ± 158.0	7.8 ±2.4	N.A.
PriA	1033.8 ± 217.0	0.23	3.6
TrpF	745.4 ± 129.6	N.A.	3.7

[a] Active site volumes (Å^3) were calculated using MDpocket,[31] and are average values and standard deviations of volumes calculated over snapshots taken every 0.5 ns of 10 x 500 ns of conventional MD simulations per system. The corresponding turnover numbers for ProFAR and PRA for each system (k_{cat}, s^{-1}) are based on experimental data presented in refs. [4, 6, 35]. Note that "N.A." indicates no observed activity towards that substrate.

Table S9. Summary of the most important interactions between the carboxylate fragment of PRA (atoms O1 and O4), the arginine residue on loop 5 (R169 in *Se*HisA, R143 in *Mt*PriA and R36 in *Tm*TrpF), and their surrounding residues, in various HisA, PriA and TrpF variants studied in this work.[a]

System	Donor/Acceptor	Donor Hydrogen	Fraction
*Se*HisA (Figure S7B)	PRA:O1…D176:OD2	D176:HD2	0.16
	PRA:O4…D176:OD2	D176:HD2	0.25
*Se*HisA(L169R) (Figure S7A)	PRA:O1…R169:NH2	R169:HH22	0.41
	PRA:O4…R169:NH2	R169:HH22	0.36
	PRA:O1…R169:NH1	R169:HH12	0.24
	PRA:O4…R169:NH1	R169:HH12	0.31
	D129:OD1…R169:NE	R169:HH21	0.41
	D129:OD2…R169:NH2	R169:HE	0.81
*Mt*PriA (Figure S5A)	PRA:O1…S81:OG	S81:HG	0.44
	PRA:O4…S81:OG	S81:HG	0.40
	PRA:O4…H49:NE2	H49:HE2	0.39
	PRA:O1…H49:NE2	H49:HE2	0.37
	D130:OD1…R143:NH2	R143:HH22	0.58
	D130:OD2…R43:NE	R143:HE	0.45
	D175:OD1…R143:NH1	R143:HH12	0.42
	D130:OD2…R143:NH2	R143:HH22	0.36
	D130:OD2…R143:N	R143:H	0.32
	D130:OD1…R143:NE	R143:HE	0.28
	D130:OD1…R143:N	R143:H	0.18
*Mt*PriA(R143A) (Figure S5B)	PRA:O1…H49:NE2	H49:HE2	0.31
	PRA:O4…S81:OG	S81:HG	0.34
	PRA:O1…S81:OG	S81:HG	0.31
	PRA:O4…H49:NE2	H49:HE2	0.26
*Tm*TrpF (Figure S6A)	PRA:O1…D126:OD2	D126:HD2	0.10
	PRA:O1…R36:NE	R36:HE	0.09
	PRA:O1…R36:NH2	R36:HH22	0.07
	PRA:O4…R36:NE	R36:HE	0.07
	PRA:O4…R36:NH2	R36:HH22	0.05
	E184:OE1…R36:NH1	R36:HH11	0.23
	E184:OE2…R36:NH2	R36:HH21	0.21
	E184:OE2…R36:NH1	R36:HH11	0.19
*Se*HisA(dup13-15/D10G/G102A/Q24L) (Figure S6B)	PRA:O1…R15b:NH2	R15b:HH21	0.33
	PRA:O4…R15b:NE	R15b:HE	0.22
	PRA:O1…D176:OD2	D176:HD2	0.21
	PRA:O4…D176:OD2	D176:HD2	0.16
	PRA:O1…R15b:NE	R15b:HE	0.14
	PRA:O4…R15b:NH2	R15b:HH21	0.11

[a] The analysis was performed with CPPTRAJ[33] as implemented in AmberTools19,[34] using a cutoff of 3.0 Å for the distance between the acceptor atom and the hydrogen of the donor atom (DonorH). The hydrogen bond occupancy is shown as a fraction of total simulation time, where a value of 1.0 indicates that the interaction was stable throughout the full simulation time, and a value of 0.0 indicates that no interaction was observed during the simulation. The figures referenced in the table illustrate the corresponding interactions in each system.

Table S10. A comparison of experimental and calculated activation free energies for the rate-limiting step of the isomerization of ProFAR by wild-type *Se*HisA, *Mt*PriA and variants.[a]

System	$\Delta G^{\ddagger}_{exp}$ [b]	$\Delta G^{\ddagger}_{calc}$ [c]	$\Delta\Delta G^{\ddagger}_{calc}$ [d]	$\Delta\Delta G^{\ddagger}_{exp,}$ [e]
***Se*HisA**				
Wild-Type$_{open}$	16.4	17.6 ± 0.6	0.1	-
Wild-Type$_{closed}$		17.5 ± 0.6	0.0	0.0
D10G	16.2	17.5 ± 0.4	0.0	-0.2
D129N	17.8	18.7 ± 0.4	1.2	1.4
S202A	17.6	16.1 ± 0.5	-1.4	1.2
***Mt*PriA**				
Wild-Type	18.3	18.3 ± 0.5	0.0	0.0
R19A	19.0	19.9 ± 0.4	1.6	0.7
D130A	20.2	18.2 ± 0.7	-0.1	1.9

[a] All values are shown in kcal mol^{-1}. The subscripts "open" and "closed" denote the open and closed conformations of loop 1. '-' indicates data not available. [b] $\Delta G^{\ddagger}_{exp}$ denotes experimental activation free energies, obtained from k_{cat} values presented in refs. [4, 6] and **Table S2** using transition state theory. These values provide an upper limit for the activation free energy of the ring-opening reaction. [c] $\Delta G^{\ddagger}_{calc}$ denotes calculated activation free energies, shown as average values and standard error of the mean over 30 individual EVB trajectories per system. [d] $\Delta\Delta G^{\ddagger}_{calc}$ denotes the difference between the calculated activation free energies for the wild-type and each enzyme variant studied in this work. [e] $\Delta\Delta G^{\ddagger}_{exp}$ denotes the difference between the experimental activation free energies for the wild-type and each enzyme variant studied in this work.

Table S11. A comparison of experimental and calculated activation free energies for the rate-limiting step of the isomerization of PRA by wild-type *Se*HisA, *Mt*PriA and variants.[a]

System	$\Delta G^{\ddagger}_{exp}$ [b]	$\Delta G^{\ddagger}_{calc}$ [c]	$\Delta\Delta G^{\ddagger}_{calc}$ [d]	$\Delta\Delta G^{\ddagger}_{exp,}$ [e]
Se**HisA**				
Wild-Type	-	19.5±0.6	-2.1	-
dup13-15	18.6	19.9±1.2	0.0	0.0
dup13-15/D10G	18.9	19.5±0.5	-0.4	0.3
dup13-15/D10G/G102A	17.9	18.5±1.0	-1.4	-0.7
dup13-15/D10G/G102A/Q24L	17.8	18.9±1.0	1.0	-0.8
dup13-15/D10G/G102A/Q24L/V15[b]M_{open}	16.7	21.2±1.2	1.7	-
dup13-15/D10G/G102A/Q24L/V15[b]M_{closed}		17.5±0.6	-2.4	-1.9
Mt**PriA**				
Wild-$Type_{open}$	16.7	23.3±1.2	5.0	-
Wild-$Type_{closed}$		16.7±0.7	0.0	0.0
R19A	17.4	17.4±0.6	0.7	0.7
D130A	17.2	12.8±0.5	-3.9	0.5
R143A	-	14.8±0.7	-1.9	-

[a] All values are shown in kcal mol^{-1}. The subscripts "open" and "closed" denote the open and closed conformations of loop 1. '-' indicates data not available. [b] $\Delta G^{\ddagger}_{exp}$ denotes experimental activation free energies, obtained from k_{cat} values presented in refs. [4, 6] and **Table S2** using transition state theory. These values provide an upper limit for the activation free energy of the ring-opening reaction. [c] $\Delta G^{\ddagger}_{calc}$ denotes calculated activation free energies, shown as average values and standard error of the mean over 30 individual EVB trajectories per system. [d] $\Delta\Delta G^{\ddagger}_{calc}$ denotes the difference between the calculated activation free energies for the wild-type and each enzyme variant studied in this work. [e] $\Delta\Delta G^{\ddagger}_{exp}$ denotes the difference between the experimental activation free energies for the wild-type and each enzyme variant studied in this work. Note that in the case of HisA, as the wild-type form of this enzyme is not able to isomerize PRA, we have instead used HisA(dup13-15), which does show TrpF-activity towards PRA,[6] as our reference state.

Table S12. Calculated distances at the Michaelis complexes (RS), transition states (TS) and intermediate states (IS) for the ring opening step of ProFAR catalyzed by HisA and PriA obtained from our EVB simulations.[a]

System			RS	TS	IS
HisA	$\mathbf{WT_{closed}}$	O_{Asp}-H	0.96	1.11	1.69
		H-OS_{ProFAR}	2.54	1.22	0.98
		OS_{ProFAR}-CT_{ProFAR}	1.29	1.63	2.36
	$\mathbf{WT_{open}}$	O_{Asp}-H	0.97	1.11	1.91
		H-OS_{ProFAR}	2.68	1.22	0.98
		OS_{ProFAR}-CT_{ProFAR}	1.29	1.63	2.40
	**D10G**	O_{Asp}-H	0.97	1.11	1.91
		H-OS_{ProFAR}	2.53	1.22	0.98
		OS_{ProFAR}-CT_{ProFAR}	1.29	1.63	2.40
	**D129N**	O_{Asp}-H	0.96	1.15	1.68
		H-OS_{ProFAR}	2.21	1.18	0.99
		OS_{ProFAR}-CT_{ProFAR}	1.29	1.66	2.22
	**S202A**	O_{Asp}-H	0.96	1.12	1.74
		H-OS_{ProFAR}	2.24	1.20	0.98
		OS_{ProFAR}-CT_{ProFAR}	1.30	1.65	2.34
PriA	**WT**	O_{Asp}-H	0.96	1.12	1.64
		H-OS_{ProFAR}	2.66	1.20	0.98
		OS_{ProFAR}-CT_{ProFAR}	1.29	1.63	2.39
	**R19A**	O_{Asp}-H	0.97	1.12	1.61
		H-OS_{ProFAR}	3.34	1.21	0.99
		OS_{ProFAR}-CT_{ProFAR}	1.27	1.63	2.34
	**D130A**	O_{Asp}-H	0.97	1.12	1.59
		H-OS_{ProFAR}	3.04	1.21	0.99
		OS_{ProFAR}-CT_{ProFAR}	1.29	1.64	2.34

[a] O_{Asp}-H denotes the distance between the proton and the relevant oxygen atom of the catalytic aspartic acid side chain, H-OS_{ProFAR} denotes the distance between the leaving group oxygen of ProFAR ring and the proton being transferred from the catalytic aspartic acid side chain, and OS_{ProFAR}-CT_{ProFAR} denotes the ring opening distance of ProFAR. All distances are shown in Å. Data is presented as average values over 30 individual EVB trajectories per system. All standard error of the mean values are 0.01 Å or less, and have therefore been omitted for clarity.

Table S13. Calculated distances at the Michaelis complexes (RS), transition states (TS) and intermediate states (IS) for the ring opening step of PRA catalyzed by HisA and PriA obtained from out EVB simulations.[a]

System			RS	TS	IS
**HisA**	**WT**	O_{Asp}-H	0.96	1.16	1.58
		H-OS_{PRA}	2.37	1.17	1.00
		OS_{PRA}-CT_{PRA}	1.43	1.73	2.22
	**dup13-15**	O_{Asp}-H	0.97	1.14	1.60
		H-OS_{PRA}	2.76	1.19	1.00
		OS_{PRA}-CT_{PRA}	1.43	1.70	2.24
	**dup13-15/D10G**	O_{Asp}-H	0.97	1.17	1.91
		H-OS_{PRA}	2.62	1.16	0.99
		OS_{PRA}-CT_{PRA}	1.43	1.74	2.31
	**dup13-15/D10G/G102A**	O_{Asp}-H	0.97	1.15	1.77
		H-OS_{PRA}	2.40	1.17	1.00
		OS_{PRA}-CT_{PRA}	1.42	1.70	2.20
	**dup13-15/D10G/G102A/Q24L**	O_{Asp}-H	0.97	1.15	1.74
		H-OS_{PRA}	2.57	1.18	1.00
		OS_{PRA}-CT_{PRA}	1.43	1.71	2.24
	**dup13-15/D10G/G102A/Q24L/V15[b]Mclose**	O_{Asp}-H	0.97	1.15	1.82
		H-OS_{PRA}	2.60	1.17	0.99
		OS_{PRA}-CT_{PRA}	1.42	1.70	2.26
	**dup13-15/D10G/G102A/Q24L/V15[b]Mopen**	O_{Asp}-H	0.97	1.16	1.86
		H-OS_{PRA}	2.71	1.16	0.99
		OS_{PRA}-CT_{PRA}	1.42	1.70	2.17
**PriA**	**WTclose**	O_{Asp}-H	0.96	1.11	1.62
		H-OS_{PRA}	2.39	1.22	0.99
		OS_{PRA}-CT_{PRA}	1.43	1.68	2.30
	**WTopen**	O_{Asp}-H	0.97	1.14	1.63
		H-OS_{PRA}	2.80	1.18	1.00
		OS_{PRA}-CT_{PRA}	1.43	1.71	2.25
	**R19A**	O_{Asp}-H	0.97	1.12	1.59
		H-OS_{PRA}	3.16	1.21	1.00
		OS_{PRA}-CT_{PRA}	1.42	1.69	2.32

**D130A**	O_{Asp}-H	0.97	1.11	1.62
	H-OS_{PRA}	1.96	1.21	0.99
	OS_{PRA}-CT_{PRA}	1.44	1.69	2.30
**R143A**	O_{Asp}-H	0.96	1.11	1.59
	H-OS_{PRA}	2.46	1.21	0.99
	OS_{PRA}-CT_{PRA}	1.43	1.68	2.33

[a] O_{Asp}-H denotes the distance between the proton and the relevant oxygen atom of the catalytic aspartic acid side chain, H-OS_{PRA} denotes the distance between the leaving group oxygen of PRA ring and the proton being transferred from the catalytic aspartic acid side chain, and OS_{PRA}-CT_{PRA} denotes the ring opening distance of PRA. All distances are shown in Å. Data is presented as average values over 30 individual EVB trajectories per system. All standard error of the mean values are 0.01 Å or less, and have therefore been omitted for clarity.

Table S14. List of neutralized residues and histidine protonation patterns used in our EVB simulations of *Se*HisA, *Mt*PriA, and variants, in complex with substrates ProFAR and PRA.[a]

Residue Type	Residue Number
Asp[b]	HisA: 110, 176<br>PriA: 75, 87, 175, 194
Glu	HisA: 85, 86, 117<br>PriA: 65, 69, 88, 120, 157, 162
Lys	HisA: 66, 93, 113, 236<br>PriA: 73
Arg	HisA: 118, 193<br>PriA: 43, 116
His-ε	HisA: 17, 47, 135, 167<br>PriA: 64, 121, 138
His-δ	PriA: 217
His-δ and ε	PriA: 50

[a] Shown here are the residues that fall outside the explicit simulation sphere, and were thus kept in their neutral form to avoid system instabilities created by having charged residues outside the water droplet (this is standard practice for such simulations). All other residues were kept in their usual ionization state at physiological pH. In the case of the histidine side chains, His-ε and His-δ indicate histidine side chains protonated at the $N_{\varepsilon 2}$ and $N_{\delta 1}$ nitrogen atoms, respectively, and His- δ and His-ε indicates a doubly protonated histidine side chain. [b] Note that D176 in HisA and the corresponding D175 in PriA are protonated in our simulations, as they are the respective catalytic acids in the reaction shown in **Figure 1**.

Table S15. EVB parameters used to describe the opening of the ribose rings of substrates ProFAR and PRA, as catalyzed by the various enzymes studied in this work. [a]

	H_{ij}	α_i
PriA/ProFAR	36.5	80.2
PriA/PRA	28.4	43.2

[a] The EVB off-diagonal term, H_{ij}, and gas-phase shift, α_i, were fit to reproduce the activation free energies derived from the turnover numbers for the PriA-catalyzed isomerization of substrates ProFAR and PRA, respectively, as described in the main text. The same parameters were then used unchanged to describe all systems. For a detailed description of the physical meaning of the EVB parameters, see *e.g.* refs. [24, 25].

S4. Supplementary References

1. Hennig, M.; Sterner, R.; Kirschner, K.; Jansonius, J. N., Crystal Structure at 2.0 Å Resolution of Phosphoribosyl Anthranilate Isomerase from the Hyperthermophile *Thermotoga Maritima*: Possible Determinants of Protein Stability. *Biochemistry* **1997,** *36*, 6009-6016.

2. Berman, H. M.; Westbrook, J.; Feng, Z.; Gililand, G.; Bhat, T. N.; Weissig, H.; Shindyalov, I. N.; Bourne, P. E., The Protein Data Bank. *Nucleic Acids Res.* **2000,** *28*, 235-242.

3. Henn-Sax, M.; Thoma, R.; Schmidt, S.; Hennig, M.; Kirschner, K.; Sterner, R., Two $(\beta\alpha)_8$-Barrel Enzymes of Histidine and Tryptophan Biosynthesis Have Similar Reaction Mechanisms and Common Strategies for Protecting Their Labile Substrates. *Biochemistry* **2002,** *41*, 12032-12042.

4. Due, A. V.; Kuper, J.; Geerlof, A.; von Kries, J. P.; Wilmanns, M., Bisubstrate Specificity in Histidine/Tryptophan Biosynthesis Isomerase from *Mycobacterium Tuberculosis* by Active Site Metamorphosis. *Proc. Natl. Acad. Sci. USA* **2011,** *108*, 3554-3559.

5. Söderholm, A.; Guo, X.; Newton, M. S.; Evans, G. B.; Näsvall, J.; Patrick, W. M.; Selmer, M., Two-step Ligand Binding in a $(\beta\alpha)_8$ Barrel Enzyme: Substrate-Bound Structures Shed New Light on the Catalytic Cycle of HisA. *J. Biol. Chem.* **2015,** *290*, 24657-24668.

6. Newton, M. S.; Guo, X.; Söderholm, A.; Näsvall, J.; Lundström, P.; Andersson, D. I.; Selmer, M.; Patrick, W. M., Structural and Functional Innovations in the Real-Time Evolution of New $(\beta\alpha)_8$ Barrel Enzymes. *Proc. Natl. Acad. Sci. USA* **2017,** *114*, 4727-4732.

7. Shapovalov, M. X.; Dunbrack Jr., R. L., A Smoothed Backbone-Dependent Rotamer Library for Proteins Derived from Adaptive Kernel Density Estimates and Regressions. *Structure* **2011,** *19*, 844-858.

8. Pettersen, E. F.; Goddard, T. D.; Huang, C. C.; Couch, G. S.; Greenblatt, D. M.; Meng, E. C.; Ferrin, T. E., UCSF Chimera - A Visualization System for Exploratory Research and Analysis. *J. Comp. Chem.* **2004,** *25*, 1605-1612.

9. Šali, A.; Blundell, T. L., Comparative Protein Modelling by Satisfaction of Spatial Restraints. *J. Mol. Biol.* **1993,** *234*, 779-815.

10. Olsson, M. H. M.; Søndergaard, C. R.; Rostkowski, M.; Jensen, J. H., PROPKA3: Consistent Treatment of Internal and Surface Residues in Empirical pK_a Predictions. *J. Chem. Theory Comput.* **2011,** *7Th* 525-537.

11. Schrödinger LLC, *Maestro, Schrödinger Release 2021-4*. New York, 2021.

12. Wang, J.; Wang, W.; Kollman, P. A.; Case, D. A., Automatic Atom Type and Bond Type Perception in Molecular Mechanical Calculations. *J. Mol. Graph. Model.* **2006,** *25*, 247-260.

13. Frisch, M. J.; Trucks, G. W.; Schlegel, H. B.; Scuseria, G. E.; Robb, M. A.; Cheeseman, J. R.; Scalmani, G.; Barone, V.; Petersson, G. A.; Nakatsuji, H.; Li, X.; Caricato, M.; Marenich, A.; Bloino, J.; Janesko, B. G.; Gomperts, R.; Mennucci, B.; Hratchian, H. P.; Ortiz, J. V.; Izmaylov, A. F.; Sonnenberg, J. L.; Williams-Young, D.; Ding, F.; Lipparini, F.; Egidi, F.; Goings, J.; Peng, B.; Petrone, A.; Henderson, T.; Ranansinghe, D.; Zakrzewski, V. G.; Gao, J.; Rega, N.; Zheng, G.; Liang, W.; Hada, M.; Ehara, M.; Toyota, K.; Fukuda, R.; Hasegawa, J.; Ishida, M.; Nakajima, T.; Honda, Y.; Kitao, O.; Nakai, H.; Vreven, T.; Throssel, l., K.; Montgomery Jr., J. A.; Peralta, J. E.; Ogliaro, F.; Bearpark, M.; Heyd, J. J.; Brothers, E.; Kudin, K. N.; Staroverov, V. N.; Keith, T.; Kobayashi, R.; Normand, J.; Raghavachari, K.; Rendell, A.; Burant, J. C.; Iyengar, S. S.; Tomasi, J.; Cossi, M.; Millam, J. M.; Klene, M.; Adamo, C.; Cammi, R.; Ochterski, J. W.; Martin, R. L.; Morokuma, K.; Farkas, O.; Foresman, J. B.; Fox, D. J., *Gaussian 09, Revision E.01*. Gaussian, Inc.: Wallingford, CT, 2016.

14. Wang, J.; Wolf, R. M.; Caldwell, J. W.; Kollmann, P. A.; Case, D. A., Development and Testing of a General AMBER Force Field. *J. Comput. Chem.* **2004,** *25*, 1157-1174.

15. Jorgensen, W. L.; Maxwell, D. S.; Tirado-Rives, J., Development and Testing of the OPLS All-Atom Force Field on Conformational Energetics and Properties of Organic Liquids. *J. Am. Chem. Soc.* **1996,** *118*, 11225-11236.

16. Case, D. A.; Betz, R. M.; Cerutti, D. S.; Cheatham III, T. E.; Darden, T. A.; Duke, R. E.; Giese, T. J.; Gohlke, H.; Goetz, A. W.; Homeyer, N.; Izadi, S.; Janowski, P.; Kaus, J.; Kovalenko, A.; Lee, T. S.; LeGrand, S.; Li, P.; Lin, C.; Luchko, T.; Luo, R.; Madej, B.; Mermelstein, D.; Merz, K. M.; Monard, G.; Nguyen, H.; Nguyen, H. T.; Omelyan, I.; Onufriev, A.; Roe, D. R.; Roitberg, A.; Sagui, C.; Simmerling, C. L.; Botello-Smith, W. M.; Swails, J.; Walker, R. C.; Wang, J.; Wolf, R. M.; Wu, X.; Xiao, L.; Kollman, P. A., *AMBER 2016*. University of California: San Francisco, 2016.

17. Maier, J. A.; Martinez, C.; Kasavaghala, K.; Wickström, L.; Hauser, K. E.; Simmerling, C. L., FF14SB: Improving the Accuracy of Protein Side Chain and Backbone Parameters from FF99SB. *J. Chem. Theory Comput.* **2015,** *11*, 3696-3713.

18. Berendsen, H. J. C.; Postma, J. P. M.; van Gunsteren, W. F.; DiNola, A.; Haak, J. R., Molecular-Dynamics with Coupling to an External Bath. *J. Chem. Phys.* **1984,** *81*, 3684-3690.

19. Schneider, T.; Stoll, E., Molecular-Dynamics Study of a Three-Dimensional One-Component Model for Distortive Phase Transitions. *Phys. Rev. B* **1978,** *17*, 1302-1322.

20. Hess, B., A Parallel Linear Constraint Solver for Molecular Simulation. *J. Chem. Theory Comput.* **2008,** *4*, 116-122.

21. Darden, T.; York, D.; Pedersen, L., Particle Mesh Ewald: An N ·log(N) Method for Ewald Sums in Large Systems. . *J. Chem. Phys.* **1993,** *98*, 10089-10092.

22. Jorgensen, W. L.; Chandresekhar, J.; Madura, J. D., Comparison of Simple Potential Functions for Simulating Liquid Water. *J. Chem. Phys.* **1983,** *79*, 926.

23. Warshel, A.; King, G., Polarization Constraints in Molecular Dynamics Simulation of Aqueous Solutions: The Surface Constraint All Atom Solvent (SCAAS) Model. *Chem. Phys. Lett.* **1985,** *121*, 124-129.

24. Warshel, A.; Weiss, R. M., An Empirical Valence Bond Approach for Comparing Reactions in Solutions and in Enzymes. *J. Am. Chem. Soc.* **1980,** *102*, 6218-6226.

25. Shurki, A.; Derat, E.; Barrozo, A.; Kamerlin, S. C. L., How Valence Bond Theory can Help You Understand Your (Bio)chemical Reaction. *Chem. Soc. Rev.* **2015,** *44*, 1037-1052.

26. Schrödinger LLC, *Schrödinger Release 2017-1: MacroModel*. New York, 2017.

27. Marelius, J.; Kolmodin, K.; Feierberg, I.; Åqvist, J., Q: A Molecular Dynamics Program for Free Energy Calculations and Empirical Valence Bond Simulations in Biomolecular Systems. *J. Mol. Graph. Model.* **1998,** *16*, 213-225.

28. Bauer, P.; Barrozo, A.; Purg, M.; Amrein, B. A.; Esguerra, M.; Wilson, P. B.; Major, D. T.; Åqvist, J.; Kamerlin, S. C. L., *Q6*: A Comprehensive Toolkit for Empirical Valence Bond and Related Free Energy Calculations. *SoftwareX* **2018,** *7*, 388-395.

29. Ryckaert, J.-P.; Ciccotti, G.; Berendsen, H. J. C., Numerical Integration of the Cartesian Equations of Motion of a System with Constraints: Molecular Dynamics of *n*-Alkanes. *J. Comput. Phys.* **1977,** *23*, 327-341.

30. Lee, F. S.; Warshel, A., A Local Reaction Field Method for Fast Evaluation of Long-range Electrostatic Interactions in Molecular Simulations. *J. Chem. Phys.* **1992,** *97*, 3100-3107.

31. Schmidtke, P.; Bidon-Chanal, A.; Luque, F. J.; Barril, X., MDpocket: Open-Source Cavity Detection and Characterization on Molecular Dynamics Trajectories. *Bioinformatics* **2011,** *27*, 3276-3285.

32. Le Guilloux, V.; Schmidtke, P.; Tuffery, P., Fpocket: An Open Source Platform for Ligand Pocket Detection. *BMC Bioinformat.* **2009,** *10*, 168.

33. Roe, D. R.; Cheatham III, T. E., PTRAJ and CPPTRAJ: Software for Processing and Analysis of Molecular Dynamics Trajectory Data. *J. Chem. Theory Comput.* **2013,** *9*, 3084-3095.

34. Case, D. A.; Ben-Shalom, I. Y.; Brozell, S. R.; Cerutti, D. S.; Cheatham III, T. E.; Cruzeiro, V. W. D.; Darden, T. A.; Duke, R. E.; Ghoreishi, D.; Giambasu, G.; Giese, T.; Gilson, M. K.; Gohlke, H.; Goetz, A. W.; Greene, D.; Harris, R.; Homeyer, N.; Huang, Y.; Izadi, S.; Kovalenko, A.; Krasny, R.; Kurtzman, T.; Lee, T. S.; LeGrand, S.; Li, P.; Lin, C.; Liu, J.; Luchko, T.; Luo, R.; Man, V.;

Mermelstein, D. J.; Merz, K. M.; Miao, Y.; Monard, G.; Nguyen, C.; Nguyen, H.; Onufriev, A.; Pan, F.; Qi, R.; Roe, D. R.; Roitberg, A.; Sagui, C.; Schott-Verdugo, S.; Shen, J.; Simmerling, C. L.; Smith, J.; Swails, J.; Walker, R. C.; Wang, J.; Wei, H.; Wilson, L.; Wolf, R. M.; Wu, X.; Xiao, L.; Xiong, Y.; York, D. M.; Kollman, P. A., *AMBER 2019*. University of California: San Francisco, 2019.

35. Sterner, R.; Kleemann, G. R.; Szadkowski, H.; Lustig, A.; Hennig, M.; Kirschner, K., Phosphoribosyl Anthranilate Isomeraes from *Thermotoga Maritima* is an Extremely Stable and Active Homodimer. *Prot. Sci.* **1996,** *5*, 2000-2008.

36. Näsvall, J.; Sun, L.; Roth, J. R.; Andersson, D. I., Real-Time Evolution of New Genes by Innovation, Amplification and Divergence. *Science* **2012,** *338*, 384-387.
